# Impacts of Degradation on Water, Energy, and Carbon Cycling of the Amazon Tropical Forests

**DOI:** 10.1029/2020JG005677

**Published:** 2020-08-20

**Authors:** Marcos Longo, Sassan Saatchi, Michael Keller, Kevin Bowman, António Ferraz, Paul R. Moorcroft, Douglas C. Morton, Damien Bonal, Paulo Brando, Benoît Burban, Géraldine Derroire, Maiza N. dos‐Santos, Victoria Meyer, Scott Saleska, Susan Trumbore, Grégoire Vincent

**Affiliations:** ^1^ Jet Propulsion Laboratory California Institute of Technology Pasadena CA USA; ^2^ Institute of Environment and Sustainability University of California Los Angeles CA USA; ^3^ International Institute of Tropical Forestry USDA Forest Service Rio Piedras Puerto Rico; ^4^ Embrapa Informática Agropecuária Campinas Brazil; ^5^ Department of Organismic and Evolutionary Biology Harvard University Cambridge MA USA; ^6^ NASA Goddard Space Flight Center Greenbelt MD USA; ^7^ Université de Lorraine, INRAE, AgroParisTech, UMR Silva Nancy France; ^8^ Department of Earth System Science University of California Irvine CA USA; ^9^ Woods Hole Research Center Woods Hole MA USA; ^10^ Instituto de Pesquisa Ambiental da Amazônia Brasília Brazil; ^11^ Institut National de Recherche en Agriculture, Alimentation et Environnement (INRAE), UMR 0745 EcoFoG, Campus Agronomique Kourou France; ^12^ Centre de Coopération Internationale en Recherche Agronomique pour le Développement (CIRAD), UMR EcoFoG (Agroparistech, CNRS, INRAE, Université des Antilles, Université de Guyane), Campus Agronomique Kourou France; ^13^ Ecology and Evolutionary Biology University of Arizona Tucson AZ USA; ^14^ Max‐Planck‐Institut für Biochemie Jena Germany; ^15^ AMAP, Univ Montpellier, IRD, CIRAD, CNRS, INRAE Montpellier France

**Keywords:** Amazon, remote sensing, ecosystem modeling, forest degradation, evapotranspiration, drought

## Abstract

Selective logging, fragmentation, and understory fires directly degrade forest structure and composition. However, studies addressing the effects of forest degradation on carbon, water, and energy cycles are scarce. Here, we integrate field observations and high‐resolution remote sensing from airborne lidar to provide realistic initial conditions to the Ecosystem Demography Model (ED‐2.2) and investigate how disturbances from forest degradation affect gross primary production (GPP), evapotranspiration (ET), and sensible heat flux (H). We used forest structural information retrieved from airborne lidar samples (13,500 ha) and calibrated with 817 inventory plots (0.25 ha) across precipitation and degradation gradients in the eastern Amazon as initial conditions to ED‐2.2 model. Our results show that the magnitude and seasonality of fluxes were modulated by changes in forest structure caused by degradation. During the dry season and under typical conditions, severely degraded forests (biomass loss ≥66%) experienced water stress with declines in ET (up to 34%) and GPP (up to 35%) and increases of H (up to 43%) and daily mean ground temperatures (up to 6.5°C) relative to intact forests. In contrast, the relative impact of forest degradation on energy, water, and carbon cycles markedly diminishes under extreme, multiyear droughts, as a consequence of severe stress experienced by intact forests. Our results highlight that the water and energy cycles in the Amazon are driven by not only climate and deforestation but also the past disturbance and changes of forest structure from degradation, suggesting a much broader influence of human land use activities on the tropical ecosystems.

## Key Points

- Airborne lidar can be used to inform about degradation‐driven changes in structure to vegetation models
- Forest degradation typically depletes evapotranspiration and productivity and increases flammability
- Extreme droughts reduce functional differences between degraded and intact tropical forests

## 1 Introduction

Tropical forests account for 25–40% of total carbon stocks in terrestrial ecosystems (Meister et al., 2012; Sabine et al., 2004), but their maintenance and functioning have been weakened by climate and land use change. As a result, tropical forests may shift to net sources of carbon to the atmosphere, with residence time of carbon in forests declining by 50% (Davidson et al., 2012; Erb et al., 2016; Grace et al., 2014; Lewis et al., 2015). Land use and land cover changes contribute to nearly 15% of total annual carbon emissions (Friedlingstein et al., 2019; Harris et al., 2012). However, most studies assessing the effects of land use change on tropical forest stocks and fluxes have focused on the effects of deforestation (e.g., Achard et al., 2014; Harris et al., 2012). Logging, understory fires, and forest fragmentation—collectively known as *forest degradation* (Hosonuma et al., 2012)—could play a comparable role in the forest's energy, water, and carbon cycle and induce locally warmer and drier conditions that could be detrimental to their functioning (Grossiord et al., 2020; Sullivan et al., 2020), but these effects remain poorly quantified.

Significant fractions of the remaining tropical forests are located within 1 km from the forest's edge (Haddad et al., 2015; Lewis et al., 2015) and thus are probably degraded (Asner et al., 2006; Morton et al., 2013; Potapov et al., 2017; Pütz et al., 2014; Tyukavina et al., 2016). The area impacted by forest degradation in the Amazon each year is highly uncertain but likely comparable to deforestation (Asner et al., 2006; Morton et al., 2013; Tyukavina et al., 2017). Total carbon losses attributable to degradation may be similar to or exceed deforestation‐related losses in tropical forests (Aragão et al., 2018; Baccini et al., 2017; Berenguer et al., 2014; Erb et al., 2018; Pearson et al., 2017), and degradation may even dominate the carbon losses in indigenous lands and protected areas (Walker et al., 2020). At the local scale, carbon stocks in degraded forests are extremely variable. Lightly disturbed forests (e.g., reduced‐impact logging) store as much carbon as intact forests, while forests impacted by severe or multiple disturbances may lose a significant fraction or nearly all of their original carbon stocks (Alamgir et al., 2016; Berenguer et al., 2014; Ferraz et al., 2018; Longo et al., 2016; Rappaport et al., 2018). Transitions between lightly and heavily degraded forests may be nonlinear and abrupt (Brando et al., 2014). Unquestionably, estimates of fluxes from forest degradation and regeneration are more uncertain than emissions from deforestation (Aragão et al., 2014; Bustamante et al., 2016; Morton, 2016), because their impacts on forests are more subtle than deforestation and thus more difficult to detect and quantify with traditional remote sensing techniques.

Selective logging and fires also modify the forest structure, composition, and functioning. For example, selective logging in the tropics generally targets large trees (diameter at breast height, DBH≥40–60 cm) from a few marketable species (e.g., Blanc et al., 2009; Feldpausch et al., 2005; Pinagé et al., 2019), but the other logging structures such as skid trails and log decks kill or damage mostly small trees (DBH<20 cm) (Feldpausch et al., 2005). Likewise, fire mortality decreases with tree size and the bark thickness (e.g., Brando et al., 2012; Pellegrini et al., 2016), although areas disturbed by recurrent fires also show significant losses of large trees (Barlow et al., 2003; Brando, Silvério, et al., 2019; Martins et al., 2012; Silvério et al., 2019). Consequently, degradation creates more open canopies and thinner understory (e.g., d'Oliveira et al., 2012; Pinagé et al., 2019; Silvério et al., 2019) and increased abundance of grasses and fast‐growing, low‐wood‐density tree species (Barlow et al., 2016; Both et al., 2019; Brando, Silvério, et al., 2019).

Previous studies indicate an increase in dry‐season length in parts of the Amazon where both deforestation and forest degradation are pervasive (e.g., Fu et al., 2013; Sena et al., 2018) and that the onset of the wet season is modulated by forest transpiration (J. S. Wright et al., 2017). Temperature and vapor pressure deficit (VPD), important drivers of evapotranspiration (ET), were found by Kapos (1989) to be significantly higher near forest edges. Likewise, Jucker et al. (2018) installed a network of micrometeorological measurements across a study area in Sabah, Malaysia, that included intact forests, a broad range of degraded forests, and oil palm plantations and found that forest structure, along with topographic features, explained most of the variance in understory temperature. Yet, only a few studies on experimental sites quantified the magnitude, seasonality, and interannual variability of water and energy cycles in degraded forests. For example, Miller et al. (2011) analyzed the impact of reduced‐impact, low‐intensity selective logging in the Amazon using eddy covariance towers and found only minor impacts of logging on sensible and latent heat fluxes. Recently, Brando, Silvério, et al. (2019) compared eddy covariance data from two towers at an experimental fire site in the Amazon forest and found declining differences in gross primary productivity (GPP) and small differences in ET between the control and burned area between 4 and 8 yr after the last burn.

Field inventory plots are fundamental to sample the structure and species composition of tropical forests, but they also have important limitations to characterize the heterogeneity of degraded landscapes. First, the number of plots required to characterize stands increase with heterogeneity, often reaching impractical numbers (Marvin et al., 2014). In addition, most tropical forest degradation occurs in private landholdings and privately managed logging concessions, where limited access by researchers may create sampling bias toward well‐managed areas, which generally experience less intensive degradation. However, airborne laser scanning (airborne lidar) can circumvent these limitations over large areas with submeter resolution. Airborne lidar data have been used successfully to quantify structural characteristics of the canopy such as height and leaf area distribution (Hunter et al., 2013; Shao et al., 2019; Vincent et al., 2017). Moreover, these data have also been used to quantify changes in canopy structure and carbon stocks at local to regional scale that experienced multiple levels of degradation (e.g., Asner et al., 2010; Ferraz et al., 2018; Longo et al., 2016; Meyer et al., 2019).

Numerical models can be used to understand the links between changes in forest structure and light and water availability for different local plant communities and the overall impact on energy, water, and carbon fluxes between forests and the atmosphere. In the past, *big‐leaf* models have been modified to account for the long‐term impacts of selectively logged tropical forests on the carbon cycle of tropical forests (e.g., Huang & Asner, 2010; Huang et al., 2008). However, big‐leaf models generally do not represent the mechanisms that control access and availability of light and water in complex and heterogeneous forest structures (Fisher et al., 2018; D. Purves & Pacala, 2008) (but see Braghiere et al., 2019). Individual‐based models can represent the changes in the population structure and microenvironments due to degradation (R. Fischer et al., 2016; Maréchaux & Chave, 2017), but the complexity and computational burden of these simulations often limit their application to single sites. Cohort‐based models, such as the Ecosystem Demography (ED‐2.2) model (Longo, Knox, Medvigy, Levine, Dietze, Kim, et al., 2019; Medvigy et al., 2009), strike a balance between these end‐members because they can efficiently represent the horizontal and vertical heterogeneity of forests. However, to represent the impact of heterogeneity in the energy, water, and carbon cycles, it is critical that these models are informed with realistic initial conditions that capture the landscape variability and they accurately represent the complex interactions between climate and the microenvironment variability. Previous studies using a variety of cohort‐based models have demonstrated that cohort‐based models can realistically reproduce the microenvironment heterogeneity and the long‐term dynamics of ecosystems, compared to both individual‐based models (Moorcroft et al., 2001; Strigul et al., 2008) and observations (Koven et al., 2019; Longo, Knox, Levine, et al., 2019; D. W. Purves et al., 2008).

In this study, we use airborne lidar data to quantify forest structure variability across the Amazon in order to provide critical initial conditions for ecosystem demography models. We also investigate the role of forest degradation on the Amazon forest productivity, flammability, and the degradation impacts on the water and energy cycles. Specifically, we seek to answer the following questions:

1. What are the relationships between degradation metrics (e.g., biomass loss) and changes in carbon, water, and energy fluxes, and how does it vary across seasons and regions with different rainfall regimes?
2. How do droughts affect the relationships between degradation and ecosystem functioning?
3. Does forest degradation make Amazon forests more susceptible to fires? If so, which parts of the Amazon experience the largest flammability response to degradation?

To this end, we integrate field inventory plots with high‐resolution airborne lidar data over five study regions in the eastern Amazon along a precipitation gradient and with a broad range of anthropogenic disturbance histories, to provide initial conditions to ED‐2.2 that realistically represent the structural diversity of degraded forests. While limited to specific regions in the Amazon where detailed degradation information exists, our goal is to provide a framework that can be extended to larger scales, including biome and pantropical scales.

## 2 Materials and Methods

### 2.1 Study Regions

**Figure 1:**
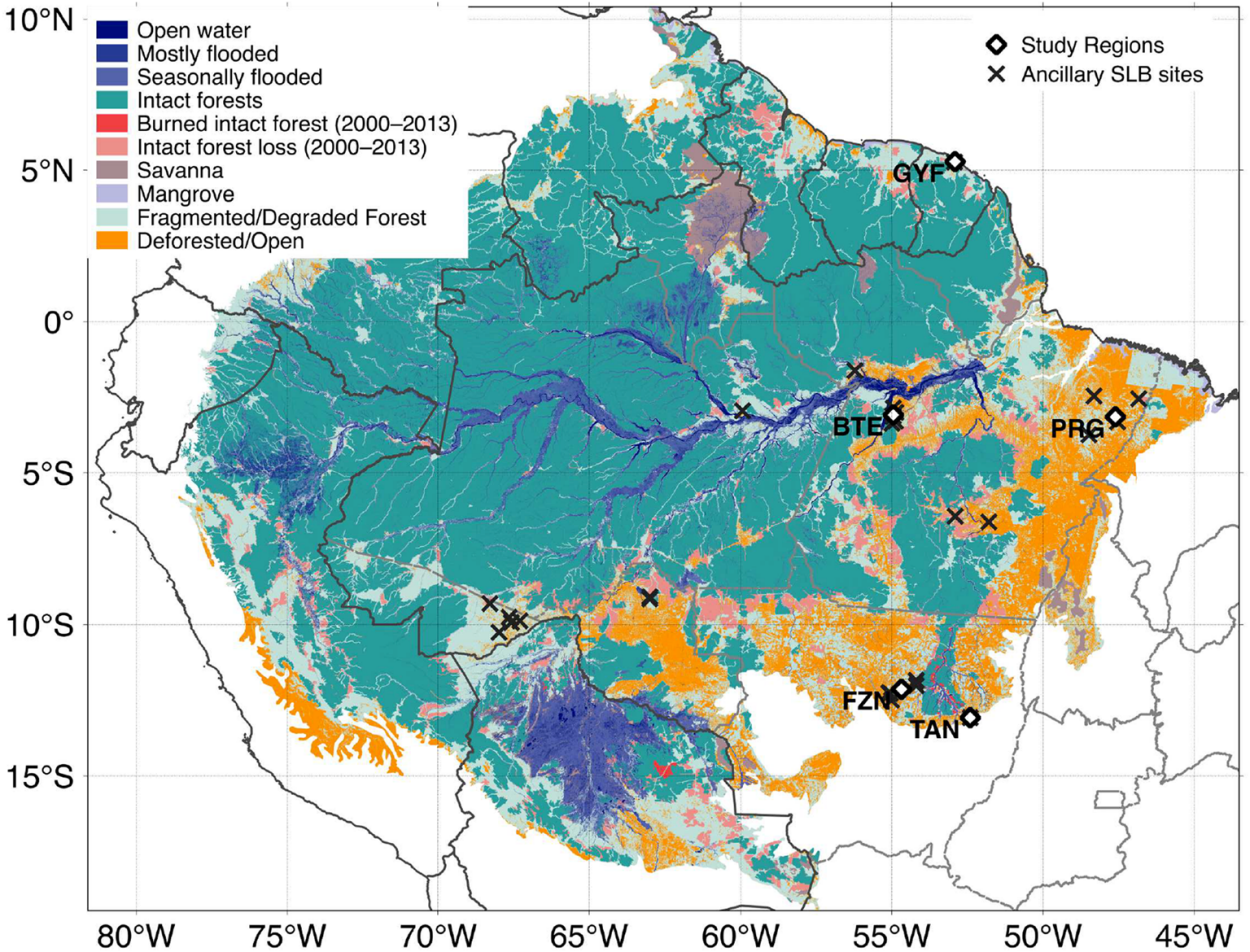
Location of the five study regions within the Amazon biome region, along with land classification as of 2013. Intact forest and intact forest loss were obtained from Potapov et al. (2017), open and deforested areas were obtained from PRODES‐INPE (2018) (Brazil), areas with tree cover below 20% are according to Hansen et al. (2013) (other countries), wetlands and water bodies in the Amazon River Basin were from Hess et al. (2015), and savannas and mangroves were obtained from Olson et al. (2001).

We selected five study regions across a gradient of disturbance and climate conditions where ground and airborne lidar are available to study the forest function (Figure 1 and Table 1). Three of these sites include eddy covariance tower measurement of energy, water, and carbon dioxide fluxes for comparison with the model simulations and have been the focus of several ecological studies in the past. Additional details on the disturbance history of each region are available in supporting information Text S1.

1. *Paracou, French Guiana (GYF)* is a field station where a logging experiment was conducted between 1987 and 1988 that includes intact forest controls and three selective logging treatments: timber extraction using conventional logging techniques, timber extraction and canopy thinning, and timber and fuelwood extraction followed by canopy thinning (Gourlet‐Fleury et al., 2004). The eddy covariance tower at the site is located in the undisturbed forest and has been operational since 2004 (Guyaflux Bonal et al., 2008).
2. *Belterra, Brazil (BTE)*. Over the past 100 yr, this region experienced cycles of economic growth and recession that created a complex landscape dominated by deforestation, degradation, and second growth. The Tapajós National Forest is this region and has areas of intact forests and selectively logged forests using reduced‐impact techniques (Lei et al., 2018; Pyle et al., 2008; VanWey et al., 2007). An eddy covariance tower known as Km 67 overlaps with one of the surveyed sites and has data for 2001–2005 and 2008–2011 (Hayek et al., 2018).
3. The *Paragominas, Brazil (PRG)* region used to be within the largest timber production area in Brazil and has undergone selective logging since the 1970s (Veríssimo et al., 1992). Since the 1990s, the economy has shifted toward agriculture, introducing large‐scale deforestation such that nearly half of the original forest cover has been lost, and most of the remaining areas have been logged (Pinto et al., 2009).
4. *Feliz Natal, Brazil (FZN)* is located at the southern fringe of the Amazon in a mosaic landscape of soybean fields, grazing lands, and logged forests. This region regularly experiences severe dry seasons and frequent understory fires (Morton et al., 2013; Rappaport et al., 2018).
5. *Tanguro, Brazil (TAN)* is located in an experimental fire study area within a larger landscape covered by intact forests and forests that were disturbed with low‐intensity understory fires (one, three, and six times) between 2004 and 2010 (Balch et al., 2008; Brando et al., 2014). The surveyed region also includes two eddy covariance towers that have been operating since 2014 at both the intact and burned forests (Brando, Silvério, et al., 2019).

**Table 1:** Overview of the Study Regions, Including Mean Annual Precipitation (MAP) and Dry‐Season Length (DSL)

		MAP^a^	DSL^b^	Lidar	Inventory	
Region (code)	Coordinates	(mm)	(mo)	(ha)	(ha)	Disturbances^c^
Paracou (GYF)	5.28°N; 52.91°W	3,040	2 (0)	963	79.8	INT, CL1, LTH
Belterra (BTE)	3.09°S; 54.95°W	1,890	5 (1)	4,057	16.7	INT, RIL, BN1, BN2, BN3
Paragominas (PRG)	3.15°S; 47.61°W	1,850	6 (2)	3,217	35.6	INT, RIL, CL1, BN1, LB1, BN2, BN3
Feliz Natal (FZN)	12.14°S; 54.68°W	1,940	5 (4)	4,210	14.0	INT, CL1, CL2, BN1, LB1, BN2, BN3
Tanguro (TAN)	13.08°S; 52.41°W	1,800	5 (4)	1,006	22.9	INT, BN1, BN3, BN6

a Source for mean annual precipitation (MAP) data: GYF—Gourlet‐Fleury, Ferry, et al. (2004); other regions—nearest site available at INMET (2019).

b Dry‐season length (DSL): number of months with precipitation below 100 mm; numbers in parentheses indicate number of severely dry months (precipitation below 30 mm).

c Disturbance history classes: INT = intact; RIL = reduced‐impact logging; CL*x* = conventional logging (*x* times); LTH = conventional logging and thinning; LB1 = conventional logging and burned (once); BN*x* = burned *x* times.

These five study regions were sampled at multiple sites by small‐footprint, multiple‐return airborne lidar. The lidar data provided both the terrain elevation at high spatial resolution (1 m) and detailed information about the vertical structure of forests from a uniform point cloud density to meet a minimum return density of four returns per m^2^ over 99.5% of the area (Leitold et al., 2015). Living trees of diameter at breast height DBH ≥ 10 cm were either botanically identified (experimental plots in GYF) or identified from field characteristics by local parataxonomists. To characterize the disturbance history, we used either published information from the experimental regions GYF (Bonal et al., 2008; Gourlet‐Fleury et al., 2004; Wagner et al., 2013) and TAN (Brando et al., 2012, 2014) or the disturbance history analysis from Longo et al. (2016), which was based on a visual interpretation of the Normalized Burn Ratio (NBR) of cloud‐free Landsat images since 1984 and complemented with information from logging companies for the reduced‐impact logging sites (e.g., Pinagé et al., 2019). Details on site‐specific data used in this study are available in Text S2 and previous work (Brando, Silvério, et al., 2019; Longo et al., 2016; Vincent et al., 2017) and were obtained through the Paracou Experimental Station and the Sustainable Landscapes Brazil data servers (dos‐Santos et al., 2019; Paracou Portal, 2016; Sustainable Landscapes Brazil, 2019).

### 2.2 Overview of the Modeling Framework

In this study, we used the Ecosystem Demography model, Version 2.2 (ED‐2.2) (Longo, Knox, Medvigy, Levine, Dietze, Kim, et al., 2019; Medvigy et al., 2009; Moorcroft et al., 2001) to simulate the impacts of forest structure on energy, water, and carbon cycles. For any point of interest, the ED‐2.2 model simulates the forest structure and functional diversity across a landscape and simulates the energy, water, and carbon budgets for multiple canopy environments, which represent the forest heterogeneity (Longo, Knox, Medvigy, Levine, Dietze, Kim, et al., 2019). ED‐2.2 has been successfully evaluated and used in both short‐term and long‐term studies in the Amazon forest (Levine et al., 2016; Longo, Knox, Levine, et al., 2019; Powell et al., 2013; Zhang et al., 2015). In ED‐2.2, the horizontal and vertical heterogeneities of forests are represented through a hierarchical structure. Each area with the same climate (e.g., footprint of an eddy covariance tower or a grid cell in a gridded meteorological driver) is called a *polygon*. Each polygon is subdivided into *patches*, which represent collections of forest gaps within a polygon that share a similar age since last disturbance and same disturbance type (although not necessarily contiguous in space). Patches are further subdivided into *cohorts*, which are collections of individual plants that have similar size and similar functional group. Importantly, because ED‐2.2 incorporates the horizontal heterogeneity of the plant community structure and composition, the model can efficiently incorporate and simulate the dynamics of degraded forests.

Most of the ED‐2.2 modules used in this study have been previously described in Longo, Knox, Medvigy, Levine, Dietze, Kim, et al. (2019). The main changes used in this study include (1) a modified height‐diameter allometry based on the Jucker et al. (2017) approach and locally collected field data that can be used consistently by the initialization and model; (2) an improved allocation to living and structural tissues, which is now based on more recent allometric equations (Chave et al., 2014; Falster et al., 2016) and data sets (Falster et al., 2015); (3) a revised photosynthesis solver, which now accounts for the maximum electron transport ratio and the maximum triose‐phosphate utilization (Lombardozzi et al., 2018; Oleson et al., 2013; von Caemmerer, 2000); and (4) updated values of traits that are used to define trade‐offs in tropical plant functional types in ED‐2.2 (wood density and leaf turnover rate) and update the trade‐off relationships of traits that directly or indirectly influence GPP and light and water use efficiency (specific leaf area and leaf carbon:nitrogen ratio, maximum carboxylation rate, maximum electron transport ratio and maximum triose‐phosphate utilization), using multiple studies and trait databases, including GLOPNET, TRY, and NGEE‐Tropics (Bahar et al., 2017; Baraloto et al., 2010; Chave et al., 2009; Gu et al., 2016; Kattge et al., 2009, 2020, 2011; Norby et al., 2017; Powers & Tiffin, 2010; Santiago & Wright, 2007; I. J. Wright et al., 2004). These changes are described in Text S3. Moreover, we used an approach developed by X. Xu (unpublished) and based on Lloyd et al. (2010) to account for light‐dependent plasticity of three leaf traits (specific leaf area, leaf turnover rate, and carboxylation capacity) and calibrated using existing data (Keenan & Niinemets, 2016; Lloyd et al., 2010; Russo & Kitajima, 2016).

**Figure 2:**
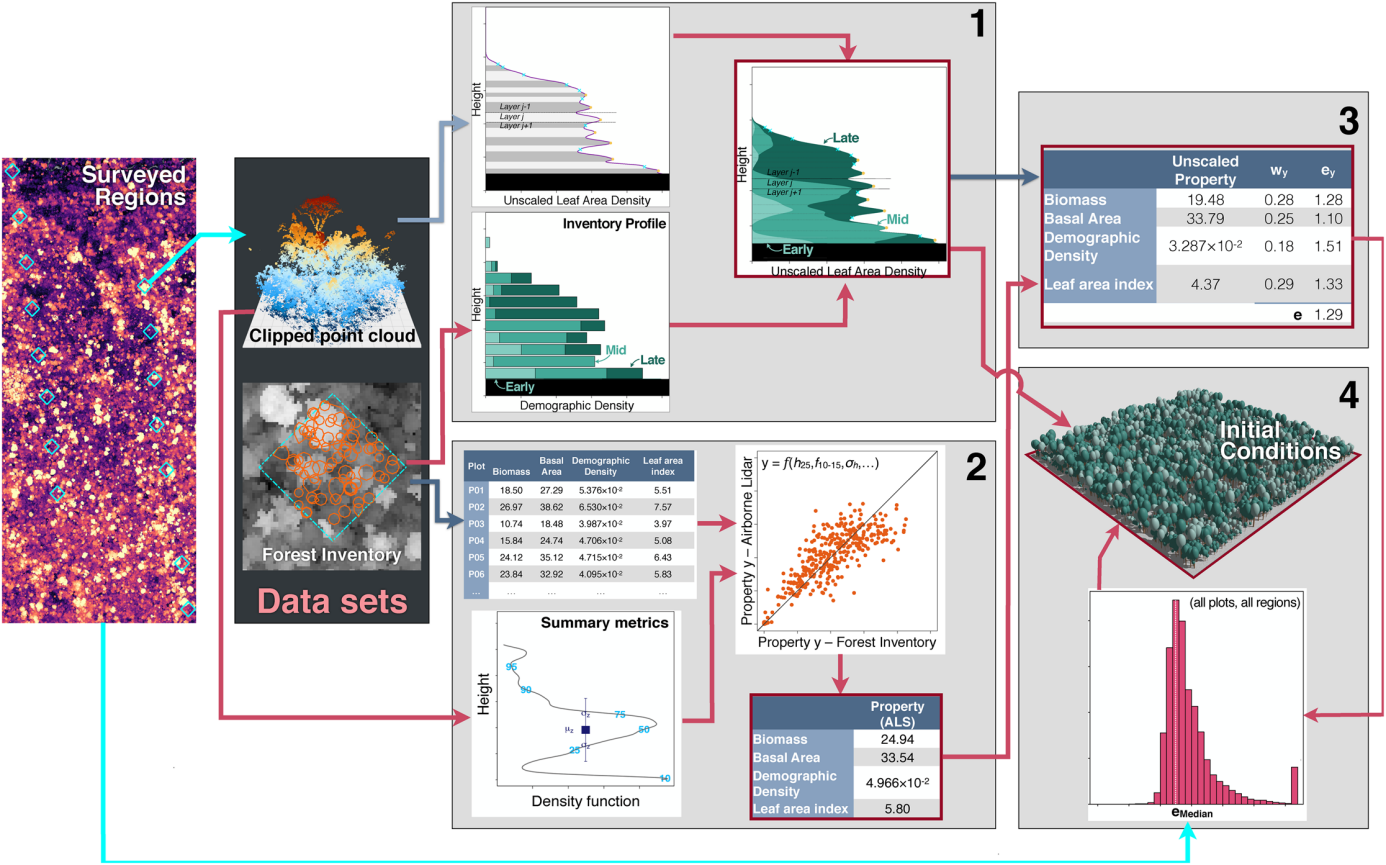
Schematic representation of the method to obtain initial conditions for ED‐2 from airborne lidar. Each light box represents one step in the procedure. The results of each step are highlighted with a red border. Dark blue arrows are stages that require individual‐based allometric equations, and light blue arrows are stages that require a light extinction model.

To obtain initial conditions for ED‐2.2 from airborne lidar, we devised a multistep approach that links airborne lidar data with ecosystem properties (Figure 2). Here we provide a summary of the initialization procedure; the technical details of this approach are described in Text S4. For Step 1, we split all collected point cloud data into 50 × 50 m columns, simulated waveforms from the discrete returns (Blair & Hofton, 1999; Hancock et al., 2019; Popescu et al., 2011) to obtain unscaled leaf area density profiles based on the vertical distribution of returns (e.g., Antonarakis et al., 2014; MacArthur & Horn, 1969; Ni‐Meister et al., 2001; Stark et al., 2012; Tang & Dubayah, 2017), and assigned the relative proportion of each plant functional type provided by one of the 769 training plots that had the most similar vertical structure; the similarity was based on the profile comparison that yielded the smallest Kolmogorov‐Smirnov statistic. The vertical profile was split into cohort layers centered around local maxima or saddle points, using a modified procedure based on function peaks (package RSEIS; Lees, 2017) of the R statistical software (R Core Team, 2019). For Step 2, we used a collection of 817 forest inventory plots (0.16–0.26 ha) that were also surveyed by airborne lidar, which included plots from all study regions as well additional sites available from Sustainable Landscapes Brazil (SLB) and used in a previous study (ancillary SLB sites, Figure 1 Longo et al., 2016); we developed statistical models based on subset selection of regression (Miller, 1984) and heteroskedastic distribution of residuals (Mascaro et al., 2011) to estimate plot‐level properties (aboveground biomass, basal area, stem number density, and leaf area index) from point cloud metrics and field estimates, following the approach by Longo et al. (2016). For Step 3, we sought to obtain a plot‐specific scaling factor to the leaf area density profile that produced the best agreement between the four estimated plot‐level properties from Step 1 and the plot‐level properties obtained by integrating the vertical distribution from Step 2, by minimizing the sum of relative square differences of the four properties. For Step 4, we analyze the scaling factor distribution for all plots for which we could test the approach and define a unique and global scaling factor, based on the median scaling factor, that is used to correct all predicted profiles.

Once we obtained the initial conditions for each 50 × 50 m column, we grouped individual columns based the disturbance history (degradation level) and the study region (Table 1). We used the following broad categories for disturbance history: intact (INT), reduced‐impact logging (RIL), conventional logging (CL*x*, where *x* is the number of logging disturbances), conventional logging and thinning (LTH), logged and burned once (LB1), and burned (BN*x*, where *x* is the number of burns). Importantly, we did not perform any averaging or sampling of the individual columns before providing them to ED‐2.2; instead, we provided all columns to the model, so the initial conditions characterize the observed distribution of forest structures that exist within each group.

### 2.3 Assessment of the Modeling Framework

We evaluated three characteristics to assess the ability of model framework to represent the forest structure heterogeneity caused by degradation and to represent components of the energy, water, and carbon cycle. First, we quantified the ability of the airborne lidar initialization to capture the differences in forest structure caused by degradation. Second, we assessed whether the model can realistically represent fluxes and storage of water, energy, and carbon across different regions. Third, we compared the model sensitivity to degradation‐driven effects on fluxes and storage with independent observations.

To evaluate the airborne lidar initialization, we used a cross‐validation approach in which we replicated the procedure described above (section 2.2) 2,000 times, using a hierarchical bootstrap approach. We first sampled regions (with replacement), to ensure that some regions would be entirely excluded from the replicate, then we sampled plots (also with replacement), to ensure that the replicate had the same number of plots as the original training data set. We then predicted the structure of all plots in the excluded regions, using iterations that did not have any plot in the training data set; to make this number consistent across regions, we used the smallest number of iterations that met this criterion across all regions (*n* = 612). Finally, for each region, we compared the average forest structure from all cross‐validation replicates that excluded the region from the training stage. Because estimates of forest properties have larger uncertainties in smaller plots (Chave et al., 2004; Mauya et al., 2015; Meyer et al., 2013), we only evaluated the method when a disturbance class within a region had at least 20 plots.

To verify the model's ability to realistically represent the regional variability of fluxes and storage, we carried out ED‐2.2 simulations initialized with airborne lidar for the intact forests regions where eddy covariance tower and forest inventory plots colocated with airborne lidar were available (GYF and BTE). Region TAN had two eddy covariance towers, one within the footprint of the burned forests and a second in intact forest (Brando, Silvério, et al., 2019), which allowed us to contrast the model's predicted impacts of degradation on fluxes and biophysical properties with the pair of tower measurements.

### 2.4 Model Configuration and Analyses

Our main focus is to understand the role of degradation‐driven changes in forest structure in altering both the state and the fluxes of energy, water, and carbon, both under typical and extreme climate. To account for regional differences in climate and to sample a broad range of interannual variability, we used time series of meteorological drivers pooled from gridded reanalyses (one set of time series per region). For most meteorological variables required by ED‐2.2 (pressure, temperature, humidity, incoming shortwave and longwave radiation, and winds), we used 0.625° × 0.5°, hourly averages (1980–2016) from the Version 2 of the Modern‐Era Retrospective Analysis for Research and Applications (MERRA‐2; Gelaro et al., 2017). MERRA‐2 precipitation is known to have significant negative biases in the tropics (Beck et al., 2019); therefore, we used the 0.1° × 0.1°, 3‐hourly precipitation rates from the Version 2 of the Multi‐Source Weighted Ensemble Precipitation product (MSWEP‐2; Beck et al., 2019). To ensure that the only difference between simulations in the same study region was the distribution of forest structures, we imposed the same edaphic conditions: free‐drainage soils with 8 m deep and nearly equal fractions of sand (32%), silt (34%), and clay (34%). To avoid confounding effects from postdisturbance mortality and recovery, all simulations were carried out without enabling dynamic vegetation, such that the differences in forest structure would remain the same for the entire time series and all differences between simulations in the same region could be attributable to well‐characterized differences in forest structure. However, disabling dynamic vegetation also precluded us from investigating the effects of climate‐driven changes in the canopy structure on the energy, water, and carbon cycle, thus potentially increasing biases in our estimates of fluxes following extreme events such as droughts.

To investigate the role of degradation on fire risk, we built on the original fire model from ED‐1 (Moorcroft et al., 2001) to determine when fire‐prone conditions would occur in each patch. The flammable area *α*_*F*_ (% yr^−1^) is calculated from the fire disturbance rate *λ*_*F*_ (yr^−1^):

(1) $$\alpha_{F}=100\;\left[1-\exp\left(-\lambda_{F}\;\Delta t\right)\right],$$

(2) $$\lambda_{F}=\left\{\begin{array}{ll} I\;C_{\text{Fuel}}, & \text{if}\;\left[\frac{1}{\left|z_{F}\right|}\int_{z_{F}}^{0}\vartheta (z)dz\right]<\left(1-f\right)\;\vartheta_{\text{Wp}}+f\;\vartheta_{\text{Fc}}\\ 0, & \text{otherwise} \end{array}\right),$$

where Δ*t*=1 yr; *I*=0.5 m^2^ kgC yr^−1^ is a fire intensity parameter; *z*_*F*_=30 cm is the depth of the soil layer used to estimate dryness; *ϑ* (m^3^ m^−3^) is the soil moisture; *ϑ*_Wp_ is the permanent wilting point and *ϑ*_Fc_ is the field capacity, both defined as in Longo, Knox, Medvigy, Levine, Dietze, Kim, et al. (2019); and *f*=0.02 is a phenomenological parameter that defines dry conditions. The values of *I* and *f* were selected based on the results from a previous model evaluation using ED‐2.2 (Longo, Knox, Levine, et al., 2019). Because understory fires are the dominant type of fire in the Amazon (A. Alencar et al., 2006; Morton et al., 2013), we considered fuels to comprise aboveground litter, aboveground coarse woody debris, and aboveground biomass from grasses and seedlings (trees with height <2 m); canopy trees were not considered to be fuels. The fire parameterization, although simple, has been previously demonstrated to capture the general features of fire regime across tropical South America (Longo, Knox, Levine, et al., 2019).

## 3 Results

### 3.1 Evaluation of the Model Initialization and Simulated Seasonal Dynamics

**Figure 3:**
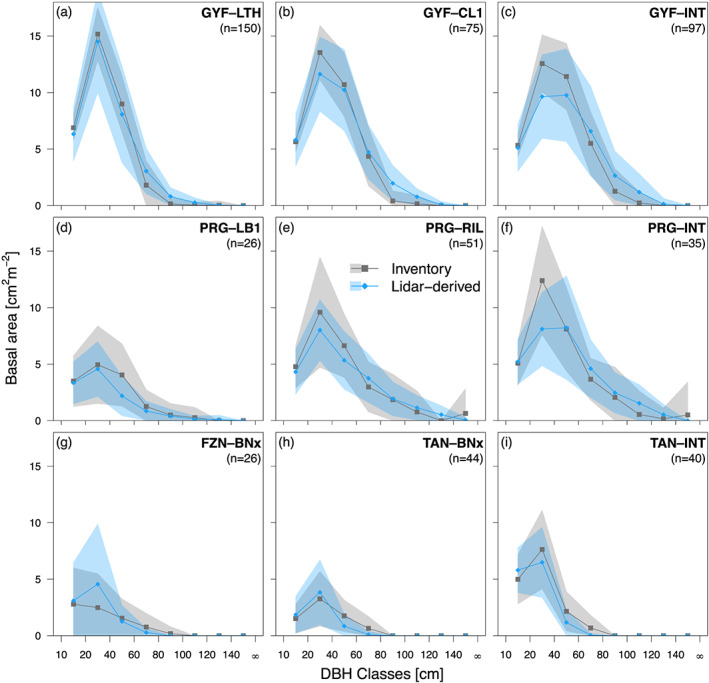
Assessment of basal area distribution as a function of diameter at breast height (DBH) for different study regions and degradation levels. Gray points are obtained from forest inventory plots, and blue points are obtained from the airborne lidar initialization (Figure 2) using a 612‐fold regional cross‐validation (i.e., excluding all plots from region in the calibration stage). Bands around points correspond to the standard deviation either across all plots in the same category (inventory) or across all plots and replicates (lidar). Sites: (a‐c) GYF = Paracou, (d‐f) PRG = Paragominas, (g) FZN = Feliz Natal, (h,i) TAN = Tanguro. Disturbance classes: (g,h) BNx = burned twice or more, (b) CL1 = conventional logging (once), (d) LB1 = logged and burned once, (a) LTH = logged and thinned, (e) RIL = reduced‐impact logging, (c,f,i) INT = intact. Additional comparisons are shown in the supporting information: basal area as functions of plant functional type (Figure S1); leaf area index profiles as functions of height (Figure S2); comparisons for Belterra (BTE‐RIL) (Figure S3).

The ED‐2.2 model initialization approach from airborne lidar (Figure 3) captured the main differences in forest structure and composition, both across study regions and along degradation gradients. To illustrate the initialization, we focus on the basal area distribution obtained from cross‐validation at disturbance histories within study regions that had at least 20 plots (Figure 3). At sites GYF, PRG, and TAN, the airborne lidar initialization predicted the total basal area with absolute biases ranging from 3% (GYF) to 13% (TAN), and root‐mean‐square error of the order of 18–27% (Figures 3c, 3f, and 3i). The largest absolute discrepancies occurred for intermediate‐sized trees (20 ≤DBH < 40 cm) at GYF and PRG, where the airborne lidar initialization underestimated basal area by 2.9 and 4.3 cm^2^ m^−2^, respectively (Figures 3c and 3f). The largest overestimation of airborne lidar was observed among larger trees (60 ≤DBH < 100 cm) in intact forests at GYF (2.4 cm^2^ m^−2^; Figure 3c). The size distribution of most degraded forests were well characterized (Figures 3a, 3b, 3d, 3e, and 3g); the largest deviations from inventory were observed in logged and burned forests in PRG, where airborne lidar underestimated total basal area by 3.0 cm^2^ m^−2^ (Figure 3d). Likewise, the initialization algorithm represented the higher relative abundance of early successional plants in the most degraded sites, and the dominance of midsuccessional and late‐successional plants at intact forests at GYF and PRG (Figure S1), and realistically represented the leaf area distribution across regions and degradation levels (Figure S2).

**Figure 4:**
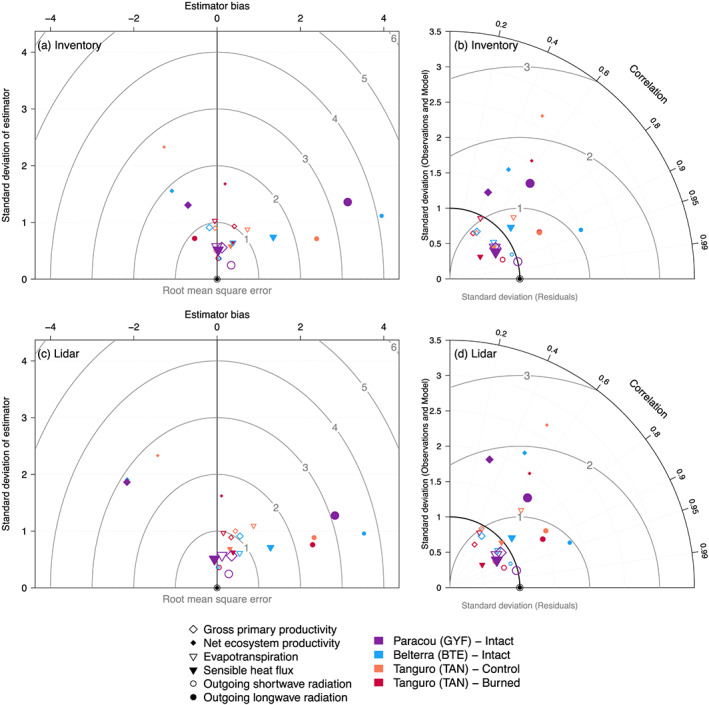
Summary of ED‐2.2 model assessment using eddy covariance towers as benchmarks, using simulations initialized with forest inventory and airborne lidar. (a, c) Bias‐variance diagram and (b, d) Taylor diagram of multiple daily averaged fluxes of carbon, energy, and water for Paracou (GYF), Belterra (BTE), and Tanguro (TAN, control and burned), for simulations initialized with (a, b) forest inventory plots and (c, d) airborne lidar. In the bias‐variance diagram, bias (*x* axis), standard deviation of residuals (*y* axis) and root‐mean‐square error (concentric arcs) are normalized by the standard deviation of observations, as is the standard deviation of models in the Taylor diagram. In both diagrams, ⊙ corresponds to the perfect model prediction. In all plots, we only compare daily averages of days with no measurement gaps. Comparisons of the seasonal cycle for all variables included in the diagrams are available in Figures S4–S9.

ED‐2.2 simulations using forest inventory and airborne lidar as initial conditions were compared with eddy covariance tower estimates of all sites (Figures 4 and S4–S9 and Table S1). GPP generally showed small biases relative to tower estimates (−0.046 to +0.394 kgC m^−2^ yr^−1^) and relatively small errors (less than observed variability) at all sites, regardless of the initial conditions (Figure 4 and Table S1). While the GPP seasonality was correctly represented at GYF, the model did not capture the late wet‐season decrease and early dry‐season increase of GPP at BTE, and it showed a delayed dry‐season decline GPP at TAN compared to tower estimates (Figure S4). Net ecosystem productivity (NEP), on the other hand, showed significant biases, large errors, and relatively small correlation with tower estimates (Figure 4 and Table S1), which were driven by excessive seasonality of heterotrophic respiration (Figure S5). Because the initial carbon stocks in necromass pools are uncertain, and the results on magnitude and seasonality of ecosystem respiration (and consequently NEP) are inconsistent with tower estimates, we will not discuss the simulation results in terms of respiration and NEP.

Water fluxes also showed small biases relative to the observed variability at GYF, TNF, and TAN (Burned), regardless of the initialization (−0.01 to +0.54 mm day^−1^; Figures 4a and 4c and Table S1); biases at TAN (Intact) were larger (0.69–0.82 mm day^−1^). With the exception of TAN (Burned), the correlation between ED‐2.2 and tower was high at daily averages (Figures 4b and 4d and Table S1). At TAN (Burned), the poorer agreement with tower estimates was caused by the model predicting a similar seasonality of water flux at both control and burned forests, whereas towers suggest an increase in water flux during the earlier part of the dry season (Figure S6). ED‐2.2 predictions of sensible heat flux had high correlation with observations at all sites (Figures 4b and 4d and Table S1), although sensible heat flux shows significant biases at BTE and dampened seasonality at GYF and TAN (Burned) (Figures 4a, 4c, and S6 and Table S1). Outgoing shortwave radiation correctly captured the seasonality at the wettest sites, but it did not capture the sharp dry‐season increase at TAN (Figure S8), which may be associated with dry‐season leaf senescence and shedding that was likely underestimated by ED‐2.2. In addition, ED‐2.2 simulations overestimated outgoing longwave radiation at all sites except at TAN (Burned) using inventory initialization (Figure S9). Nonetheless, the seasonality and the intraseasonal variation of outgoing longwave radiation were correctly captured by ED‐2.2, resulting in generally high correlation and small standard deviation of residuals at most sites (Figure 4 and Table S1).

### 3.2 Degradation Effects on Seasonality of Fluxes

**Figure 5:**
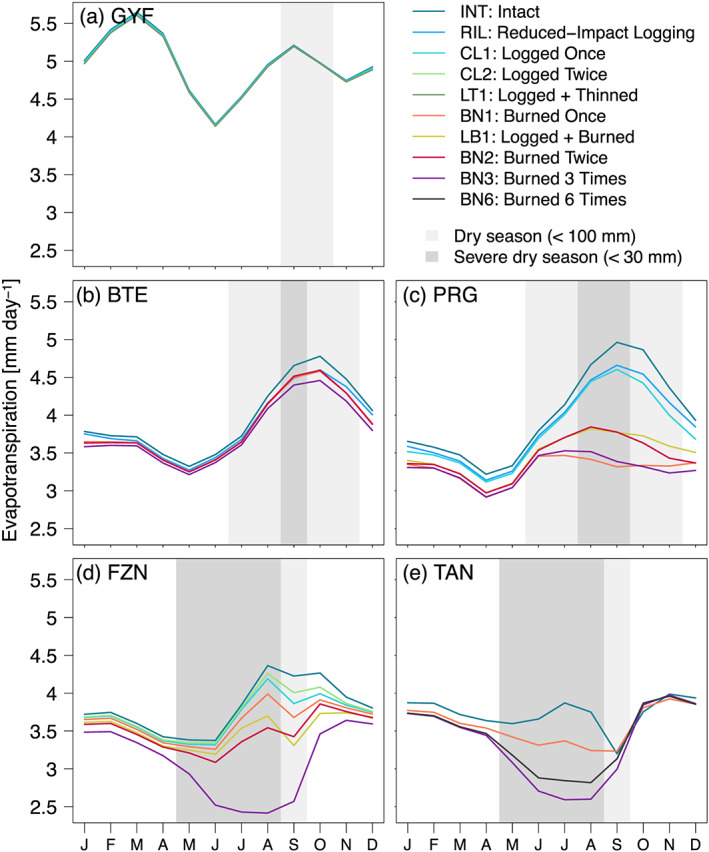
Monthly mean evapotranspiration (ET) as a function of region and degradation. Monthly averages correspond to the 1980–2016 period, simulated by ED‐2.2 for (a) Paracou (GYF), (b) Belterra (BTE), (c) Paragominas (PRG), (d) Feliz Natal (FZN), and (e) Tanguro (TAN), aggregated by degradation history within each region (lines). Gray rectangles in the background correspond to the average dry season.

From ED‐2.2, we found that forest degradation can have substantial impacts on the ecosystem function such as ET or ground temperature in severely or recently degraded forests and in parts of the Amazon with a longer dry season. At GYF, the airborne lidar survey sampled only intact forests and areas that were logged 25 yr prior to the data acquisition: Consequently, the average water vapor flux and ground temperature were nearly indistinguishable across degraded and intact forests (Figures 5a and S10a). At the equatorial sites, degradation effects were small during the wet season but showed marked reduction in ET (2.1–6.7% in BTE and 4.3–31.8% in PRG) and increase in daytime temperature (0.4–0.9°C in BTE and 1.0–6.0°C in PRG) during the dry season, with the largest changes relative to intact forests found at burned areas (Figures 5b, 5c, S10b, and S10c). At the southern (driest) sites, the seasonal changes were even more pronounced: At both FZN and TAN, ET decreased by 21–25% early in the dry season (June) at the most severely burned forests, whereas ET in intact forests peaked in the middle of the dry season (July–August; Figures 5d and 5e). Similarly, burned forests were warmer year‐round than intact forests at the southern sites (FZN and TAN), with minimum warming during the wet season (December–March; 0.5–0.8°C), and maximum warming occurring at the peak of the dry season (July–August; 1.0–6.5°C; Figures S10d and S10e).

**Figure 6:**
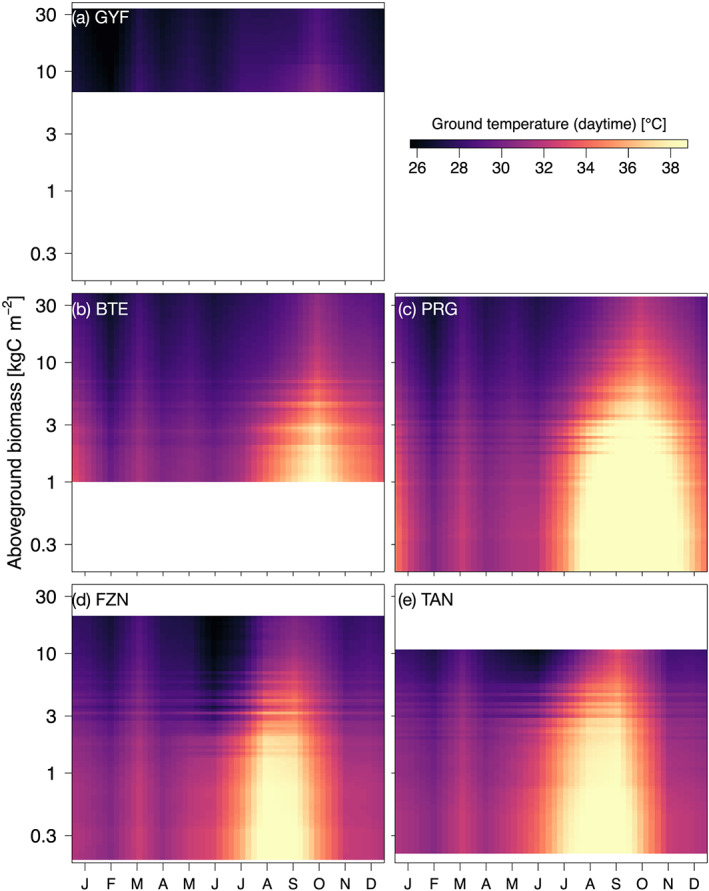
Monthly mean daytime ground temperature as a function of region and local (patch) aboveground biomass. Monthly averages correspond to the 1980–2016 period, simulated by ED‐2.2 for (a) Paracou (GYF), (b) Belterra (BTE), (c) Paragominas (PRG), (d) Feliz Natal (FZN), and (e) Tanguro (TAN), and the *y* axis corresponds to the aboveground biomass for each patch, linearly interpolated for visualization. White areas are outside the range of biomass of each region and thus excluded.

Importantly, the ED‐2.2 results in Figures 5 and S10 emerge from the different distribution of forest structures associated with degradation histories. ED‐2.2 accounts for the diversity of forest structures within each disturbance history by means of patches; each patch represents a different forest structure found within any disturbance regime, and patch area is proportional to the probability of finding such forest structure (Longo, Knox, Medvigy, Levine, Dietze, Kim, et al., 2019). For example, the ground temperature is consistently warmer at the low‐biomass patches, but the differences between the lowest and highest patch temperatures are as low as 1°C at GYF (Figure 6a) and less than 4°C during the wet season even at the southern regions (Figures 6d and 6e). In contrast, differences along biomass gradients exceed 9°C during the dry season at all regions except GYF (Figure 6).

**Figure 7:**
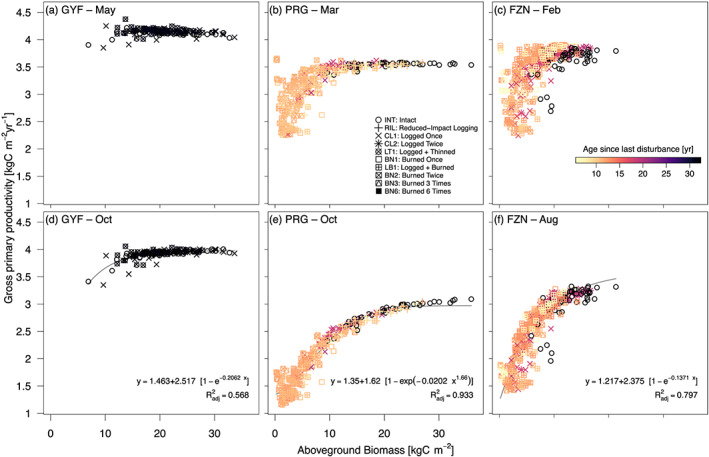
Variability of gross primary productivity (GPP) as a function of local (patch) aboveground biomass (AGB). Scatter plot of AGB (*x* axis) and GPP (*y* axis) at sites (a, d) Paracou (GYF), (b, e) Paragominas (PRG), (c, f) Feliz Natal (FZN), for (a–c) the peak of wet season—May (GYF), March (PRG), and February (FZN)—and (d–f) peak of dry season—October (GYF and PRG) and August (FZN). Each point represents the 1980–2016 average GPP of each patch solved by ED‐2.2; point shapes correspond to the disturbance history, and point colors represent the time between the last disturbance (undetermined for intact forests) and lidar data acquisition. Curves correspond to nonlinear least squares fits of the most parsimonious function, defined from Bayesian information criterion (Schwarz, 1978), between shifted exponential or shifted Weibull functions. Only fits that produced $R_{adj}^{2}>0.5$ were included.

Likewise, when all simulated patches are considered, we observe strong coherence between biomass and GPP across all regions and throughout the year (Figures 7 and S11). However, the effect of local communities on GPP is seasonal: Differences in typical GPP between low‐biomass and high‐biomass patches do not exceed 1.1 kgC m^−2^ yr^−1^ during the wettest months (Figures 7a–7c), whereas the range of GPP reaches 0.7 kgC m^−2^ yr^−1^ at the short dry season at GYF and exceeds 2.0 kgC m^−2^ yr^−1^ during the dry season at the most degraded and driest it does not represent many msites (Figures 7e and 7f). Similar effects were observed in ET, where differences along biomass are the strongest during the dry season (Figure S12).

### 3.3 Degradation Impacts on Forest Flammability

**Figure 8:**
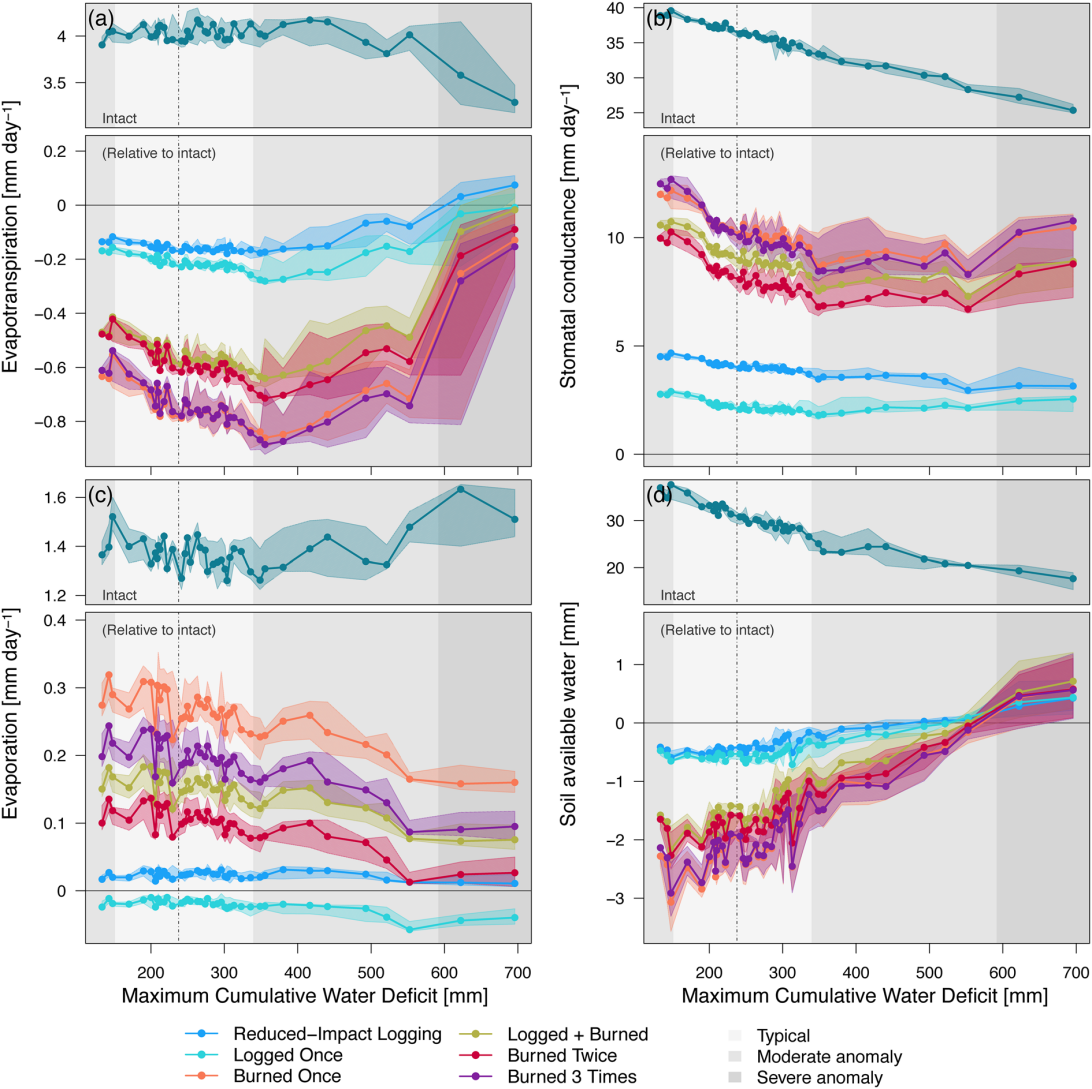
Response of the water cycle components across a forest degradation gradient and drought severity in Paragominas (PRG). Selected components: (a) total water vapor flux, (b) stomatal conductance, averaged by leaf area, (c) evaporation, and (d) soil available water (i.e., in excess of permanent wilting point) of the top 30 cm. Points correspond to the median value of 12 month running averages, aggregated into 40 quantiles along the range of maximum cumulative water deficit (MCWD). Bands around the points correspond to the 95% range within each MCWD bin. Top panels are the absolute value for intact forests, and bottom panels are the absolute difference between degraded and intact forests. Background shades denote the MCWD anomaly: light gray = 68% range around the median (dot‐dash vertical line); intermediate gray = 95% range; dark gray = anomalies exceeding the 95% range.

The impact of forest degradation on ecosystem functioning showed important year‐to‐year variability, and differences between intact and degraded forests were generally larger during typical years than during extreme droughts. For this section, we calculate the monthly water deficit based on the difference between potential ET (calculated following Priestley & Taylor, 1972) and rainfall, relate the 12 month running averages of multiple response variables with the maximum cumulative water deficit over the previous 12 months, and define drought length as the number of consecutive months in water deficit exceeds 20 mm. Using region PRG as an example, as the region has the broadest range of recent disturbances and maximum cumulative water deficit, we found that, during typical rainfall periods, ET in logged forests and burned forests were 3–6% and 11–22% lower than intact forests, respectively (Figure 8a); this difference was significantly reduced or even reversed during severe droughts, when ET of degraded forests were up to 4% higher than in intact forests (Figure 8a). Degraded forests have a lower proportion of shade‐tolerant, late‐successional trees, and typical stomatal conductance is higher by 19–34% in burned forests and by 5–13% in logged forests (Figure 8b). This result indicates that the reduced typical ET results from degraded forests having lower leaf area index relative to intact forests, as local leaf area index is related to local aboveground biomass (Figure S13). In addition, extreme droughts did not substantially reduce the differences in stomatal conductance between degraded and intact forests (Figure 8b). While ET was generally lower in degraded forests, total evaporation (from ground and canopy intercepted water) was higher in most degraded forests, with burned forests experiencing 3–26% more evaporation in typical years and 0–14% during severe droughts (Figure 8c). The combination of higher evaporation and relatively shorter canopy (shallower roots) in degraded forests were typically translated into slightly drier near‐surface soils (Figure 8d): During typical years, soil water availability at the top 30 cm layers was 1.2–12% lower in burned forests than intact forests, whereas the differences were more modest in logged forests (0.2–3%) and even reversed during extreme droughts (Figure 8d). Carbon and energy fluxes showed similar behavior. GPP in intact forests steadily decreased with increased drought severity, and the depletion of productivity caused by degradation is most marked during typical years but is reduced during severe droughts (Figure S14a). While ground temperature is always higher in degraded forests (Figure S14b), differences in sensible heat fluxes and outgoing longwave radiation also diminish during extreme drought conditions (Figures S14c and S14cd).

**Figure 9:**
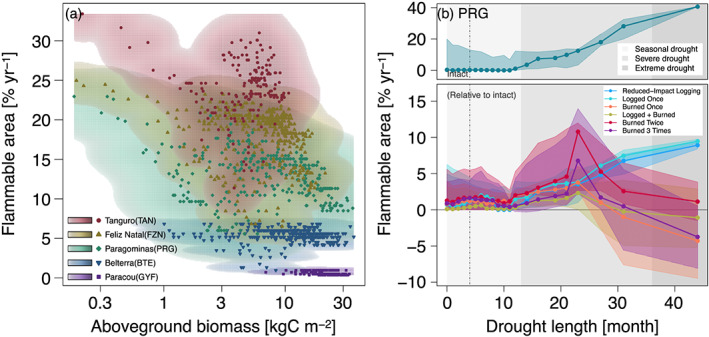
Average flammability as functions of degradation and climate variability. (a) Scatter plot shows the average flammable area (1980–2016) for each simulated patch across all regions, as a function of canopy height. Density cloud (background color) was produced through a bidimensional kernel density estimator; points are the averages used to generate each density cloud. Color ramps (logarithmic) range from 0.1–100% of the maximum computed scale. (b) Flammable area at region PRG, as a function of degradation history and drought length (number of consecutive months with water deficit in excess of 20 mm). Points correspond to the median value of 12 month running averages, aggregated into quantiles along the drought length. Bands around the points correspond to the 95% range within each drought length bin. Top panels are the absolute value for intact forests, and bottom panels are the absolute difference between degraded and intact forests. Background shades denote drought length classes used in the text: seasonal (light gray, less than 12 months), severe (intermediate gray, 12–36 months), and extreme (dark gray; more than 36 months). Flammability response to degradation and drought duration for other regions are shown in Figure S15.

Degraded forests show drier near‐surface soils (Figure 8d) and warmer surface temperatures (Figure S14) than intact forests for most years, yet the interannual variability of climate also modulates the differences in water, carbon, and energy cycles between degraded and intact forests (Figures 8 and S14). Therefore, both degradation and climate may influence the flammability of forests. The average flammable area predicted by ED‐2.2 (section 2.4) shows large variation across regions, ranging from nearly 0 at GYF forests (the wettest region) to over 25% yr^−1^ at some of the forests in TAN (the driest region) (Figure 9a). Within each region (i.e., under the same prescribed climate), the model generally predicted higher flammability for the shortest forests (<10 m), although predictions also indicate large within‐region variability of flammable area for forests with intermediate canopy height (10–25 m) (Figure 9a). For most forests, flammable conditions were predicted mostly during moderate or severe droughts, regardless of the degradation history, as exemplified by region PRG (Figure 9b). While the time series of flammable area were synchronized across degradation types, ED‐2.2 predictions of flammable area were generally higher for burned forests than intact or lightly logged forests (Figures 9b and S15). The one exception was the driest region (TAN), where forests that burned multiple times experienced lower flammability than intact forests (Figure S15d); at TAN, even intact forests were relatively short (Figure 9a), which caused ED‐2.2 to predict limited access to deeper soils and increased desiccation.

## 4 Discussion

### 4.1 Initialization of Forest Structure From Remote Sensing

Our method to derive the vertical structure of the canopy from high‐resolution airborne lidar successfully characterized the diversity of forest structures of the Amazon, captured differences in forest structure variability along a precipitation gradient, and described the within‐region variability in forest structure caused by forest degradation (Figures 3, S2, and S3). Previous studies have used forest structure derived from remote sensing data to initialize vegetation demography models in tropical forests (e.g., Antonarakis et al., 2011; Hurtt et al., 2004; Rödig et al., 2018). However, these studies often assume a relationship between forest structure and canopy height with stand age. While this assumption has been successfully applied to intact and second‐growth tropical forests (Antonarakis et al., 2011; Hurtt et al., 2004), the association between forest structure and succession is unlikely to be preserved in degraded forests. For example, understory fires proportionally kill more smaller trees than large trees (Brando et al., 2012; Silva et al., 2018; Uhl & Kauffman, 1990), and selective logging creates complex mosaics of forest structure, with substantial losses of large trees from harvesting and extensive damage to smaller trees in skid trails (Feldpausch et al., 2005). In contrast, our approach accounts for the entire vertical profile at local (50 m) scale, similarly to Antonarakis et al. (2014), which does not require any assumption on the successional stage of the forest. Importantly, our approach requires only the vertical distribution of returns and could be adapted to large‐footprint, airborne or spaceborne lidar data, including the NASA's Global Ecosystem Dynamics Investigation (GEDI; Hancock et al., 2019).

We demonstrated that the initialization from airborne lidar profiles captures most of the variability across and within regions, yet it has important assumptions and limitations. First, our approach relies on allometric equations to determine both the diameter at breast height (DBH) and the individual leaf area (*L*_*i*_, Text S4.3), with the implicit assumption, that the contribution of branches, twigs, and stems to the lidar return signal is negligible. In reality, allometric equations have either large uncertainties (DBH) or limited number of samples (Figure S16). Previous studies using destructive sampling and terrestrial laser scanning suggest that wood area index may constitute 7–15% of the plant area index (Olivas et al., 2013; Schneider et al., 2019). The use of allometric equations that account for regional variation (e.g.,  Feldpausch et al., 2011, 2012) and the expansion of open‐source databases, such as the Biomass And Allometry Database (BAAD;  Falster et al., 2015) used in our study, could further improve the characterization of the vertical structure. In addition, the increased availability of terrestrial laser scanning (TLS) and high‐resolution, low‐altitude unmanned aerial vehicle lidar could substantially increase the data availability and thus improve the overall quality of allometric equations and constrain the relative contribution of woody tissues to the total plant area (Calders et al., 2015; Schneider et al., 2019; Stovall et al., 2018). Alternatively, techniques that extract individual tree crowns from lidar point clouds readily provide highly accurate local stem density and local size‐frequency distributions (e.g., tree height or crown size; Ferraz et al., 2016, 2020). These distributions can be used to attribute DBH to individuals and generate initial conditions akin to forest inventory to the ED‐2.2 model, and data‐model fusion techniques that leverage the growing availability of data could reduce uncertainties on many model parameters, including allometry (F. J. Fischer et al., 2019). Finally, ED‐2.2 overestimated the seasonality of GPP and ET at the driest region (TAN) (Figures S4 and S6). This result suggests that simulated rooting depth for TAN was underestimated in the model. Rooting profiles in tropical forests remain largely uncertain: Some site studies have sought to relate individual tree size with rooting depth using isotopic measurements (e.g., Brum et al., 2019; Stahl et al., 2013), whereas regional studies that provide spatial distribution of rooting depth still show important discrepancies in the tropics (e.g.,  Fan et al., 2017; Yang et al., 2016). Constraining the belowground allocation of tropical ecosystems should be a priority in future studies.

In our study we inferred the functional diversity from forest structure obtained from existing forest inventory plots. The functional group attribution captured the general characteristics of functional composition along degradation gradients (Figure  S1), including the more frequent occurrence of early successional individuals in degraded forests, consistent with field‐based studies (Both et al., 2019); nonetheless, uncertainties in functional attribution from field measurements are high. The increased availability of coordinated airborne laser scanning (ALS) and airborne imaging spectroscopy (AIS) data in midlatitudes has lead to opportunities to link structural variability with functional diversity (e.g., Antonarakis et al., 2014; Schneider et al., 2017), and previous studies have successfully integrated ALS and AIS data to attribute functional groups in the ED‐2 model (e.g.,  Antonarakis et al., 2014; Bogan et al., 2019). Overlapping ALS and AIS data over tropical forests are becoming increasingly common (Asner et al., 2014; de Almeida et al., 2019; Laybros et al., 2019) and could provide new opportunities to reduce uncertainties in functional attribution in future studies. Likewise, ongoing and upcoming spaceborne missions at the International Space Station such as GEDI (Hancock et al., 2019) and the Hyperspectral Imaging Suite (HISUI;  Matsunaga et al., 2017) will allow for large‐scale characterization of structure and function of ecosystems at global scale (Schimel et al., 2019; Stavros et al., 2017).

### 4.2 Degradation Impacts on Ecosystem Functioning

In addition to carbon losses and structural changes, degradation has substantial impacts on energy and water cycles in Amazonian forests, especially in severely degraded forests with marked dry season. According to the ED‐2.2 simulations, ground temperature of logged forests ranged from nearly identical to intact forests (low‐impact logging or old logging disturbances) to 0.7°C warmer (recently logged forests), whereas severely burned forests experienced daytime near‐surface temperatures increases of as much as 4°C (Figure S10), and differences between the lowest and highest biomass patches exceeded 9°C (Figure 6). Observed differences in understory temperatures show large variability, but they generally agree with the ED‐2.2 results. For example, results of temperature differences between logged and intact areas in the wet forests of Sabah, Malaysia, ranged from negligible to 1.2°C for average maximum temperature (Jucker et al., 2018; Senior et al., 2018). The predicted warmer daytime understory temperatures at recurrently burned forests also yielded drier near‐surface conditions: Daytime ground vapor pressure deficit was on average 15–25 hPa greater than in intact forests (equivalent to 5–15% reduction in relative humidity), which is within the range observed after the most damaging experimental fire at TAN in 2007 (Brando et al., 2014) and similar to differences in understory relative humidity reported in the dry season between open‐canopy seasonally flooded forests and closed‐canopy upland forests in the central Amazon (de Resende et al., 2014). Because temperatures are higher in degraded forests, the simulated changes in energy and water cycle caused by degradation also point to a reduction of entropy production in degraded forests, which is consistent with the results across pastures and intact forests across the Amazon (Holdaway et al., 2010).

ED‐2.2 showed various degrees of agreement with the few existing observational studies comparing changes in ET due to degradation. ET response to reduced‐impact logging was minor (−1.9% reduction relative to intact in BTE), consistent with eddy covariance tower estimates in a logging experiment in the same region (−3.7% reduction after accounting for site differences and interannual variability;  Miller et al., 2011). The model results for the experimental fire at TAN, however, suggested similar wet‐season ET between burned and intact forests (ΔET = ET_Brn_−ET_Int_=0.002 mm day^−1^), with stronger depletion of ET in burned forests during the dry season (ΔET = −0.31 mm day^−1^) (Figures 5 and S6). In contrast, Brando, Silvério, et al. (2019) found higher ET in burned forests over a period of 4 yr, albeit ΔET also showed significant interannual variability. A few other studies suggest that the significant decline in dry‐season ET in burned forests may be expected in some areas: For example, Hirano et al. (2015) found that ET of drained and burned peatlands with second‐growth vegetation in Central Kalimantan (Indonesia) was 0.43 mm day^−1^ lower than drained forests; Quesada et al. (2004) inferred ET changes from soil water budget in savannas and found significant reductions following fires in a savanna site in central Brazil. The advent of high‐resolution remote sensing products that quantify energy, water, and carbon fluxes, such as the ECOsystem Spaceborne Thermal Radiometer Experiment on Space Station (ECOSTRESS) and the Orbiting Carbon Observatory 3 (OCO‐3), will provide new opportunities to quantify the role of tropical forest degradation on ecosystem functioning at regional scale (Schimel et al., 2019), as well as to provide new benchmark data for ecosystem models.

Our model results indicate that severe degradation substantially alters the magnitude and seasonality of energy, water, and carbon fluxes (Figures 5, 6, 7 and S10–S12). In our study, we disabled the vegetation dynamics in ED‐2.2 to ensure that predicted differences in ecosystem functioning could be unequivocally attributed to structural diversity, but the differences in ecosystem functioning between degraded and intact forests may diminish over time as the forest recovers from previous disturbance. This pathway is consistent with the relatively small differences in ET and surface temperature (Figures 5 and 6) observed at logged forests at GYF (25 yr since last disturbance) and burned forests at BTE (15 yr since last disturbance). However, the recovery trajectory is one out of multiple possible pathways: Degraded forests may be more prone to subsequent disturbances (Hérault & Piponiot, 2018; Silvério et al., 2019); the recovery dynamics can be long or not attainable if multiple stable states exist or if succession is arrested (Ghazoul & Chazdon, 2017; Mesquita et al., 2015), potentially prolonging the impacts of forest degradation on energy and water cycles; and feedbacks on precipitation caused by degradation could affect the spatial distribution of rainfall similarly to the effect observed with deforestation (Spracklen et al., 2018), although to our knowledge this impact has not yet been quantified for degraded forests.

In this study, we focused on the effects of forest structure on ecosystem function, and thus we used idealized, homogeneous soil with intermediate hydraulic characteristics in all simulations. In reality, soils across the Amazon are highly heterogeneous and directly affect forest structure across the biome (Quesada et al., 2012). Likewise, soil depth and texture and variability in local topography also modulate the effects of tropical forest degradation on microclimate (Jucker et al., 2018). A previous study using ED‐2.2 found that ET in central Amazonia could decrease by 12–16% under scenarios of recurrent yearlong droughts (40% reduction in rainfall), but the severity of the decrease varied by 7% under the same climate scenarios but different soil hydraulic properties (Longo et al., 2018). These results suggest that degraded forests in clay‐rich, compact soils and deeper water table could amplify reductions in ET and GPP during the dry season, while degradation effects on energy, water, and carbon cycle would likely be dampened in regions where the water table is near the surface for most of the year or soils with higher water storage capacity.

### 4.3 Interactions Between Forest Degradation and Climate Variability

The predicted reductions in ET in the most degraded areas during the dry season suggest that land use change impacts on the water cycle may be more widespread and pervasive than indicated by earlier studies. Previous model‐based studies showed that biome‐wide deforestation could cause ET to decrease by 25–40% relative to intact forests in the Amazon during the dry season (e.g., von Randow et al., 2004; Zemp et al., 2017). These reductions are comparable to the ET reductions predicted by ED‐2.2 at the most degraded forests (21–32%, Figure 5). Because tropical forest degradation affects an area comparable to deforestation in the Amazon (Tyukavina et al., 2017), it may further reduce the strength of the Amazon water vapor source to the atmosphere. In our study, we focused on understanding how climate and structure variability impacts the water and energy fluxes, but degradation‐driven changes in these fluxes are likely to feed back into the atmosphere. For example, changes in ET and sensible heat flux associated with deforestation are known to either redistribute or reduce total rainfall in tropical forests (Spracklen et al., 2018, and references therein), and a substantial fraction of South American precipitation water comes from ET from Amazonian forests (van der Ent et al., 2010). Recent estimates of ET for the Amazon Basin from the Gravity Recovery and Climate Experiment (GRACE) suggest that the basin‐wide ET (including intact forests) has decreased by 1.7% between 2002 and 2015 (Swann & Koven, 2017). In addition, several studies suggest that the dry season in the Amazon is becoming longer (Fu et al., 2013; Sena et al., 2018), and land use change is one of the main drivers of the drying trend (Barkhordarian et al., 2018). The role of forest degradation on ongoing and future changes in climate across the Amazon remains uncertain and deserves further investigation, potentially with coupled biosphere‐atmosphere models that represent heterogeneity in forest structure and functioning (Knox et al., 2015; Swann et al., 2015; Wu et al., 2017). Likewise, we could not account for cascading effects of climate on the energy, water, and carbon cycle in this study because we disabled dynamic vegetation. However, severe droughts are known to increase mortality rates and canopy turnover in tropical forests (Feldpausch et al., 2016; Leitold et al., 2018; Phillips et al., 2010); such disturbances may increase gap fraction and thus reduce GPP and ET in the years immediately following the drought. Future studies that include dynamic vegetation can provide further insights on the resilience and resistance of degraded and intact forests to climate extreme.

Our results show that structural changes resulting from forest degradation make the forest surface drier and warmer (Figures 5, 6, 7, 8 and S10). Drier and warmer conditions near the surface increase flammability (Brando, Paolucci, et al., 2019, and references therein), and it has been long suggested that forest degradation and canopy opening make forests more likely to burn (e.g., A. A. C. Alencar et al., 2015; Cochrane et al., 1999; Ray et al., 2005; Uhl & Buschbacher, 1985). The ED‐2.2 simulations indeed predicted higher flammability in degraded (more open‐canopy) forests on any given year (Figures 9 and S15). However, our results also suggest that climate strongly drives the variability of flammable area across most of our study regions (Figures 9b and S15), which is consistent with the significant increases in forest fires in the Amazon during extreme drought years (Aragão et al., 2018; Morton et al., 2013). Moreover, our results indicate that differences in flammable area between intact and degraded forests are reduced or even reversed during extreme droughts, which indicates that under extreme conditions, the level of degradation is less critical to create flammable conditions. This effect was predicted for most years at TAN, which typically experiences severe and longer dry seasons compared to the other study regions (Figure S15).

Previous studies suggest that parts of the eastern Amazon could become drier by the end of the century and experience more extreme events, including droughts (Duffy et al., 2015; IPCC, 2014), and thus potentially more susceptible to future fires (Brando et al., 2020; De Faria et al., 2017). However, how tropical forest flammability will respond in the long‐term to ongoing changes in climate and land use is still uncertain, and recent studies have shown that either climate (Le Page et al., 2017) or land use (Fonseca et al., 2019) could be dominant on predicted shifts in fire regime. Importantly, while our analysis focused on flammability, and ED‐2.2 fire model captures the general patterns of fire disturbance across the Amazon (Longo, Knox, Levine, et al., 2019), it does not represent many mechanisms and processes that are critical to describe fire dynamics in tropical forests, such as anthropogenic ignitions, diurnal cycle of fire intensity, and fire termination; therefore, we could not quantify the effects of fire on further forest degradation. The use of process‐based fire disturbance models within the ED‐2.2 (e.g.,  Le Page et al., 2015; Thonicke et al., 2010) framework could contribute to further improve our understanding of interactions between forest degradation, climate, and flammability across the Amazon.

## 5 Conclusion

Our study showed that tropical forest degradation can markedly modify the ecosystem functioning in the Amazon, with substantial reductions in ET and GPP and increase in surface temperature (Figures 5, 6, 7, 8). Within the regions included in our study, the effects of degradation on energy, water, and carbon cycles were the strongest in the eastern and southern Amazon, where the dry season is more pronounced. Notably, in areas where severe forest degradation resulted in substantial changes in forest structure, reductions in dry‐season ET are similar to those found in deforested areas (Figure 5;  von Randow et al., 2004). The area of the Amazon forest impacted by degradation is comparable to the deforested area (Asner et al., 2005; Morton et al., 2013; Souza Jr. et al., 2013; Tyukavina et al., 2017), and thus degradation‐driven changes in water, energy, and carbon cycles are potentially important. However, the extent to which degradation affects the biophysical and biogeochemical cycles at regional scale ultimately depends on (1) annual degradation rates; (2) recovery time of degraded forests; and (3) the likelihood that degraded forests are cleared. For example, Brando, Silvério, et al. (2019) found that ET in burned forests was indistinguishable from intact forests 7 yr after the last fire. While their result suggests fast recovery of degraded forests, the impacts of degradation on ET can still be regionally relevant if degradation rates are sufficiently high to maintain low average age since last disturbance in degraded forests. Moreover, we found that the impacts of tropical forest degradation on energy, water, and carbon cycles and on flammability are more pronounced during typical years than during extreme droughts (when all forests become flammable), which highlights the complex interactions between climate and forest structure. To understand and reduce uncertainties of climate‐structure interactions, it would be valuable to leverage the recent advances in remote sensing of forest structure, including the recently launched GEDI mission (Hancock et al., 2019) and terrestrial biosphere models that can represent complex and heterogeneous ecosystems (Fisher et al., 2018). Our study, while focusing on airborne lidar data, has demonstrated the opportunities to integrate remote sensing and terrestrial biosphere models even in regions with complex forest structure such as degraded forests.

## Supporting information



Supporting Information S1

## Data Availability

Airborne lidar and forest inventory data were obtained from Sustainable Landscapes Brazil (2019), dos‐Santos et al. (2019) (Brazil), and Paracou Portal (2016) (French Guiana). MERRA‐2 reanalyses are available from GMAO (2015a, 2015b, 2015c, 2015d), and MSWEP‐2.2 data were downloaded from https://www.gloh2o.org. The ED‐2.2 model used in this study is available at Longo, Knox, Medvigy, Levine, Dietze, Swann, et al. (2019), and the scripts and ED‐2.2 output are permanently stored at Longo et al. (2020). Trait data are available at the TRY initiative on plant traits (https://www.try-db.org), request 2751; at Gu et al. (2016); or as supporting information from the cited references (Bahar et al., 2017; Santiago & Wright, 2007; I. J. Wright et al., 2004).
