# Supplementary material for: Impacts of Degradation on Water, Energy, and Carbon Cycling of the Amazon Tropical Forests
Source: J Geophys Res Biogeosci. 2020 Aug 20;125(8):e2020JG005677. doi: 10.1029/2020JG005677 (PMC7507752; doi:10.1029/2020JG005677)
Supplement: Supplementary file 1 — Supporting Information S1 [file JGRG-125-e2020JG005677-s001.pdf]

Supporting Information for "Impacts of Degradation on Water, Energy, and Carbon Cycling of the Amazon Tropical Forests"

Marcos Longo[1],Sassan Saatchi[2,3],Michael Keller[2,4,5],Kevin Bowman[2],António Ferraz[2,3],Paul R. Moorcroft[6],Douglas C Morton[7], Damien Bonal[8],Paulo Brando[9,10,11],Benoît Burban[12],Géraldine Derroire[13],Maiza N dos-Santos[5],Victoria Meyer[2],Scott Saleska[14],Susan Trumbore[15],Grégoire Vincent[16]

[1]NASA Postdoctoral Program Fellow, Jet Propulsion Laboratory, California Institute of Technology, Pasadena CA, United States

[2]Jet Propulsion Laboratory, California Institute of Technology, Pasadena, CA, United States

[3]Institute of Environment and Sustainability, University of California, Los Angeles, CA, United States

[4]International Institute of Tropical Forestry, USDA Forest Service, Rio Piedras, Puerto Rico

[5]Embrapa Informática Agropecuária, Campinas, SP, Brazil

[6]Department of Organismic and Evolutionary Biology, Harvard University, Cambridge, MA, United States

[7]NASA Goddard Space Flight Center, Greenbelt, MD, United States

[8]Université de Lorraine, INRAE, AgroParisTech, UMR Silva, F-54000 Nancy, France

[9]Department of Earth System Science, University of California, Irvine, CA, United States

[10]Woods Hole Research Center, Woods Hole, MA, United States

[11]Instituto de Pesquisa Ambiental da Amazônia, Brasília, DF, Brazil

[12]INRAE, UMR 0745 EcoFoG, Campus Agronomique, Kourou 97379, France

[13]CIRAD, UMR EcoFoG (AgroParisTech, CNRS, INRAE, Univ. Antilles, Univ. Guyane), Kourou 97379, France

[14]University of Arizona, Tucson, AZ, United States

[15]Max-Planck-Institut für Biochemie, Jena, Germany

[16]AMAP, Univ Montpellier, IRD, CIRAD, CNRS, INRAE, Montpellier, 34000 France

Contents to this file

1. Text S1 to S4
2. Figures S1 to S21
3. Table S1 to S4

Additional Supporting Information (Files uploaded separately)

1. Captions for Dataset S1

Introduction

This supporting material provides additional information on the study sites, methodology, and results in the main text. Text S1 provides information on the disturbance history of the selected study regions. Text S2 contains additional information on the airborne lidar and forest inventory plot data used in this study. Text S3 summarizes changes in the ED-2.2 model to improve the representation of forest structure and ecosystem functioning. Text S4 describes in detail the steps needed to obtain ED-2.2 initial conditions from airborne lidar.

Figures S1, S2 and S3 provide additional evaluation of the airborne lidar initialization, specifically the distribution of functional groups, the vertical leaf area index profile, and the evaluation of plots affected by reduced-impact logging in region BTE. Figures S4-

S9 complement the ED-2.2 model evaluation against eddy covariance towers, comparing fortnightly averages for multiple energy, water, and carbon cycle variables. Figure S10 shows the differences in the average seasonal cycle of daytime ground temperature for all the regions simulated by ED-2.2, as functions of the degradation history. Figure S11 shows the ED-2.2 predictions of average seasonal cycle of gross primary productivity as functions of local (patch) aboveground biomass for all focus regions. Figure S12 shows the distribution of evapotranspiration as function of local (patch) biomass and age since last disturbance, during the wet and dry seasons, for three selected regions across the precipitation gradient. Figure S13 shows the local (patch) distribution of leaf area index as a function of aboveground biomass for all the focus regions. Figure S14 shows the drought severity response of intact and degraded forests in region PRG, for multiple carbon and energy variables. Figure S15 complements Figure 9 shows how forest flammability varies as a function of drought length across degradation gradients at additional regions. Figure S16 is part of Text S2 and shows the fitted allometric models relating height, diameter at breast height, and individual leaf area, which are used by both the model initialization and model simulations. Figures S17 and S18 are also part of Text S2 and show multiple trait relationships derived from multiple data sets and implemented in the ED-2.2 model. Figure S19 is part of Text S3 and shows an example of how the vertical distribution of lidar returns is processed to obtain cohorts that are provided to the ED-2.2 model. Figure S19 is also part of Text S3 and shows the results of cross-validation of airborne lidar initialization using aggregated forest inventory plot metrics as benchmarks. Figure S21 is also part of Text S3 and summarizes the distribution of scaling factors to adjust the non-dimensional leaf area density profiles.

Table S1 shows a selection of metrics to assess the ED-2.2 model performance against multiple energy, water, and carbon cycle variables obtained from the eddy covariance towers. Table S2 is part of Text S1 and provides additional information of data used for the five focus regions and the ancillary regions. Table S3 is part of Text S2 and provides detailed information on ED-2.2 model settings. Table S4 is part of Text S3 and lists multiple goodness-of-fit statistics for the fitted models that relate airborne lidar metrics and aggregated, area-based forest properties.

S1. Disturbance history of the study regions

Here we briefly describe the disturbance history for each region, which in some cases comprised multiple sites. The disturbance history of most of the sites in Brazil has been previously described in Longo et al. (2016), and detailed information on the disturbance history in GYF can be found in Gourlet-Fleury, Guehl, and Laroussinie (2004). A summary of data collected in each site is shown in Table S2.

1. *Paracou, French Guiana (GYF).* This is a research field station was established in 1983 to study the dynamics of logged forests under a variety of silvicultural treatments (Gourlet-Fleury et al., 2004). Since then the a broader range of studies on functional ecology and biodiversity have been established at the research station, including the areas of intact forests.

- *Logging experiment (PRC).* Twelve forest inventory plots (6.25 ha) were established to monitor the dynamics of logged forests under different logging treatments. Following the first survey (1984), plots were grouped into three categories according to their forest structure, and plots within categories were randomly assigned to one of the four treatments which were carried out between 1986 and 1988: (T1) conventional selective

logging of commercial species ($10\,\text{trees}\,\text{ha}^{-1}$; diameter at breast height DBH $\geq 50\,\text{cm}$); (T2) conventional logging as in T1, followed by canopy thinning by poison-girdling of non-commercial species ($30\,\text{trees}\,\text{ha}^{-1}$, DBH $\geq 40\,\text{cm}$); (T3) similar to T2, but the additional logging of non-commercial species ($40 \leq \text{DBH} < 50\,\text{cm}$) for fuelwood; (T4) no treatment. No further logging treatment has been carried out since then. Additional information available in Gourlet-Fleury, Ferry, Molino, Petronelli, and Schmitt (2004).

- *Guyaflux site (GFE).* This site is the footprint of the eddy covariance tower at Paracou, which was installed in 2003, along with 10 plots ($0.49 - -1\,\text{ha}$) within the tower footprint. The tower footprint covers mostly large hills with a small valley with a creek and a sandy plateau. See Bonal et al. (2008) for further details about the site.

2. *Belterra, Brazil (BTE).* Throughout the 20th and early 21st centuries, this region experienced multiple economic growth and stagnation cycles, which led to a complex mosaic of deforested and degraded forests interspersed with second-growth forests. Airborne lidar data were collected in four sites along the Cuiabá-Santarém highway, within or near the Tapajós National Forest.

- *Anambé base (ANA).* This site, within the Tapajós National Forest, was assigned as a forest concession for timber harvesting. Selective logging operations were carried out in 2015–2016 using reduced-impact techniques, and aimed at commercial species with DBH $\geq 55\,\text{cm}$ (Lei et al., 2018).

- *Km 67 base (TNF).* This site is located in one of the ecological corridors within the Tapajós National Forest limits. No indication of recent anthropogenic disturbance exists within this site, which is considered intact forest. However, there is evidence that this intact forest has been previously impacted by drought disturbances both during the 1990s

and during the 2015–2016 El Niño events (Hayek et al., 2018; Pyle et al., 2008; Leitold et al., 2018).

- *São Jorge (TSJ).* Forest near the settlement of São Jorge were originally within the boundaries of the Tapajós National Forest, but were excluded in 2012. Forests in this site constitute a mosaic of deforestation, fragmentation and degradation from fire and small-scale logging, with some areas experience secondary growth.

- *Eastern Sites (EBT).* The surveyed forests are located outside the Tapajós National Forest. The remaining forests are near several patches of pastures and croplands (mostly soy bean and maize), and thus fragmented and degraded. Logging activities have been occurring in this forests for at least 25 years, and some of the forests experienced one or multiple fires. Post-disturbance regrowth has been also observed in some forests.

3. *Paragominas, Brazil (PRG).* This region experienced significant expansion of selective logging starting in the 1960s, and became the largest center for hardwood processing in Brazil by the early 1990s (Veríssimo et al., 1992). Since then, agriculture and cattle ranching rapidly expanded, and became the main economic activity, and by the late 2000s about 45% of the forests in Paragominas had been cleared (Pinto et al., 2009). Three sites in the region were studied:

- *Cauaxi (CAU).* This site is privately managed by *Instituto Floresta Tropical*, and has been long used for teaching and training of reduced-impact logging techniques. About 800 ha were designated for logging from 2006 to 2012 (the year of the lidar survey used in this study). Nearly 600 ha of the forests were intact, including some areas within the logging work units that were in steep terrain (slope $> 20°$) and thus not suitable

for logging. Selective logging harvested commercial trees (DBH $\geq$ 55 cm) extracted 11–28 $m^3\,ha^{-1}$ of timber. Additional information in (Pinagé et al., 2019).

- *Andiroba (AND)*. This site is in a private land and has been partially deforested and remaining forests are moderately degraded and fragmented. Some of the remaining forests were logged (not using reduced-impact techniques) between 1999 and 2003. Understory forest fires affected the remaining forests in 2001 and 2009 (Longo et al., 2016).

- *Nova Neonita (PAR)*. This site, also in private landholding, is heavily degraded and fragmented. Some of the area was cleared in the 1980s and abandoned in the 1990s, leading to secondary growth. Other surveyed forests were damaged by intensive logging operations in the 1990s and in 2004–2006. Most of the remaining forests suffered extensive damage by three large fire events (1992, 2005, and 2008) (Morton et al., 2013; Longo et al., 2016).

4. *Feliz Natal, Brazil (FZN)*. Before logging operations became common in this region in the later 1970s, forests were minimally disturbed. In the 1980s, this region experienced significant changes with widespread deforestation, first for pastures, and later for croplands (mostly soy beans). Because this region has a distinct dry season and also experience episodes of low atmospheric humidity, forests in this region are prone to large, multi-day fires (Morton et al., 2013; Rappaport et al., 2018). Four sites were surveyed in this region:

- *Long transect (FN2)*. This long transect (50×0.2 km) sampled multiple areas with a broad range of disturbance histories typically found in FZN, including areas that were deforested, logged, burned and fragmented. Forest inventory plots were clustered in two segments, at 2 km north and 17 km south of the transect midpoint.

• *Vitória (FNA).* This site included some of the most degraded forests in our sample. Forests had been intensively logged in the 1990s, and experienced at least four severe fire events (2005, 2007, 2010, and 2012) (Morton et al., 2013). Biomass at the degraded forests was depleted by more than 90% relative to the nearby gallery forests (Longo et al., 2016).

• *Eastern site (FNC)..* Most forests in this site have been moderately degraded. Widespread selective logging disturbed parts of the surveyed forests between 1993–1996 or 2002–2005 (different locations). Some of the forests logged between 1993–1996 were affected by high-intensity fires in 1999, and since then have only experienced occasional low-intensity fires and additional logging.

• *Western site (FND)..* This area near this site has experienced significant deforestation since the 1980s, leading to highly fragmented forests. The surveyed forests are all within 1.5 km from the forest edge, and experienced high-intensity logging between 1993 and 2003. High-intensity, multi-day fires severely damaged some of the surveyed forests in 2007, 2010, and 2013, and created a range of forest structures due to gradients in disturbance exposure.

5. *Tanguro, Brazil (TAN).* Transitional forests in this region experience long (> 5 mo) and severe dry season and receive relatively low rainfall (1700 mm) (Balch et al., 2008). The surveyed forests include minimally disturbed forests and forests that are part of a fire experiment.

• *Fire experiment (TGE).* This experiment to understand the dynamics of forests under different fire regimes was established in 2004, when three 50 ha plots were set in the legal reserve of a private property. One plot remained as the control (never burned), one plot was burned every three years (2004, 2007, 2010), and one plot was burned 6

times (every year between 2004 and 2010, except 2008). Fires were set at late dry season (August-September) using fire lines about 100 m apart from each other during 3 or 4 days (reigniting them in case they were extinguished at night). Additional details can be found in (Balch et al., 2008; Brando et al., 2014). After 2010, plots were never burned again. In 2014, two eddy-covariance towers were installed at the experiment site: one within the footprint of the control plot, and another within the footprint of the burned plots (Brando et al., 2019).

- *Legal reserve (TGW).* This site includes areas immediately to the south and west of the fire experiment, also in the legal reserve of the Tanguro ranch. Surveyed forests generally do not show signs of recent disturbances despite being within 2 km of forest edges. These forests are considered mostly intact, with the exception of a 50 ha patch of forest that burned once in 2007.

S2. Additional information on airborne lidar and forest inventory plots

Each region contained one or multiple sites for which airborne lidar data were available. Many of these sites also contained forest inventory plots, and have been previously used in studies that quantified carbon losses due to degradation in the Amazon and plant area index estimation (Longo et al., 2016; Vincent et al., 2017; Rappaport et al., 2018). Table S2 provides additional information on each specific site. Further information on plots can be found in Gourlet-Fleury et al. (2004) (site PRC), Bonal et al. (2008) (site GFE), Brando et al. (2012) (site TGE), and Longo et al. (2016), Sustainable Landscapes Brazil (2019) and dos-Santos, Keller, and Morton (2019) (other sites). To reduce the differences among plots regarding size and sampling effort, we considered only living individuals (trees, lianas, and palms) with diameter at breast height $D \geq 10\,\mathrm{cm}$, and split larger

plots ($0.5 - 6.25$ ha) into sub-plots that were as close to 0.25 ha as possible. The location of all inventories in Brazil were geo-registered with sub-meter accuracy using differential Global Navigation Satellite Systems (GeoXH6000); forest inventories in French Guiana were geo-referenced with handheld Global Positioning System, with nominal accuracy of 2 m.

For the study areas in Brazil, airborne lidar data were collected between 2012 and 2017, and surveys used Optech ALTM instruments onboard an aircraft flying at average height of 850m above ground; the sensor scan angle was restricted to 5.6° off-nadir and an average swath sidelap between flight lines of 65% (Longo et al., 2016); the point cloud data are publicly available (Sustainable Landscapes Brazil, 2019). Airborne lidar data at GYF were collected in 2013; the aircraft flew at a height of 550m above ground carrying a Riegl LMSQ560; the scan angle was capped in 20° off-nadir, and the flight line sidelap was near 60% (Vincent et al., 2017). To ensure that the terrain elevation was well characterized, flights had to meet a minimum return density of $4\,\mathrm{m}^{-2}$ of 99.5% of the area (except water bodies and pastures), following previous recommendations for tropical forests (Leitold et al., 2015).

Some of the regions were only used to assist the calibration of the statistical models (Section S4.2), but not used in the simulations. Because our goal was to characterize the impacts of degradation on forest structure and ecosystem functioning, we did not include simulations from MAO, where all surveyed forests were intact, nor did we include JAM and FST, where all forests were logged (albeit using reduced-impact techniques). Forests in SFX were not included because the disturbance history based on Landsat analysis was uncertain due to widespread presence of vines. Finally, at RBR, none of the

surveyed forests could be considered intact or logged using reduced-impact techniques, which precluded us to have a minimally-disturbed forest as reference.

S3. Additional ED-2.2 developments

S3.1. Allometric relations

To obtain an allometric equation for diameter at breast height (D, cm) as a function of tree height (H, m), we used all individual tree measurements from the plots included in steps 1 and 2 that were from living trees (excluding lianas and palms), and had field measurements of both D and H ($n = 15865$). Because the sampling effort was not even across tree sizes, and to reduce the effects of variability in tree measurements of height along the D range on local biases, we followed the approach by Jucker et al. (2017) and binned the data into 50 evenly spaced $\log_{\mathrm{e}}(D)$ classes between $D = 5$ and $D = 200\,\mathrm{cm}$ (the range of D measurements). The binned data were fitted using standardized major axis regression. This choice ensures that the arithmetic inverse relationship (i.e. height as a function of D) could be also used in the ED-2.2 model:

$$\log_{\mathrm{e}}(D) = \underbrace{(-2.01 \pm 0.25)}_{\log_{\mathrm{e}}(d_1)} + \underbrace{(1.68 \pm 0.08)}_{d_2} \log_{\mathrm{e}}(H), \qquad \text{(S1)}$$

where H should be in m, and D should be in cm. The model fit is shown in Figure S16a.

We did not have any measurement of individual leaf area (L_i, $\mathrm{m}^2_{\mathrm{Leaf}}\,\mathrm{plant}^{-1}$) at the study sites, therefore we developed an allometric equation based on the Biomass And Allometry Database (BAAD; Falster et al., 2015). Similar to many allometric equations for aboveground and leaf biomass (e.g., Chave et al., 2014), we used ($D^2\,H$) as the predictor. Because we did not seek a reversible equation, we fitted the model using minimum least squares with heteroskedastic distribution of residuals (Mascaro et al., 2011; Longo et al.,

2016). The fitted model was:

$$L_i = \underbrace{(0.234 \pm 0.012)}_{\ell_1} \left(D^2\, H\right)^{\overbrace{0.641 \pm 0.011}^{\ell_2}} + E_{\mathcal{N}}\left[\mu = 0, \sigma = 0.241 \pm 0.026\, L_i^{1.001 \pm 0.056}\right], \tag{S2}$$

where coefficients are presented in the form Expected Value ± Standard Error; units for the empirical equation should be: D in cm, H in m, and L_i in $\mathrm{m^2_{Leaf}\,plant^{-1}}$. The model fit is shown in Figure S16b.

In ED-2.2, the carbon stocks ($\mathrm{kgC\,plant^{-1}}$) of different tissues — leaves (C_L), fine roots (C_R), sapwood (C_S), bark (C_B) and heartwood (C_H) — are defined through allometric equations. Leaf biomass (C_L) is obtained from Equation (S2):

$$C_L = \frac{L_i}{\mathrm{SLA}}, \tag{S3}$$

where SLA ($\mathrm{m^2_{Leaf}\,kgC^{-1}}$) is the individual plant's specific leaf area. Fine-root biomass and sapwood biomass are derived from leaf biomass, using the same relationships described in Moorcroft, Hurtt, and Pacala (2001). Bark biomass followed a parameterization similar to sapwood:

$$C_R = q_R\, C_L, \tag{S4}$$

$$C_S = q_S\, H\, C_L, \tag{S5}$$

$$C_B = q_B\, H\, C_L, \tag{S6}$$

where $q_R = 1$ for all plant functional types, following Moorcroft et al. (2001). The leaf-to-sapwood (q_S) and leaf-to-bark (q_B) scaling factors ($\mathrm{m^{-1}}$) are determined using the same formulation as Falster, FitzJohn, Brännström, Dieckmann, and Westoby (2016):

$$q_S = \frac{\eta_c\, \mathrm{SLA}\, \rho_W\, 1000}{\beta\, A_{L:S}}, \tag{S7}$$

$$q_B = \frac{\eta_c \, \mathrm{SLA} \, \rho_B \, 1000}{\beta \, A_{L:B}} \qquad \text{(S8)}$$

where η_c is an empirical shape parameter based on Falster et al. (2016) parameterization for broadleaf trees; $A_{L:S}$ and $A_{L:B}$ ($\mathrm{m^2_{Leaf}\,m^{-2}_{Bark}}$) are the leaf:sapwood and leaf:bark area ratios, respectively; ρ_W and ρ_B ($\mathrm{g\,cm^{-3}}$) are the wood and bark densities, respectively; $\beta = 2.0\,\mathrm{kg\,kgC^{-1}}$ is the oven-dry:carbon biomass ratio; and the factor 1000 is included for unit conversion. Values of these parameters are shown in Table S3.

The allometric equation for heartwood biomass (C_H) was obtained using both the pantropical allometric equation for aboveground biomass (C_{AG}, $\mathrm{kgC\,plant^{-1}}$; Chave et al., 2014), and that total aboveground biomass is the sum of the biomass of the following tissues:

$$C_{\mathrm{AG}} = \frac{1}{\beta} 0.0673 \left(\rho_W \, D^2 \, H\right)^{0.976} \qquad \text{(from Chave et al., 2014)} \qquad \text{(S9)}$$

$$C_{\mathrm{AG}} = C_L + f_{\mathrm{AG}} \left(C_S + C_B + C_H\right), \qquad \text{(S10)}$$

where f_{AG} is the fraction of biomass above ground; $\beta = 2.0\,\mathrm{kg\,kgC^{-1}}$ is the oven-dry:carbon biomass ratio; and units for S9 should be: ρ_W in $\mathrm{g\,cm^{-3}}$, D in cm, H in m, and C_{AG} in $\mathrm{kgC\,plant^{-1}}$. To simplify the implementation of C_H in ED-2.2, we used Equations (S9), (S10) and (S1) to find C_H at $D = 10\,\mathrm{cm}$ (typical minimum diameter measured in inventories) and at $H = 46\,\mathrm{m}$ (maximum height allowed for tropical trees) and derive a function for C_H with the same form and units as Equation (S9):

$$C_H = \frac{1}{\beta} 0.0608 \left(\rho_W \, D^2 \, H\right)^{1.004} . \qquad \text{(S11)}$$

S3.2. Changes in the photosynthesis module

The photosynthesis module in ED-2.2 has been previously described in detail in (Longo, Knox, Medvigy, et al., 2019); here we show only a brief overview and highlight the

main modifications. Similarly to previous versions, the net CO_2 assimilation rate (A, $\mathrm{mol CO_2\, m^{-2}\, s^{-1}}$) for C_3 plants is defined as:

$$A = V_c - \frac{1}{2} V_o - R, \quad \text{(S12)}$$

$$V_o = \frac{2\,\Gamma}{c_i} V_c, \quad \text{(S13)}$$

$$\Gamma = \frac{o}{2\,\tau}, \quad \text{(S14)}$$

where V_c, V_o, and R ($\mathrm{mol CO_2\, m^{-2}\, s^{-1}}$) are the carboxylation, oxygenation (photorespiration) and day respiration rates, respectively; Γ ($\mathrm{mol\, CO_2\, mol^{-1}}$) is the CO_2 compensation point; ($\mathrm{mol\, CO_2\, mol^{-1}}$) is the intercellular $o = 0.209\,\mathrm{mol O_2\, mol^{-1}}$ is the oxygen mixing ratio; and τ is the carboxylase:oxygenase ratio. The terms R, Γ, and τ are calculated the same way as in (Longo, Knox, Medvigy, et al., 2019). The carboxylation rate V_c depends on environmental constraints, which ultimately limits the net assimilation rate A.

The maximum carboxylation rate given temperature ($V_c^{\max}$) is defined as in Longo, Knox, Medvigy, et al. (2019):

$$V_c^{\max} = \frac{V_{c15}^{\max}\, Q_V^{\frac{T-T_{15}}{10}}}{\{1 + \exp\left[-f\ (T - T_\mathrm{c})\right]\}\ \{1 + \exp\left[+f\ (T - T_\mathrm{h})\right]\}}, \quad \text{(S15)}$$

where $V_{c15}^{\max}$ ($\mathrm{mol\, m^{-2}\, s^{-1}}$) is $V_c^{\max}$ at temperature $T_{15} = 288.15\,\mathrm{K}$ ($15°C$); T (K) is the leaf temperature; Q_V determines the steepness of the temperature dependence of $V_c^{\max}$; f, T_c, and T_h are phenomenological parameters that reduce $V_c^{\max}$ at extreme temperatures, following the same formulation used in previous ED versions (Moorcroft et al., 2001; Longo, Knox, Medvigy, et al., 2019).

The maximum carboxylation rate can never be achieved because CO_2 inhibts oxygenation, and O_2 inhibits carboxylation (von Caemmerer, 2000). The carboxylation rate at

saturated Ribulose-1,5-Biphosphate (RuBP) conditions (V_c^{RuBP}) is determined as:

$$V_c^{\mathrm{RuBP}} = V_c^{\max} \frac{c_i}{c_i + K_c \left(1 + \dfrac{o}{K_o}\right)}, \tag{S16}$$

where K_c ($\mathrm{mol CO_2\, mol^{-1}}$) and K_o ($\mathrm{mol O_2\, mol^{-1}}$) are the Michaelis constants for carboxylation and oxygenation, respectively, and are also calculated as in (Longo, Knox, Medvigy, et al., 2019). Equation (S16) is the same described in (Longo, Knox, Medvigy, et al., 2019).

The RuBP regeneration depends on the electric transport rate (J, $\mathrm{mol\, m^{-2}\, s^{-1}}$), which in turns depends on the absorbed irradiance (I, $\mathrm{mol\, m^{-2}\, s^{-1}}$). If I is relatively low, then RuBP pools may decline, limiting the carboxylation rate. The RuBP-limited (also known as light-limited) carboxylation rate (V_c^{PAR}) is defined as in von Caemmerer (2000):

$$V_c^{\mathrm{PAR}} = \frac{J}{4 + 8\,\dfrac{\Gamma}{c_i}}, \tag{S17}$$

and J is determined from an empirical quadratic equation (von Caemmerer, 2000; Oleson et al., 2013):

$$J = \frac{(I_{\mathrm{PSII}} + J^{\max}) - \left[(I_{\mathrm{PSII}} + J^{\max})^2 - 4\,\varphi\, I_{\mathrm{PSII}}\, J^{\max}\right]^{\frac{1}{2}}}{2\,\varphi} \tag{S18}$$

$$J^{\max} = \frac{J_{15}^{\max}\, Q_J^{\frac{T-T_{15}}{10}}}{\{1 + \exp\left[-f_c\,(T - T_c)\right]\}\,\{1 + \exp\left[+f_h\,(T - T_{\mathrm{h}})\right]\}} \tag{S19}$$

$$I_{\mathrm{PSII}} = \frac{1}{2}\,\gamma_{\mathrm{PSII}}\, I \tag{S20}$$

where $J^{\max}$ ($\mathrm{mol\, m^{-2}\, s^{-1}}$) is the temperature-dependent maximum electron transport rate; $J_{15}^{\max}$ and Q_J are the equivalent of $V_{c15}^{\max}$ and Q_V for the electron transport rate, respectively; I_{PSII} ($\mathrm{mol\, m^{-2}\, s^{-1}}$) is the light effectively used by the photosystem II; $\varphi = 0.7$ is an empirical curvature parameter (von Caemmerer, 2000; Oleson et al., 2013); $\gamma_{\mathrm{PSII}} = 0.85$ is the quantum yield of the photosystem II (von Caemmerer, 2000; Oleson et al., 2013);

and T_c, T_h, f_c, and f_h are empirical parameters to downscale photosynthetic activity at extreme temperatures (Table S3). Unlike the original implementation of V_c^{PAR} (Moorcroft et al., 2001; Longo, Knox, Medvigy, et al., 2019) the explicit representation on electron transport rate is advantageous because it accounts for the differences in temperature dependence of $J^{\max}$ and $V_c^{\max}$ (von Caemmerer, 2000), and the saturation behavior of J as I becomes non-limiting.

In addition to light limitation, carboxylation rates may be limited by the triose phosphate utilization (TPU) for synthesizing sugars and starch (von Caemmerer, 2000). The TPU limitation typically occurs when both CO_2 mixing ratio and irradiance are high, or when temperature is low (von Caemmerer, 2000; Lombardozzi et al., 2018), and is expected to become more important as atmospheric CO_2 increases (Lombardozzi et al., 2018). The TPU-limited carboxylation rate (V_c^{TPU}) is defined as:

$$V_c^{\mathrm{TPU}} = 3\,E_{\mathrm{TP}}\,\frac{c_i}{c_i - \Gamma}, \tag{S21}$$

where E_{TP} ($\mathrm{mol\,m^{-2}\,s^{-1}}$) is the export rate of triose phosphate from chloroplasts, and is normally parameterized as a function of $V_c^{\max}$ ($E_{\mathrm{TP}} = \varepsilon_E\,V_c^{\max}$; von Caemmerer, 2000; Oleson et al., 2013; Lombardozzi et al., 2018).

Similar to previous versions of ED-2, the net assimilation rate is determined through a law of minimum:

$$A = \min\left(A^{\mathrm{RuBP}}, A^{\mathrm{PAR}}, A^{\mathrm{TPU}}\right) \tag{S22}$$

where each of the cases on the right-hand side are calculated from Equations (S12) and (S13), by replacing V_c with each of the cases (Equations S16, S17, and S21), and using the algorithm described in Longo, Knox, Medvigy, et al. (2019).

Both $J_{15}^{\max}$ and E_{TP} are assumed to be proportional to $V_{c15}^{\max}$. To obtain the proportionality ratios, we used the data collected at multiple sites in Panama (Gu et al., 2016; Norby et al., 2017). Even though the Norby et al. (2017) provided fits relating these quantities, we refitted the functions to eliminate the intercept, and corrected for the fact that Norby et al. (2017) provides values at 25°C and ED-2.2 needs the reference at 15°C:

$V_c^{\max}$ ($J_{15}^{\max} = \varepsilon_J \, V_{c15}^{\max}$)

The values of ε_J and ε_E are determined from the data collected at multiple sites in Panama and described in Norby et al. (2017). Although Norby et al. (2017) provided empirical fits relating $V_c^{\max}$, $J^{\max}$ and E_{TP}, we obtained the relationships using standardized major axis (SMA) to account for the variability on both variables, and corrected for the fact that Norby et al. (2017) values use a different reference temperature (25°C):

$$\varepsilon_J = \underbrace{\frac{J_{25}^{\max}}{V_{c25}^{\max}}}_{\varepsilon'_J} \frac{Q_V}{Q_J}, \tag{S23}$$

$$\varepsilon E = \frac{E_{\mathrm{TP}}}{V_{c25}^{\max}}, \tag{S24}$$

where $J_{25}^{\max}$ and $V_{c25}^{\max}$ are the values at 25°C, obtained directly from Gu et al. (2016). The SMA line, coefficients ε'_J and ε_E and the R^2 are shown in Figure S17.

S3.3. Updated trait and trade-off relationships

In ED-2.2, we represent the functional diversity within ecosystems by defining multiple plant functional types (PFTs). PFTs are defined by both morphological characteristics (e.g. tree or grass) and by a set of traits that determine a variety of life strategies within the ecosystems. Many traits and trade-offs of tropical forest PFTs had not been changed since the original ED-1.0 release (Moorcroft et al., 2001), despite the increase in data availability for the tropics. Here, we aggregated data from multiple trait-based studies

and trait data bases such as GLOPNET and TRY (Wright et al., 2004; Santiago & Wright, 2007; Chave et al., 2009; Kattge et al., 2009, 2011, 2020; Baraloto et al., 2010; Powers & Tiffin, 2010; Bahar et al., 2017; Norby et al., 2017), to revise the values associated with each PFT. For this revision, we focused on the following traits: wood density, leaf turnover rate, specific leaf area, leaf carbon:nitrogen ratio, maximum carboxylation rate, maximum electron transport rate, and maximum triose-phosphate utilization rate. These traits were selected because we obtained a sufficiently large ($n > 50$) number of samples that could be used to build trade-off relationships and were already used to define trade-offs in ED-2.2, and traits known to directly or indirectly influence gross primary productivity and thus light- and water-use efficiencies. To remove confounding factors such as canopy position, we only used data for sun leaves, or individuals that were either emergent or canopy trees.

Wood density was the most widely available trait in our data base, and also the indicative trait used to define PFTs in ED-1.0 (Moorcroft et al., 2001). To re-define the PFTs, we used the data from all forest inventory plots available, attributed wood density for each individual using the wood density data base compiled by Chave et al. (2009). We then calculated the probability distribution function of wood density (weighted by basal area), and split the distribution based on quantiles (the lower, middle, and upper 33% of the distribution associated with early-successional, mid-successional, and late-succesional trees, respectively). The expected values of wood density for each PFT was assumed to be the mid-point within each quantile (i.e. 16.67%, 50%, and 83.33% quantiles, respectively).

To determine the trade-off axes between traits, we fitted standardized major axes (SMA). Because most wood density data came from the Chave et al. (2009) compilation (only wood density data were available), we aggregated data to species to seek relation-

ships between wood density and other traits. Most traits were not correlated with wood density: leaf turnover rate showed the most significant, yet weak correlation with wood density (Figure S18a). For leaf traits, we were able to obtain large number of paired observations (i.e. two trait measurements from the same individual) between specific leaf area (SLA) and the other traits, and thus we fitted the standardized major axes using SLA as one of the variables (Figures S18b, S18c, and S18d).

The revised trait values for the plant functional types used in these simulations are shown in Table S3.

S4. ED-2.2 initial conditions using airborne lidar

The approach to obtain initial conditions for ED-2 using airborne lidar data is summarized in three steps: (1) derivation of unscaled vertical profiles of leaf area density from the vertical distribution of returns, and the height-dependent proportion of leaf area density allocated to each plant functional type; (2) estimation of plot-level properties of the forest (biomass, basal area, and individual's stem density) from airborne lidar; (3) optimization of scaling factors to obtain absolute leaf area density profiles and the initial conditions for ED-2. This approach requires only representative, geo-referenced forest inventory plots for calibration, and small-footprint, discrete-return airborne lidar point cloud data with high density of returns, in addition to knowledge of individual-based allometric equations that relate diameter at breast height (D) to tree height, above-ground biomass and leaf biomass.

S4.1. Vertical foliage profiles

To obtain vertical profiles of leaf area density (Figure 2, Box 1) across the areas surveyed by airborne lidar, we first clipped the full point cloud domain into $50 \times 50\,\mathrm{m}$ columns. For each column, we simulated a pseudo-waveform from the discrete point clouds to create a continuous and smooth distribution of return energy in the vertical (see one example in Figure S19a). Our simulated waveform function (E) is based on the algorithm described by Popescu, Zhao, Neuenschwander, and Lin (2011) and Hancock et al. (2019):

$$E\left(h_i\right) = X\left(h\right) * Z\left(h, h_i\right), \tag{S25}$$

$$X\left(h\right) = \sum_{n=1}^{N} \begin{cases} 1 & \text{if } h_n \in \left[h - \dfrac{\Delta h}{2}; h + \dfrac{\Delta h}{2}\right] \\ 0 & \text{otherwise} \end{cases}, \tag{S26}$$

$$Z\left(h, h_i\right) = \frac{1}{\sigma_h \sqrt{2\,\pi}} \exp\left[-\frac{\left(h - h_i\right)^2}{2\,\sigma_h^2}\right], \tag{S27}$$

where h_i is the mean elevation of each bin; $\Delta h = 10\,\mathrm{cm}$ is the thickness of each bin layer; $X\left(h\right)$ is the energy distribution function across the laser beam trajectory (horizontal); $Z\left(h\right)$ is the energy distribution function in the vertical (i.e. along the laser beam trajectory); σ_z is the pulse width in the vertical, which controls the smoothness of the simulated waveform; and $*$ is the convolution operator. Similar to Hancock et al. (2019), we binned the return counts before applying the convolution to improve computational efficiency. When the goal is to simulate the signal of large-footprint waveform lidar (e.g. GLAS or GEDI), the energy distribution function across the laser beam trajectory is frequently assumed Gaussian (Blair & Hofton, 1999; Popescu et al., 2011; Hancock et al., 2019). In our case, however, we sought to characterize the average vegetation profile for the entire column and assumed a uniform (rectangular) distribution across the entire column area instead (Eq. S26). In addition, as we will discuss in later in this text, it is important

that the waveform is not excessively noisy to obtain realistic leaf area index, yet it should retain sufficient features to ensure the vegetation structure is not overly aggregated (Figure S19a). We defined $\sigma_h = 50\,\mathrm{cm}$ which resulted in a good compromise in preliminary tests. Finally, following Hancock et al. (2019), we calculated the waveform functions for vegetation (E_v) and ground (E_g) returns separately, in order to obtain the integrated return energy (R_v and R_g):

$$R_v\left(h_i\right) = \sum_{j=i}^{N_I} E_v\left(h_j\right), \tag{S28}$$

$$R_g = \sum_{j=1}^{N_I} E_g\left(h_j\right), \tag{S29}$$

where N_I is the total number of layers. In our case, we defined layers up to $h_T = 70\,\mathrm{m}$ to ensure that the tallest sampled trees would be completely characterized.

To obtain the relative vertical distribution of leaf area density ($\lambda\left(h\right)$; $\mathrm{m}^2_{\mathrm{Leaf}}\,\mathrm{m}^{-2}$), we applied the Beer-Lambert light extinction approach, following the approach originally developed by MacArthur and Horn (1969) and adapted for lidar profiles (e.g., Ni-Meister et al., 2001; Stark et al., 2012; Antonarakis et al., 2014). In this approach, $\lambda\left(h\right)$ is a function of the gap probability (P, non-dimensional):

$$\lambda(h) = \frac{\cos\varphi}{G\left(h,\varphi\right)} \frac{1}{P\left(h,\varphi\right)} \frac{\partial P\left(h,\varphi\right)}{\partial h}, \tag{S30}$$

where h is the height, φ is the angle of incident light, and $G\left(h,\varphi\right)$ is the leaf area projection factor. For most lidar surveys used in this study, the maximum off-nadir scan angle was 5.5° (Longo et al., 2016); the only exception was Paracou (GYF), where the off-nadir angle was 20° (Vincent et al., 2017). As a first approximation, we assumed $\varphi \approx 0$, and thus $P\left(h,\varphi\right) \approx P\left(h\right)$, but we acknowledge that this introduces an error ($5-8\%$ for 10% of the points at GYF). The leaf area projection factor is dependent upon the mean leaf

orientation. For simplicity, we assumed isotropic (random) orientation, i.e. $G(h, \varphi) = 0.5$ (Ni-Meister et al., 2001; Vincent et al., 2017).

Following Ni-Meister et al. (2001), the vertical profile of gap probability can be described by the integral of the lidar return energy $[R_v(h)]$ between height h and the top canopy height (h_T):

$$-\frac{\mathrm{d}R_v(h)}{\mathrm{d}h} = J_0\, r_v \frac{\mathrm{d}P(h)}{\mathrm{d}h}, \tag{S31}$$

where J_0 is the irradiance emitted by the lidar sensor and r_v is the canopy reflectivity. Using the boundary conditions at the top canopy $[R_v(h_T) = 0; P(h_T) = 1]$ and that the total energy reflected by the ground is proportional to the total gap fraction, we obtain:

$$R_v(h_i) = J_0\, r_v \left[1 - P(h_i)\right], \tag{S32}$$

$$R_{v0} = J_0\, r_v \left[1 - P(h = 0)\right], \tag{S33}$$

$$R_g = J_0\, r_g\, P(h = 0), \tag{S34}$$

where r_g is the soil reflectivity and $R_{v0} = R_v(h = 0)$. The irradiance emitted by the sensor (J_0) is not provided in the data set, however it is possible to combine Equations (S32)-(S34) to suppress J_0 from the definition of $P(h)$:

$$P(h_i) = 1 - \frac{R_v(h_i)}{R_{v0} + k_r\, R_g}, \tag{S35}$$

where $k_r = \frac{r_v}{r_g}$, the ratio between vegetation and ground reflectivities. By substituting Equations (S31), (S33), and (S35) into Equation (S30) for the $\varphi = 0; G = 0.5$ case, we obtain:

$$\lambda(h) = 2\frac{\mathrm{d}}{\mathrm{d}h}\ln\left[R_{v0} + k_r\, R_g - R_v(h)\right]. \tag{S36}$$

It is possible to determine k_r from airborne lidar surveys that have reflectance data (Antonarakis et al., 2014), or from optimization using independent local measurements of leaf area index (Stark et al., 2012). Neither information is easily obtained for large areas, and thus we assumed $k_r = 1.03$, following Tang and Dubayah (2017). We found that the results are not sensitive to small variations in k_r, particularly when the gap fraction is low. On the other hand, the approximation of return counts is only a proxy to the return energy, and therefore, we assumed that the profile obtained from Equation (S36) was considered unscaled, and will be referred as $\lambda^{\star}(h)$. Following Shao, Stark, de Almeida, and Smith (2019), we excluded the profile below 5 m, as estimates of leaf area density near the surface often show large uncertainty due to the limited fraction of returns near the surface in denser canopies.

Cohorts in ED-2 are defined as discrete groups of individuals with similar size and same life strategy (plant functional type; PFT). To separate the vertical profile into discrete layers of similar size, we assumed that the layers with the most significant population can be identified by local maxima, or by local saddle points when the layers are not completely separated, as shown in Figure S19b. The boundary between consecutive layers is defined as either the local minima or inflection points that are not saddle points (Figure S19b). These features were automatically determined based on the function `peaks` (package `RSEIS`, Lees, 2017), which was modified to capture inflection points and local minima.

The last stage of step 1 was to attribute the fraction of each plant functional type in each vertical layer, which was used to define the cohorts (Figure S19c). Because the airborne lidar data was from a single band, we could not use spectral mixture analyses (e.g., Antonarakis et al., 2014). To overcome this limitation, we also simulated waveforms

for all plots that had complete overlap with airborne lidar data in all of the study sites, and complemented with data from the Sustainable Landscapes Brazil project (Longo et al., 2016; Sustainable Landscapes Brazil, 2019; dos-Santos et al., 2019) (total of 817 $0.25 - ha$ plots). For each plot, we determined the expected relative proportion of each PFT p (early-successional, ETR; mid-successional, MTR; and late-successional, LTR) as a function of height ($q_p(h)$) and the associated profile of return heights and built a look-up table. The normalized profile of each column was compared with the normalized profile of all plots in the look-up table using the Kolmogorov-Smirnov test, and the least dissimilar profile found in the look-up table was used to determine the relative proportion of PFTs in the column of interest (Figure S19c).

S4.2. Statistical models for plot-level properties

For the second step (Figure 2, Box 2), we developed parametric statistical models that related summary metrics describing the distribution of return heights with four plot-level properties ($D \geq 10\,\mathrm{cm}$): aboveground biomass carbon density (ABCD, $\mathrm{kg_C\,m^{-2}}$), basal area (BA, $\mathrm{cm^2\,m^{-2}}$), (maximum, allometry-based) leaf area index (LAI, $\mathrm{m^2_{Leaf}\,m^{-2}}$), and stem number density (ND, $\mathrm{m^{-2}}$). Similar to Step 1 (Section S4.1), we considered again all plots that were entirely within the areas surveyed by airborne lidar (total of 817 $0.25 - \mathrm{ha}$ plots, Section 4). For each plot-level property, we selected the most informative yet simple model using the subset selection of regression method method (Miller, 1984). Additionally, we only considered models that did not show strong signs of multicollinearity, quantified by the variance inflation factor (VIF < 4). The selected model was fitted assuming heteroskedastic distribution of residuals (Mascaro et al., 2011; Longo et al., 2016). Field inventory above-ground biomass was determined using the same models as in Longo et al.

(2016). Individual-based maximum leaf area was determined using an allometric model derived from the Biomass And Allometry Database (BAAD; Falster et al., 2015) and presented in Section S4.3.

We obtained the following models:

$$\begin{aligned}
\mathrm{ABCD}_{\mathrm{ALS}} &= 0.132^{+0.072}_{-0.045}\ \mu_h^{1.59^{+0.14}_{-0.14}} \\
&\quad + E_{\mathcal{N}}\left[\mu = 0, \sigma = 0.95^{+0.35}_{-0.25}\ \mathrm{ABCD}_{\mathrm{ALS}}^{0.49^{+0.15}_{-0.13}}\right], && \text{(S37)} \\
\mathrm{BA}_{\mathrm{ALS}} &= 1.81^{+1.19}_{-0.65}\ \exp\left[-5.77^{+1.19}_{-0.94}\ f_{1-2.5}\right] h_{75}^{0.85^{+0.12}_{-0.15}} \\
&\quad + E_{\mathcal{N}}\left[\mu = 0, \sigma = 1.45^{+1.54}_{-0.39}\ \mathrm{BA}_{\mathrm{ALS}}^{0.39^{+0.16}_{-0.26}}\right], && \text{(S38)} \\
\mathrm{LAI}_{\mathrm{ALS}} &= 0.37^{+0.33}_{-0.13}\ \exp\left[-5.8^{+1.7}_{-2.0}\ f_{1-2.5}\right] \mu_h^{0.91^{+0.12}_{-0.20}} \\
&\quad + E_{\mathcal{N}}\left[\mu = 0, \sigma = 0.462^{+0.141}_{-0.045}\ \mathrm{LAI}_{\mathrm{ALS}}^{0.49^{+0.14}_{-0.22}}\right], && \text{(S39)} \\
ND_{\mathrm{ALS}} &= 0.0337^{+0.0053}_{-0.0083}\ \exp\left[-8.5^{+2.0}_{-1.8}\ f_{1-2.5} + 0.77^{+0.31}_{-0.17}\, F_{7.5}\right] \\
&\quad + E_{\mathcal{N}}\left[\mu = 0, \sigma = 0.038^{+0.069}_{-0.027}\ \mathrm{ND}_{\mathrm{ALS}}^{0.37^{+0.26}_{-0.40}}\right], && \text{(S40)}
\end{aligned}$$

where $f_{1-2.5}$ is the fraction (range $0.0 - 1.0$) of returns coming from the layer between 1 and 2.5 m; $F_{7.5}$ is the fraction (range $0.0 - 1.0$) of returns from above 7.5 m; h_{75} is the third quartile of the distribution of return heights; and μ_h is the mean of the distribution of return heights. Numbers after the coefficients are the 68% range (equivalent to $\pm 1\sigma$ if the distribution was Gaussian) of 1000 replicates using a nested bootstrap sampling. We separated the plots by study regions, then for each replicate, we first randomly selected which study regions to include in the model fitting stage, then randomly selected plots from the these regions. Plots from regions excluded from the model fitting stage were used for cross-validation.

The fitted models for ABCD, BA, and LAI showed similar-quality fits, and both explained over 70% of the inventory-plot variance (Table S4), whereas the model for ND explained 64% of the observed variance (Figure S20c; Table S4). Cross-validation assessment show that all fitted models are robust: models show similar fraction of unexplained variance, and none of them are significantly biased (Figure S20; Table S4).

S4.3. Plot-specific scaling factors and absolute cohort demography

For the third step of this approach (Figure 2, box 3), the unscaled profiles obtained in step 1 were calibrated using the stem number density (ND), basal area (BA) and aboveground biomass carbon density (ABCD) estimated from the parametric models developed in step 2. First, we obtain the unscaled leaf area index of each cohort layer i ($\Lambda_i^\star$):

$$\Lambda_i^\star = \int_{h_i^-}^{h_i^+} \lambda^\star (h) \, \mathrm{d}h, \qquad \text{(S41)}$$

where $\left(h_i^-; h_i^+\right)$ are the lower and upper bounds of the discrete layer associated with cohort i (Figure S19). We then estimated the unscaled stem number density of cohort i ($n_i^\star$, m^{-2}) following the same approach by Antonarakis et al. (2014), which assumes that the leaf area index is directly proportional to $n_i^\star$, and individual leaf area (L_i, $\mathrm{m}^2_{\mathrm{Leaf}}\,\mathrm{plant}^{-1}$), assumed to be a function of the tree size:

$$n_i^\star = \frac{1}{L_i \left(D_i, H_{t_i}\right)} \, \Lambda_i^\star, \qquad \text{(S42)}$$

where D_i (cm) is the diameter at breast height, and H (m) is the tree height. Neither L_i nor D_i can be directly retrieved by airborne lidar, therefore we developed allometric equations based on available data. To be consistent with the ED-2.2 simulations, we used the allometric equations for height and individual leaf area described in Supplement S3.1.

The unscaled stem number density of each cohort ($n_i^\star$) is obtained by substituting Equations (S2) and (S1) into Equation (S42):

$$n_i^\star = \nu_1 \, H^{\nu_2} \, \Lambda_i^\star, \tag{S43}$$

$$\nu_1 = \frac{1}{\ell_1 \, d_1^{2\,\ell_2}}, \tag{S44}$$

$$\nu_2 = -\left(2\,d_2 + 1\right) \, \ell_2. \tag{S45}$$

Once all $n_i^\star$ values are determined, it is possible to derive unscaled, column-aggregated values of aboveground biomass carbon density ($\mathrm{ABCD}^\star$), basal area ($\mathrm{BA}^\star$) and stem number density ($\mathrm{ND}^\star$):

$$\mathrm{ABCD}^\star = \sum_{i=1}^{I} \left(n_i^\star \, f_C \, a_1 \, \left\{ \rho_{p(i)} \, \left[D\left(H\right)\right]^2 \, H \right\}^2 \right), \tag{S46}$$

$$\mathrm{BA}^\star = \sum_{i=1}^{I} \left\{ n_i^\star \, \frac{\pi}{4} \, \left[D\left(H\right)\right]^2 \right\}, \tag{S47}$$

$$\mathrm{LAI}^\star = \sum_{i=1}^{I} \left\{ n_i^\star \, \Lambda_i^\star \right\}, \tag{S48}$$

$$\mathrm{ND}^\star = \sum_{i=1}^{I} n_i^\star, \tag{S49}$$

where I is the total number of cohorts in the analyzed column, $(\rho_{\mathrm{ETR}}; \rho_{\mathrm{MTR}}; \rho_{\mathrm{LTR}}) = (0.450; 0.615; 0.790)\ \mathrm{g\,cm^{-3}}$ are the wood density values for each PFT $p(i)$, and $(a_1; a_2) = (0.0673; 0.976)$ are the empirical coefficients from the pantropical allometric equation developed by Chave et al. (2014). The unscaled values are compared with the properties estimated using the statistical model using airborne-lidar metrics (Section S4.2), denoted by $\left(\mathrm{ND}^\odot; \mathrm{BA}^\odot; \mathrm{LAI}^\odot; \mathrm{ABCD}^\odot\right)$:

$$e_A = \frac{\mathrm{ABCD}^\odot}{\mathrm{ABCD}^\star}, \tag{S50}$$

$$e_B = \frac{\mathrm{BA}^\odot}{\mathrm{BA}^\star}, \tag{S51}$$

$$e_L = \frac{\mathrm{LAI}^\odot}{\mathrm{LAI}^\star}, \tag{S52}$$

$$e_N = \frac{\mathrm{ND}^{\odot}}{\mathrm{ND}^{\star}}, \tag{S53}$$

where $(e_A; e_B; e_L; e_N)$ are the scaling factor that would match the estimates from the third step with estimates from the first step. The minimum overall error when taking all variables into account can be determined from the global minimum of function S based on the weighted least squares:

$$S(e) = \frac{w_A\,(e - e_A)^2 + w_B\,(e - e_B)^2 + w_L\,(e - e_L)^2 + w_N\,(e - e_N)^2}{w_A + w_B + w_L + w_N}, \tag{S54}$$

where $(w_A; w_B; w_L; w_N) = (0.279; 0.251; 0.292; 0.177)$ are the weights of ABCD, BA, LAI, and ND, respectively, and are proportional to the inverse of the fraction of unexplained variance for the full model (Table S4). The scaling factor e that minimizes can be determined analytically:

$$e = \frac{w_A\,e_A + w_B\,e_B + w_L\,e_L + w_N\,e_N}{w_A + w_B + w_L + w_N}, \tag{S55}$$

which is equivalent to the weighted average of the scaling factors. The scaled number density of each cohort i is then assumed to be $n_i = e\,n_i^{\star}$.

S4.4. General scaling factor

The scaling factor in step 3 (Equation S55) could be obtained for any airborne lidar column, as it only relies on the local vertical profile of returns (Section S4.1) and statistical models based on airborne lidar metrics (Equations S37–S40). However, the statistical models (Equations S37–S40) are based on plots with $D \geq 10\,\mathrm{cm}$, which is relatively high for the most degraded forests. Consequently, the statistical models cannot fully constrain the leaf area density profiles at the most degraded forests, because the return energy above 11 m (equivalent to $D \geq 10\,\mathrm{cm}$) may represent a small fraction of the return energy. To overcome this limitation introduced by the lack of small trees in our forest inventory

data set, we sought to define a characteristic scaling factor that could be applied to all lidar scenes. To do so, we used the results from the regional cross validation at all sites (Table S2) to analyze the distribution of scaling factors e. The distribution of factors from all the plots are shown in Figure S21. The distribution has a well-defined peak, and the mode of the global distribution is close to the median value $e_{50} = 1.357$. Although the distribution of factors vary by each site (Figure S21b), for simplicity we used a single factor equivalent to the median at all sites.

References

Antonarakis, A. S., Munger, J. W., & Moorcroft, P. R. (2014, Jul). Imaging spectroscopy- and lidar-derived estimates of canopy composition and structure to improve predictions of forest carbon fluxes and ecosystem dynamics. *Geophys. Res. Lett.*, *41*(7), 2535–2542. doi: 10.1002/2013GL058373

Bahar, N. H. A., Ishida, F. Y., Weerasinghe, L. K., Guerrieri, R., O'Sullivan, O. S., Bloomfield, K. J., ... Atkin, O. K. (2017, May). Leaf-level photosynthetic capacity in lowland Amazonian and high-elevation Andean tropical moist forests of Peru. *New Phytol.*, *214*(3), 1002–1018. doi: 10.1111/nph.14079

Balch, J. K., Nepstad, D. C., Brando, P. M., Curran, L. M., Portela, O., de Carvalho, O., & Lefebvre, P. (2008, Oct). Negative fire feedback in a transitional forest of southeastern Amazonia. *Glob. Change Biol.*, *14*(10), 2276–2287. doi: 10.1111/j.1365-2486.2008.01655.x

Baraloto, C., Paine, C. E. T., Poorter, L., Beauchene, J., Bonal, D., Domenach, A.-M., ... Chave, J. (2010, Nov). Decoupled leaf and stem economics in rain forest trees. *Ecol. Lett.*, *13*(11), 1338–1347. doi: 10.1111/j.1461-0248.2010.01517.x

Blair, J. B., & Hofton, M. A. (1999, Aug). Modeling laser altimeter return waveforms over complex vegetation using high-resolution elevation data. *Geophys. Res. Lett.*, *26*(16), 2509–2512. doi: 10.1029/1999GL010484

Bonal, D., Bosc, A., Ponton, S., Goret, J.-Y., Burban, B., Gross, P., ... Granier, A. (2008, Aug). Impact of severe dry season on net ecosystem exchange in the Neotropical rainforest of French Guiana. *Glob. Change Biol.*, *14*(8), 1917–1933. doi: 10.1111/j.1365-2486.2008.01610.x

Brando, P. M., Balch, J. K., Nepstad, D. C., Morton, D. C., Putz, F. E., Coe, M. T., ... Soares-Filho, B. S. (2014, Apr). Abrupt increases in Amazonian tree mortality due to drought–fire interactions. *Proc. Natl. Acad. Sci. U. S. A.*, *111*(17), 6347–6352. doi: 10.1073/pnas.1305499111

Brando, P. M., Nepstad, D. C., Balch, J. K., Bolker, B., Christman, M. C., Coe, M., & Putz, F. E. (2012, Feb). Fire-induced tree mortality in a neotropical forest: the roles of bark traits, tree size, wood density and fire behavior. *Glob. Change Biol.*, *18*(2), 630–641. doi: 10.1111/j.1365-2486.2011.02533.x

Brando, P. M., Silvério, D., Maracahipes-Santos, L., Oliveira-Santos, C., Levick, S. R., Coe, M. T., ... Trumbore, S. E. (2019, Sep). Prolonged tropical forest degradation due to compounding disturbances: implications for CO_2 and H_2O fluxes of an experimental forest. *Glob. Change Biol.*, *25*(9), 2855–2868. doi: 10.1111/gcb.14659

Chave, J., Coomes, D., Jansen, S., Lewis, S. L., Swenson, N. G., & Zanne, A. E. (2009, Apr). Towards a worldwide wood economics spectrum. *Ecol. Lett.*, *12*(4), 351–366. doi: 10.1111/j.1461-0248.2009.01285.x

Chave, J., Réjou-Méchain, M., Búrquez, A., Chidumayo, E., Colgan, M. S., Delitti,

W. B., … Vieilledent, G. (2014, Oct). Improved allometric models to estimate the aboveground biomass of tropical trees. *Glob. Change Biol.*, *20*(10), 3177–3190. doi: 10.1111/gcb.12629

dos-Santos, M., Keller, M., & Morton, D. (2019, Dec). *LiDAR surveys over selected forest research sites, Brazilian Amazon, 2008–2018.* Retrieved 31 Jan 2020, from https://daac.ornl.gov/cgi-bin/dsviewer.pl?ds_id=1644 doi: 10.3334/ORNLDAAC/1644

Falster, D. S., Duursma, R. A., Ishihara, M. I., Barneche, D. R., FitzJohn, R. G., Vårhammar, A., … York, R. A. (2015, May). BAAD: a biomass and allometry database for woody plants. *Ecology*, *96*(5), 1445–1445. doi: 10.1890/14-1889.1

Falster, D. S., FitzJohn, R. G., Brännström, Å., Dieckmann, U., & Westoby, M. (2016, Feb). `plant`: A package for modelling forest trait ecology and evolution. *Methods Ecol. Evol.*, *7*(2), 136–146. doi: 10.1111/2041-210X.12525

Gourlet-Fleury, S., Ferry, B., Molino, J.-F., Petronelli, P., & Schmitt, L. (2004). Experimental plots: Key features. In S. Gourlet-Fleury, J.-M. Guehl, & O. Laroussinie (Eds.), *Ecology and management of a Neotropical rainforest: Lessons drawn from Paracou, a long-term experimental research site in French Guiana* (pp. 3–60). Paris: Elsevier.

Gourlet-Fleury, S., Guehl, J.-M., & Laroussinie, O. (2004). *Ecology and management of a Neotropical rainforest: Lessons drawn from Paracou, a long-term experimental research site in French Guiana.* Paris: Elsevier.

Gu, L., Norby, R., Haworth, I., Jensen, A., Turner, B., Walker, A., … Winter, K. (2016). *Photosynthetic parameters and nutrient content of trees at the Panama crane*

sites. 1.0. NGEE Tropics data collection. Retrieved 12 Sep 2019, from https://ngt-data.lbl.gov doi: 10.15486/NGT/1255260

Hancock, S., Armston, J., Hofton, M., Sun, X., Tang, H., Duncanson, L. I., … Dubayah, R. (2019, Feb). The GEDI simulator: A large-footprint waveform lidar simulator for calibration and validation of spaceborne missions. *Earth Space Sci.*, *6*(2), 290–310. doi: 10.1029/2018EA000506

Hayek, M. N., Longo, M., Wu, J., Smith, M. N., Restrepo-Coupe, N., Tapajós, R., … Wofsy, S. C. (2018, Aug). Carbon exchange in an Amazon forest: from hours to years. *Biogeosciences*, *15*(15), 4833–4848. doi: 10.5194/bg-15-4833-2018

Hunter, M. O., Keller, M., Vitoria, D., & Morton, D. C. (2013, Dec). Tree height and tropical forest biomass estimation. *Biogeosciences*, *10*(6), 10491–10529. doi: 10.5194/bg-10-8385-2013

Jucker, T., Caspersen, J., Chave, J., Antin, C., Barbier, N., Bongers, F., … Coomes, D. A. (2017, Jan). Allometric equations for integrating remote sensing imagery into forest monitoring programmes. *Glob. Change Biol.*, *23*(1), 177–190. doi: 10.1111/gcb.13388

Kattge, J., Bönisch, G., Díaz, S., Lavorel, S., Prentice, I. C., Leadley, P., … Wirth, C. (2020, Jan). TRY plant trait database — enhanced coverage and open access. *Glob. Change Biol.*, *26*(1), 119–188. doi: 10.1111/gcb.14904

Kattge, J., Díaz, S., Lavorel, S., Prentice, I. C., Leadley, P., Bönisch, G., … Wirth, C. (2011, Sep). TRY – a global database of plant traits. *Glob. Change Biol.*, *17*(9), 2905–2935. doi: 10.1111/j.1365-2486.2011.02451.x

Kattge, J., Knorr, W., Raddatz, T., & Wirth, C. (2009, Apr). Quantifying pho-

tosynthetic capacity and its relationship to leaf nitrogen content for global-scale terrestrial biosphere models. *Glob. Change Biol.*, *15*(4), 976–991. doi: 10.1111/j.1365-2486.2008.01744.x

Lees, J. M. (2017). RSEIS: Seismic time series analysis tools [Computer software manual]. Retrieved from https://CRAN.R-project.org/package=RSEIS (R package version 3.7-4)

Lei, Y., Treuhaft, R., Keller, M., dos-Santos, M., Gonçalves, F., & Neumann, M. (2018, Jun). Quantification of selective logging in tropical forest with spaceborne SAR interferometry. *Remote Sens. Environ.*, *211*, 167–183. doi: 10.1016/j.rse.2018.04.009

Leitold, V., Keller, M., Morton, D., Cook, B., & Shimabukuro, Y. (2015, Feb). Airborne lidar-based estimates of tropical forest structure in complex terrain: opportunities and trade-offs for REDD+. *Carbon Balance Manage.*, *10*(1), 3. doi: 10.1186/s13021-015-0013-x

Leitold, V., Morton, D. C., Longo, M., dos-Santos, M. N., Keller, M., & Scaranello, M. (2018, Aug). El Niño drought increased canopy turnover in Amazon forests. *New Phytol.*, *219*(3), 959–971. doi: 10.1111/nph.15110

Lombardozzi, D. L., Smith, N. G., Cheng, S. J., Dukes, J. S., Sharkey, T. D., Rogers, A., ... Bonan, G. B. (2018, Jul). Triose phosphate limitation in photosynthesis models reduces leaf photosynthesis and global terrestrial carbon storage. *Environ. Res. Lett.*, *13*(7), 074025. doi: 10.1088/1748-9326/aacf68

Longo, M., Keller, M., dos Santos, M. N., Leitold, V., Pinagé, E. R., Baccini, A., ... Morton, D. C. (2016, Nov). Aboveground biomass variability across intact and degraded forests in the Brazilian Amazon. *Global Biogeochem. Cycles*, *30*(11), 1639–

1660. doi: 10.1002/2016GB005465

Longo, M., Knox, R. G., Levine, N. M., Swann, A. L. S., Medvigy, D. M., Dietze, M. C., ... Moorcroft, P. R. (2019, Oct). The biophysics, ecology, and biogeochemistry of functionally diverse, vertically and horizontally heterogeneous ecosystems: the Ecosystem Demography model, version 2.2 – part 2: Model evaluation for tropical South America. *Geosci. Model Dev.*, *12*(10), 4347–4374. doi: 10.5194/gmd-12-4347-2019

Longo, M., Knox, R. G., Medvigy, D. M., Levine, N. M., Dietze, M. C., Kim, Y., ... Moorcroft, P. R. (2019, Oct). The biophysics, ecology, and biogeochemistry of functionally diverse, vertically and horizontally heterogeneous ecosystems: the Ecosystem Demography model, version 2.2 – part 1: Model description. *Geosci. Model Dev.*, *12*(10), 4309–4346. doi: 10.5194/gmd-12-4309-2019

MacArthur, R. H., & Horn, H. S. (1969, Sep). Foliage profile by vertical measurements. *Ecology*, *50*(5), 802–804. doi: 10.2307/1933693

Mascaro, J., Litton, C. M., Hughes, R. F., Uowolo, A., & Schnitzer, S. A. (2011, Nov). Minimizing bias in biomass allometry: Model selection and log-transformation of data. *Biotropica*, *43*(6), 649–653. doi: 10.1111/j.1744-7429.2011.00798.x

Miller, A. J. (1984, Nov). Selection of subsets of regression variables. *J. R. Stat. Soc. A-Gen.*, *147*(3), 389–425. doi: 10.2307/2981576

Moorcroft, P. R., Hurtt, G. C., & Pacala, S. W. (2001, Nov). A method for scaling vegetation dynamics: The Ecosystem Demography model (ED). *Ecol. Monogr.*, *71*(4), 557–586. doi: 10.1890/0012-9615(2001)071{$[$}0557:AMFSVD{$]$}2.0.CO;2

Morton, D. C., Le Page, Y., DeFries, R. S., Collatz, G. J., & Hurtt, G. C. (2013, Jun).

Understorey fire frequency and the fate of burned forests in southern Amazonia. *Philos. Trans. R. Soc. B-Biol. Sci.*, *368*(1619), 20120163. doi: 10.1098/rstb.2012.0163

Ni-Meister, W., Jupp, D., & Dubayah, R. (2001, Sep). Modeling lidar waveforms in heterogeneous and discrete canopies. *IEEE T. Geosci. Remote Sens.*, *39*(9), 1943–1958. doi: 10.1109/36.951085

Norby, R. J., Gu, L., Haworth, I. C., Jensen, A. M., Turner, B. L., Walker, A. P., ... Winter, K. (2017, Sep). Informing models through empirical relationships between foliar phosphorus, nitrogen and photosynthesis across diverse woody species in tropical forests of Panama. *New Phytol.*, *215*(4), 1425–1437. doi: 10.1111/nph.14319

Oleson, K. W., Lawrence, D. M., Bonan, G. B., Drewniak, B., Huang, M., Koven, C. D., ... Yang, Z.-L. (2013). *Technical description of version 4.5 of the community land model (CLM)* (Technical Report Nos. NCAR/TN-503+STR). Boulder, CO: NCAR. (420pp.) doi: 10.5065/D6RR1W7M

Pinagé, E. R., Keller, M., Duffy, P., Longo, M., dos Santos, M. N., & Morton, D. C. (2019, Mar). Long-term impacts of selective logging on Amazon forest dynamics from multi-temporal airborne LiDAR. *Remote Sens.*, *11*(6), 709. doi: 10.3390/rs11060709

Pinto, A., Amaral, P., Souza Jr., C. M., Veríssimo, A., Salomão, R., Gomes, G., & Balieiro, C. (2009). *Diagnóstico socioeconômico e florestal do município de Paragominas* (Technical Report). Belém, PA, Brazil: Instituto do Homem e Meio Ambiente da Amazônia (Imazon). (Available at `http://imazon.org.br/publicacoes/diagnostico-socioeconomico-e-florestal-do-municipio-de-paragominas/`)

Popescu, S. C., Zhao, K., Neuenschwander, A., & Lin, C. (2011, Nov). Satellite lidar

vs. small footprint airborne lidar: Comparing the accuracy of aboveground biomass estimates and forest structure metrics at footprint level. *Remote Sens. Environ.*, *115*(11), 2786–2797. doi: 10.1016/j.rse.2011.01.026

Powers, J. S., & Tiffin, P. (2010, Aug). Plant functional type classifications in tropical dry forests in Costa Rica: leaf habit versus taxonomic approaches. *Funct. Ecol.*, *24*(4), 927–936. doi: 10.1111/j.1365-2435.2010.01701.x

Pyle, E. H., Santoni, G. W., Nascimento, H. E. M., Hutyra, L. R., Vieira, S., Curran, D. J., ... Wofsy, S. C. (2008, Mar). Dynamics of carbon, biomass, and structure in two Amazonian forests. *J. Geophys. Res.-Biogeosci.*, *113*(G1), G00B08. doi: 10.1029/2007JG000592

Rappaport, D., Morton, D., Longo, M., Keller, M., Dubayah, R., & dos-Santos, M. N. (2018, Jun). Quantifying long-term changes in carbon stocks and forest structure from Amazon forest degradation. *Environ. Res. Lett.*, *13*(6), 065013. doi: 10.1088/1748-9326/aac331

Santiago, L. S., & Wright, S. J. (2007, Feb). Leaf functional traits of tropical forest plants in relation to growth form. *Funct. Ecol.*, *21*(1), 19–27. doi: 10.1111/j.1365-2435.2006.01218.x

Schwarz, G. (1978, Mar). Estimating the dimension of a model. *Ann. Stat.*, *6*(2), 461–464. doi: 10.1214/aos/1176344136

Shao, G., Stark, S. C., de Almeida, D. R., & Smith, M. N. (2019, Feb). Towards high throughput assessment of canopy dynamics: The estimation of leaf area structure in Amazonian forests with multitemporal multi-sensor airborne lidar. *Remote Sens. Environ.*, *221*(221), 1–13. doi: 10.1016/j.rse.2018.10.035

Stark, S. C., Leitold, V., Wu, J. L., Hunter, M. O., de Castilho, C. V., Costa, F. R. C., … Saleska, S. R. (2012, Dec). Amazon forest carbon dynamics predicted by profiles of canopy leaf area and light environment. *Ecol. Lett.*, *15*(12), 1406–1414. doi: 10.1111/j.1461-0248.2012.01864.x

Sustainable Landscapes Brazil. (2019, Nov). Retrieved 9 Jan 2019, from `https://www.paisagenslidar.cnptia.embrapa.br/webgis/`

Tang, H., & Dubayah, R. (2017, Mar). Light-driven growth in Amazon evergreen forests explained by seasonal variations of vertical canopy structure. *Proc. Natl. Acad. Sci. U. S. A.*, *114*(10), 2640–2644. doi: 10.1073/pnas.1616943114

Veríssimo, A., Barreto, P., Mattos, M., Tarifa, R., & Uhl, C. (1992, Dec). Logging impacts and prospects for sustainable forest management in an old Amazonian frontier: The case of Paragominas. *Forest Ecol. Manag.*, *55*(1–4), 169–199. doi: 10.1016/0378-1127(92)90099-U

Vincent, G., Antin, C., Laurans, M., Heurtebize, J., Durrieu, S., Lavalley, C., & Dauzat, J. (2017, Sep). Mapping plant area index of tropical evergreen forest by airborne laser scanning. a cross-validation study using LAI2200 optical sensor. *Remote Sens. Environ.*, *198*, 254–266. doi: 10.1016/j.rse.2017.05.034

von Caemmerer, S. (2000). *Biochemical models of leaf photosynthesis* (No. 2). Collingwood, VIC, Australia: CSIRO Publishing. doi: 10.1006/anbo.2000.1296

Wright, I. J., Reich, P. B., Westoby, M., Ackerly, D. D., Baruch, Z., Bongers, F., … Villar, R. (2004, Apr). The worldwide leaf economics spectrum. *Nature*, *428*(6985), 821–827. doi: 10.1038/nature02403

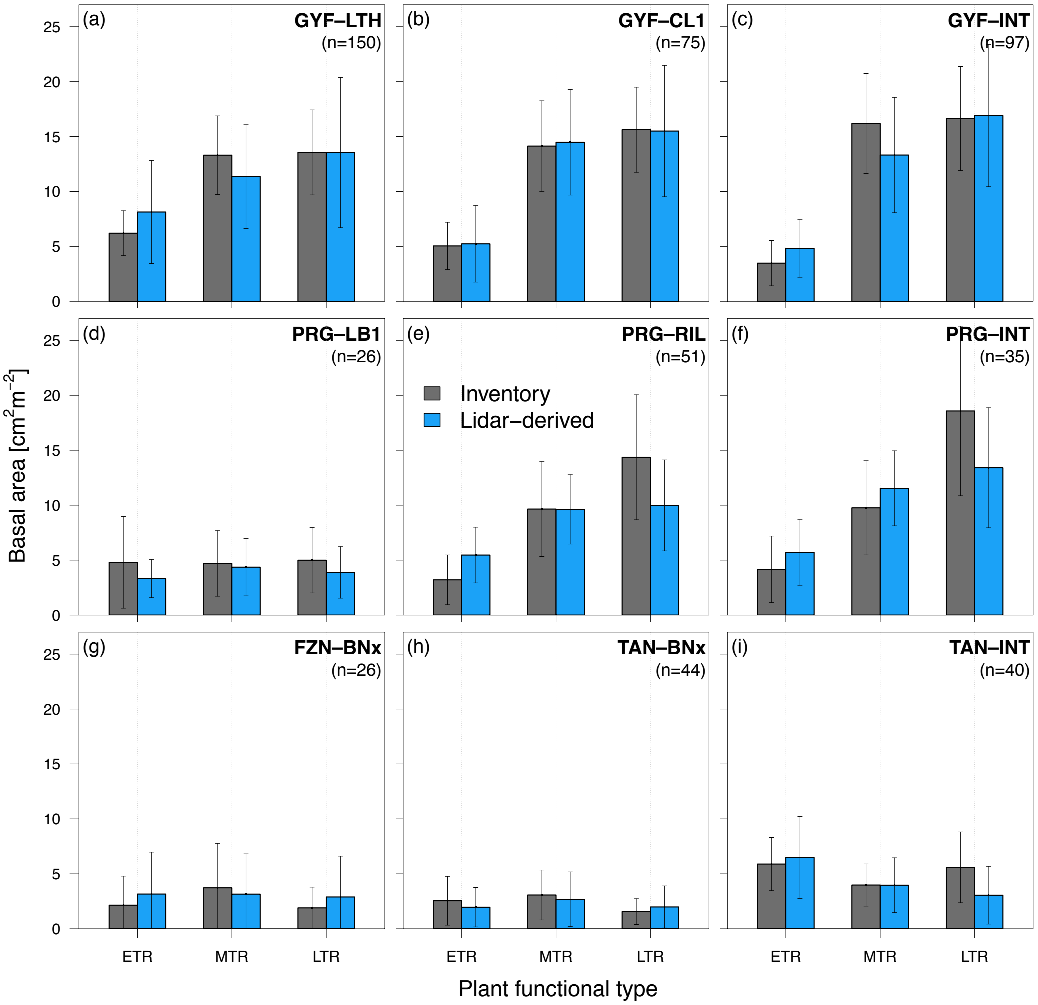

Figure S1. Assessment of basal area by plant functional types (PFTs), for different study regions and degradation levels. Plant functional types are early-successional tropical tree (ETR), mid-successional tropical tree (MTR) and late-successional tropical tree (LTR). Grey bars are obtained from forest inventory plots, and blue bars are obtained from the airborne lidar initialization using a 612-fold regional cross-validation (i.e. excluding all plots from region in the calibration stage). Whiskers correspond to the standard deviation either across all plots in the same category (inventory) or across all plots and replicates (lidar). Sites: GYF – Paracou, PRG – Paragominas, FZN – Feliz Natal, TAN – Tanguro. Disturbance classes: BNx – Burned twice or more, CL1 – conventional logging (once), LB1 – logged and burned once, LTH – logged and thinned, RIL – reduced-impact logging, INT – intact.

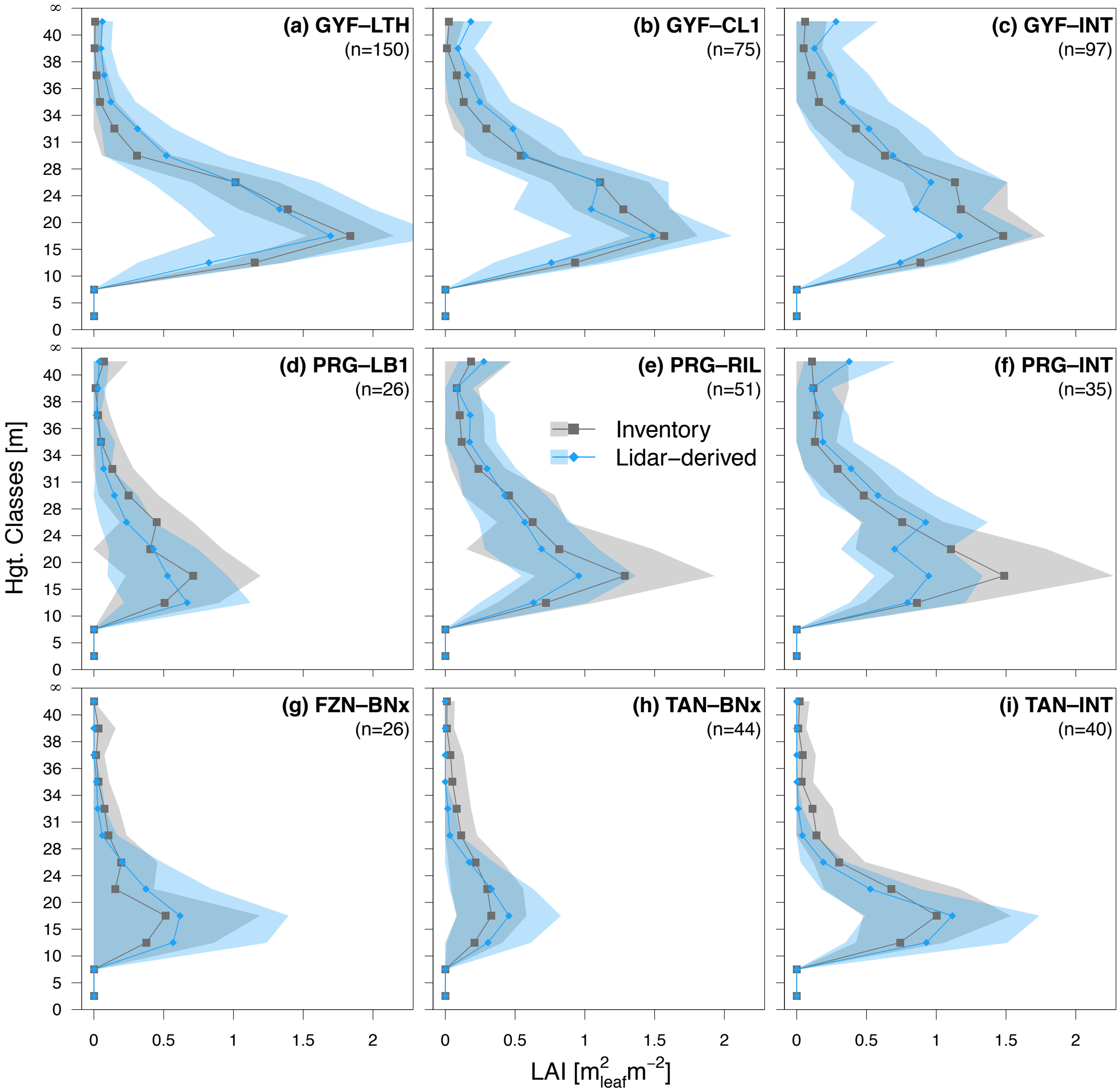

Figure S2. Assessment of leaf area index distribution as a function of height for different study regions and degradation levels. Grey points are obtained from forest inventory plots, and blue points are obtained from the airborne lidar initialization using a 612-fold regional cross-validation (i.e. excluding all plots from region in the calibration stage). Bands around points correspond to the standard deviation either across all plots in the same category (inventory) or across all plots and replicates (lidar). Sites: GYF – Paracou, PRG – Paragominas, FZN – Feliz Natal, TAN – Tanguro. Disturbance classes: BNx – Burned twice or more, CL1 – conventional logging (once), LB1 – logged and burned once, LTH – logged and thinned, RIL – reduced-impact logging, INT – intact.

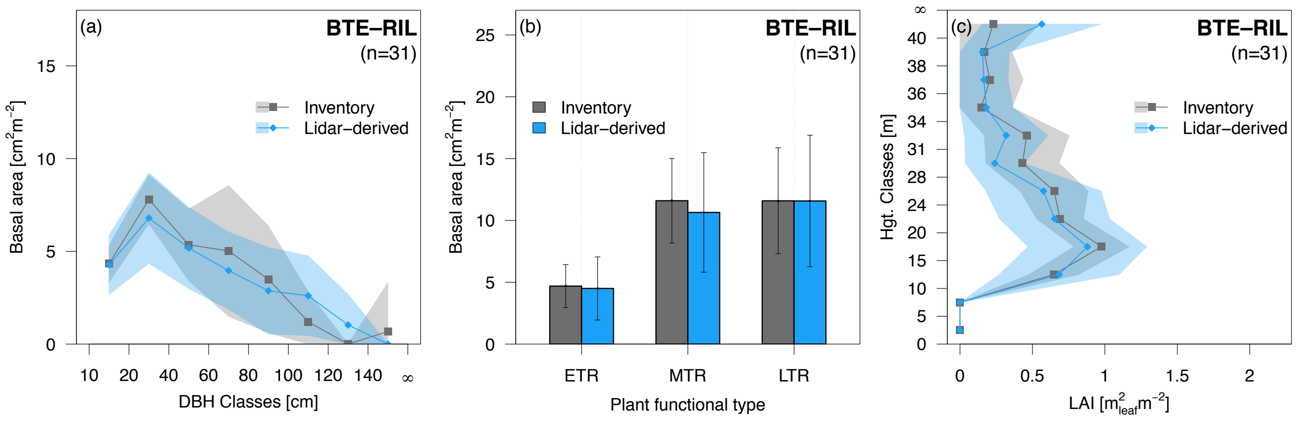

Figure S3. Assessment of airborne lidar initialization for Belterra (BTE). Comparison of (a) basal area distribution across diameter of breast height (DBH) classes, (b) basal area distribution among plant functional types (PFTs), and (c) leaf area index distribution as a function of height, for reduced-impact logging (RIL, the only disturbance type with $n > 20$ plots in BTE). Plant functional types are early-successional tropical tree (ETR), mid-successional tropical tree (MTR) and late-successional tropical tree (LTR). Grey points and bars are obtained from forest inventory plots, and blue points and bars are obtained from the airborne lidar initialization using a 612-fold regional cross-validation (i.e. excluding all plots from region in the calibration stage). Bands around points and whiskers correspond to the standard deviation either across all plots in the same category (inventory) or across all plots and replicates (lidar).

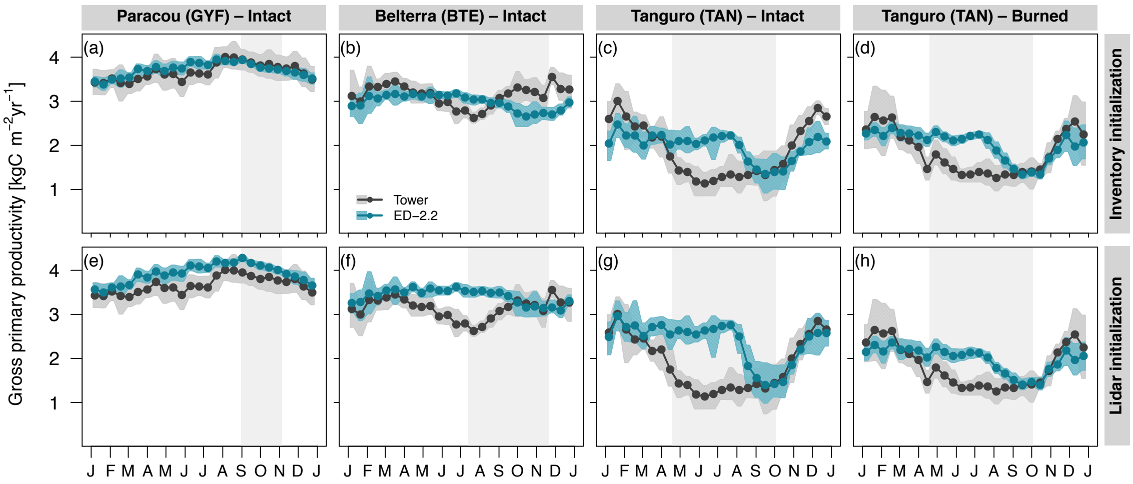

Figure S4. Model assessment of gross primary productivity. Fortnightly averages of gross primary productivity at (a,e) Paracou (GYF), intact forest; (b,f) Belterra (BTE), intact forests; (c,g) Tanguro (TAN), intact forests; (d,h) Tanguro (TAN), burned forests, initialized with (a-d) forest inventory plots and (e-h) airborne lidar. Fortnightly averages for both ED-2.2 and tower estimates were calculated using only hours with available data from the tower, and were integrated by obtaining the mean diurnal cycle then averaging the mean diurnal cycle to avoid biases due to data gaps. Bands around the averages correspond to the 95% confidence interval of the means, obtained through bootstrap. The grey rectangle in the background corresponds to the average dry season.

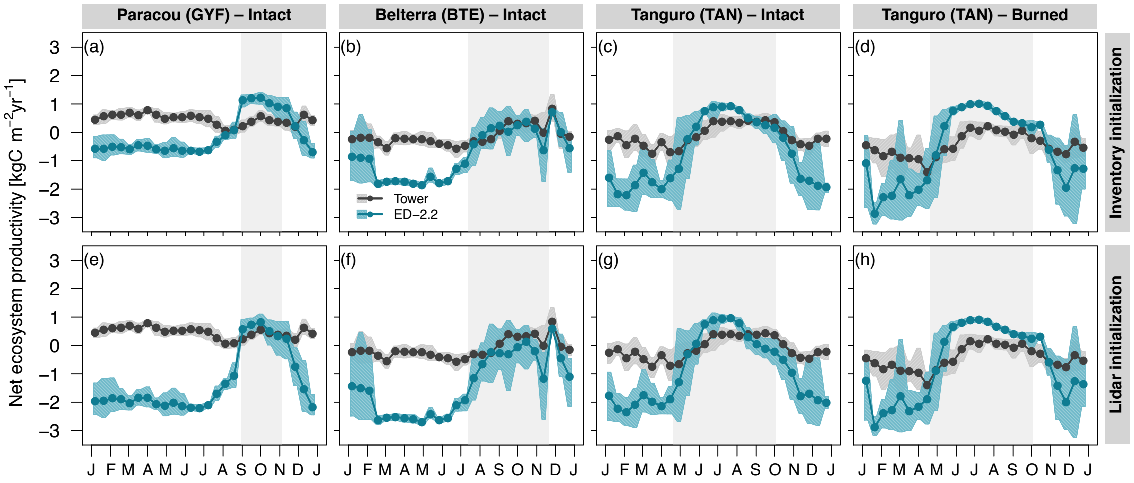

Figure S5. Model assessment of net ecosystem productivity. Fortnightly averages of net ecosystem productivity at (a,e) Paracou (GYF), intact forest; (b,f) Belterra (BTE), intact forests; (c,g) Tanguro (TAN), intact forests; (d,h) Tanguro (TAN), burned forests, initialized with (a-d) forest inventory plots and (e-h) airborne lidar. Positive fluxes mean net uptake. Fortnightly averages for both ED-2.2 and tower estimates were calculated using only hours with available data from the tower, and were integrated by obtaining the mean diurnal cycle then averaging the mean diurnal cycle to avoid biases due to data gaps. Bands around the averages correspond to the 95% confidence interval of the means, obtained through bootstrap. The grey rectangle in the background corresponds to the average dry season.

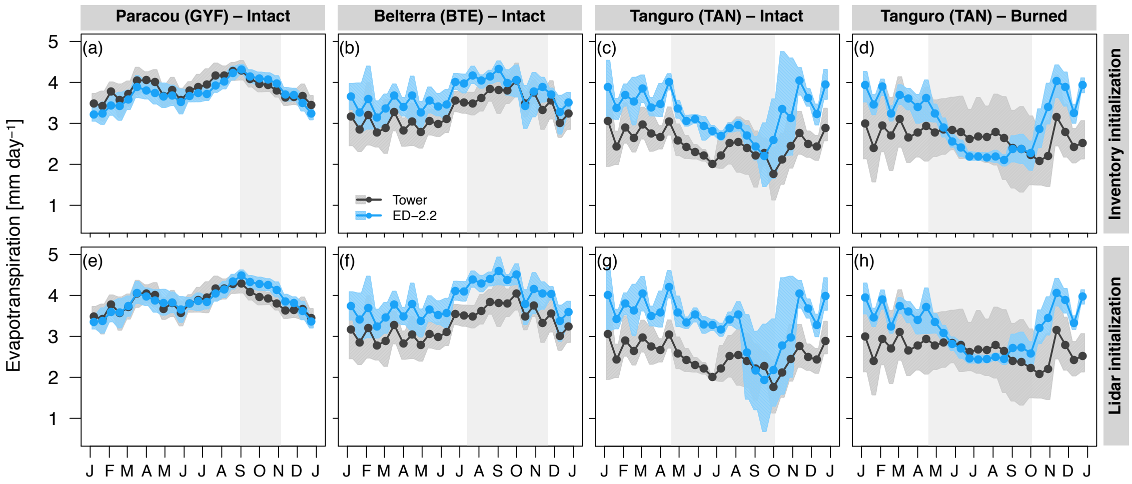

Figure S6. Model assessment of evapotranspiration. Fortnightly averages of water heat flux at (a,e) Paracou (GYF), intact forest; (b,f) Belterra (BTE), intact forests; (c,g) Tanguro (TAN), intact forests; (d,h) Tanguro (TAN), burned forests, initialized with (a-d) forest inventory plots and (e-h) airborne lidar. Fortnightly averages for both ED-2.2 estimates and tower measurements were calculated using only hours with available data from the tower, and were integrated by obtaining the mean diurnal cycle then averaging the mean diurnal cycle to avoid biases due to data gaps. Bands around the averages correspond to the 95% confidence interval of the means, obtained through bootstrap. The grey rectangle in the background corresponds to the average dry season.

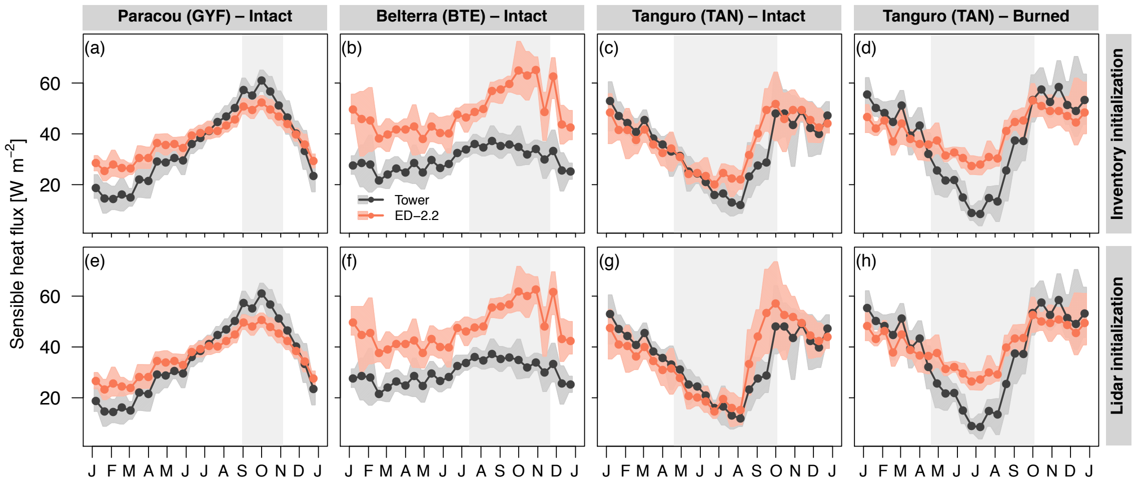

Figure S7. Model assessment of sensible heat flux. Fortnightly averages of sensible heat flux at (a,e) Paracou (GYF), intact forest; (b,f) Belterra (BTE), intact forests; (c,g) Tanguro (TAN), intact forests; (d,h) Tanguro (TAN), burned forests, initialized with (a-d) forest inventory plots and (e-h) airborne lidar. Fortnightly averages for both ED-2.2 estimates and tower measurements were calculated using only hours with available data from the tower, and were integrated by obtaining the mean diurnal cycle then averaging the mean diurnal cycle to avoid biases due to data gaps. Bands around the averages correspond to the 95% confidence interval of the means, obtained through bootstrap. The grey rectangle in the background corresponds to the average dry season.

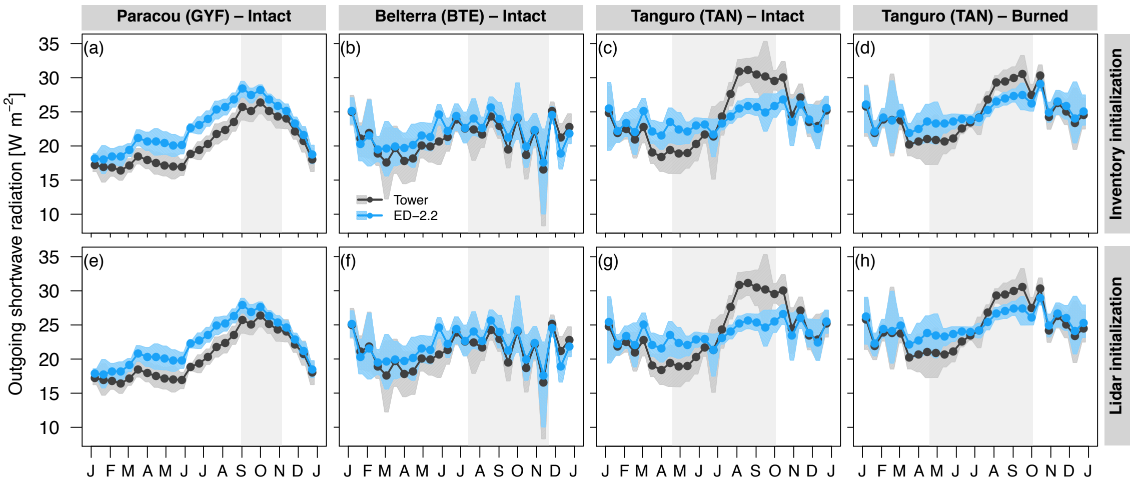

Figure S8. Model assessment of outgoing shortwave radiation. Fortnightly averages of outgoing shortwave radiation at (a,e) Paracou (GYF), intact forest; (b,f) Belterra (BTE), intact forests; (c,g) Tanguro (TAN), intact forests; (d,h) Tanguro (TAN), burned forests, initialized with (a-d) forest inventory plots and (e-h) airborne lidar. Fortnightly averages for both ED-2.2 estimates and tower measurements were calculated using only hours with available data from the tower, and were integrated by obtaining the mean diurnal cycle then averaging the mean diurnal cycle to avoid biases due to data gaps. Bands around the averages correspond to the 95% confidence interval of the means, obtained through bootstrap. The grey rectangle in the background corresponds to the average dry season.

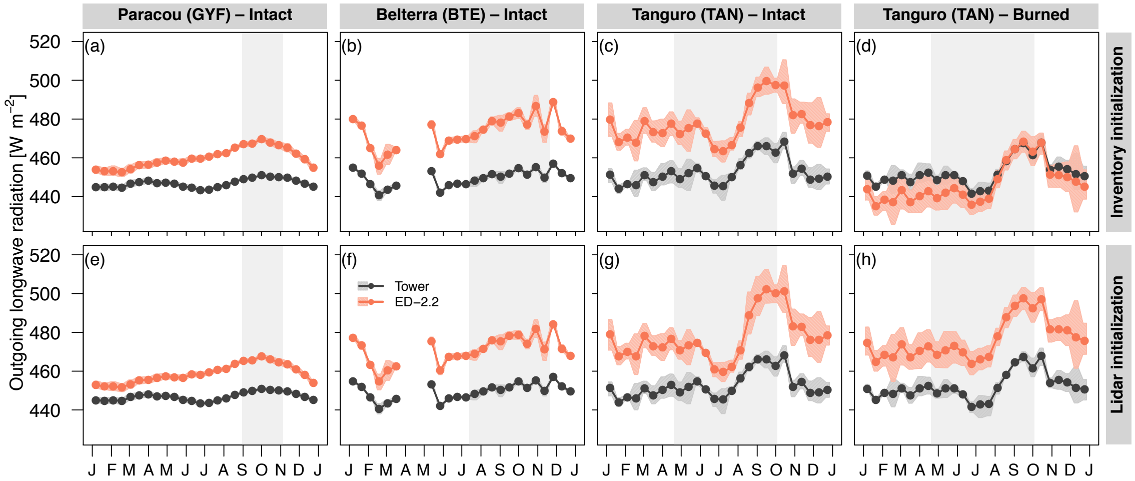

Figure S9. Model assessment of outgoing longwave radiation. Fortnightly averages of outgoing longwave radiation at (a,e) Paracou (GYF), intact forest; (b,f) Belterra (BTE), intact forests; (c,g) Tanguro (TAN), intact forests; (d,h) Tanguro (TAN), burned forests, initialized with (a-d) forest inventory plots and (e-h) airborne lidar. Fortnightly averages for both ED-2.2 estimates and tower measurements were calculated using only hours with available data from the tower, and were integrated by obtaining the mean diurnal cycle then averaging the mean diurnal cycle to avoid biases due to data gaps. Missing fortnightly periods at BTE did not have sufficient measurements to characterize the entire diurnal cycle. Bands around the averages correspond to the 95% confidence interval of the means, obtained through bootstrap. The grey rectangle in the background corresponds to the average dry season.

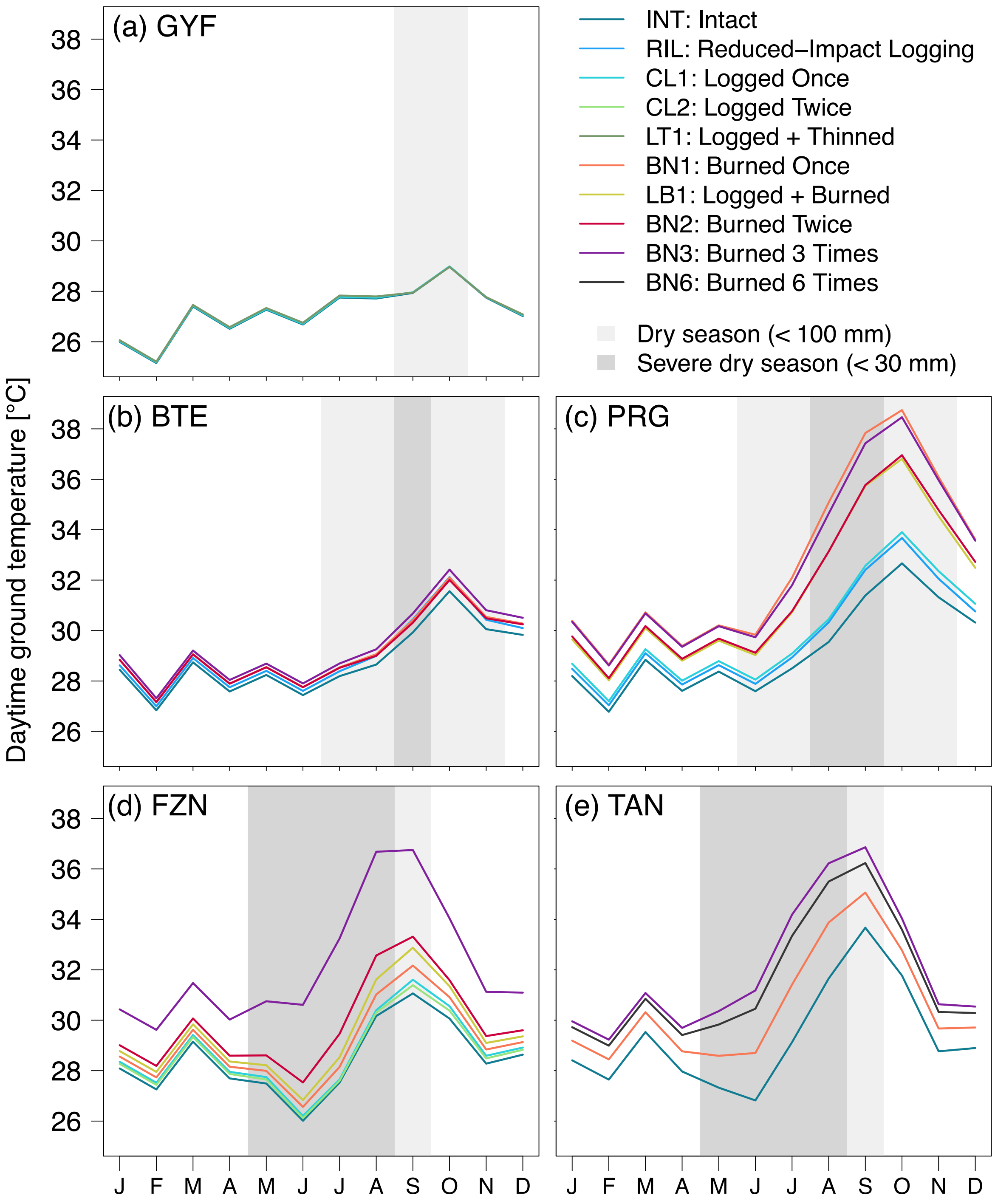

Figure S10. Multi-decadal average daytime ground temperate as a function of region and degradation. Monthly means of ground temperature (1980–2016), simulated by ED-2.2 and driven by MERRA-2 and MSWEP-2.2 for (a) Paracou (GYF), (b) Belterra (BTE), (c) Paragominas (PRG), (d) Feliz Natal (FZN), and (e) Tanguro (TAN), aggregated by degradation history (lines). Grey rectangles in the background correspond to the average dry season.

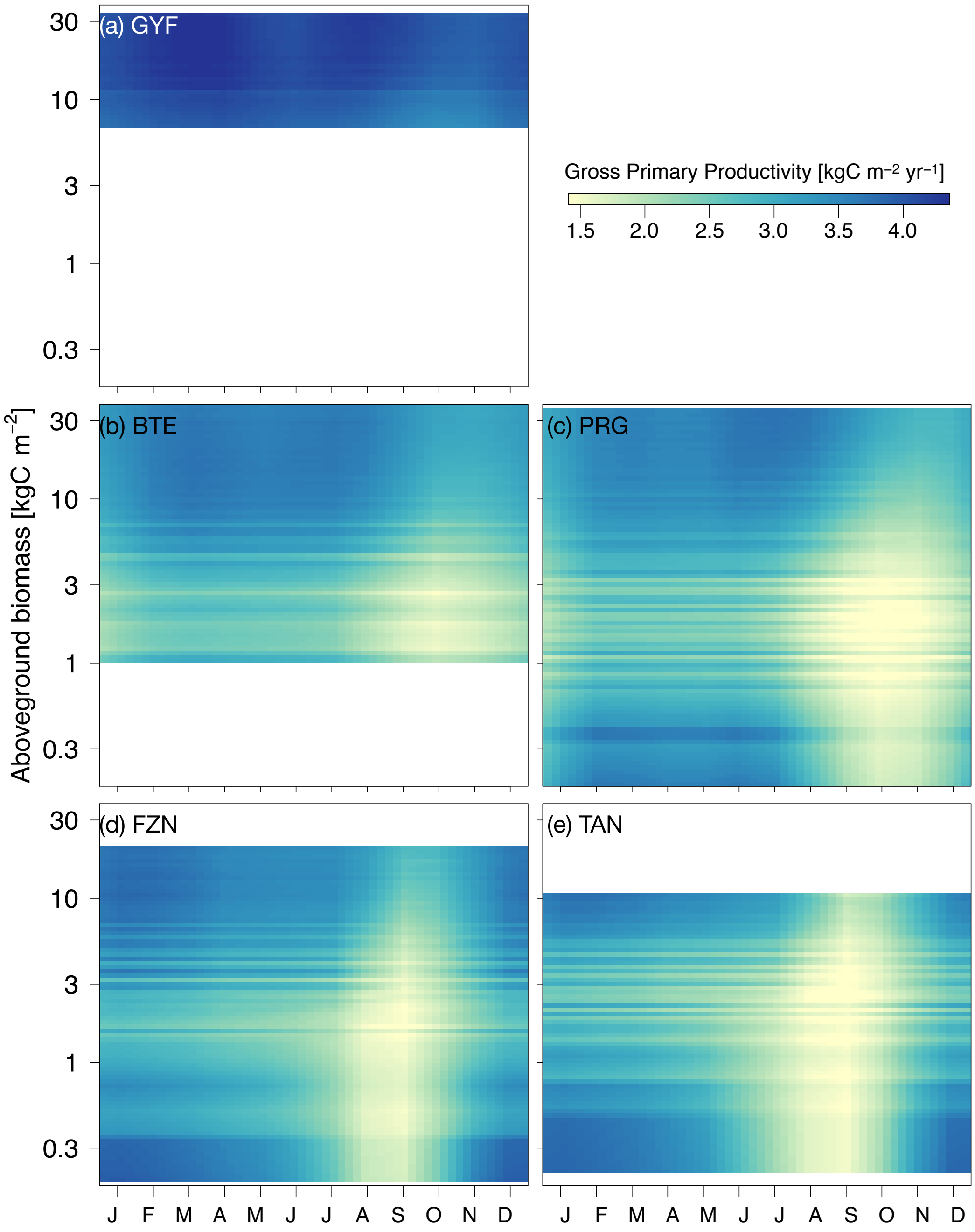

Figure S11. Monthly mean daytime gross primary productivity as a function of region and local (patch) aboveground biomass. Monthly averages correspond to the 1980–2016 period, simulated by ED-2.2 for (a) Paracou (GYF), (b) Belterra (BTE), (c) Paragominas (PRG), (d) Feliz Natal (FZN), and (e) Tanguro (TAN), and the y axis corresponds to the aboveground biomass for each patch, linearly interpolated for visualization. White areas are outside the range of biomass of each region and thus excluded.

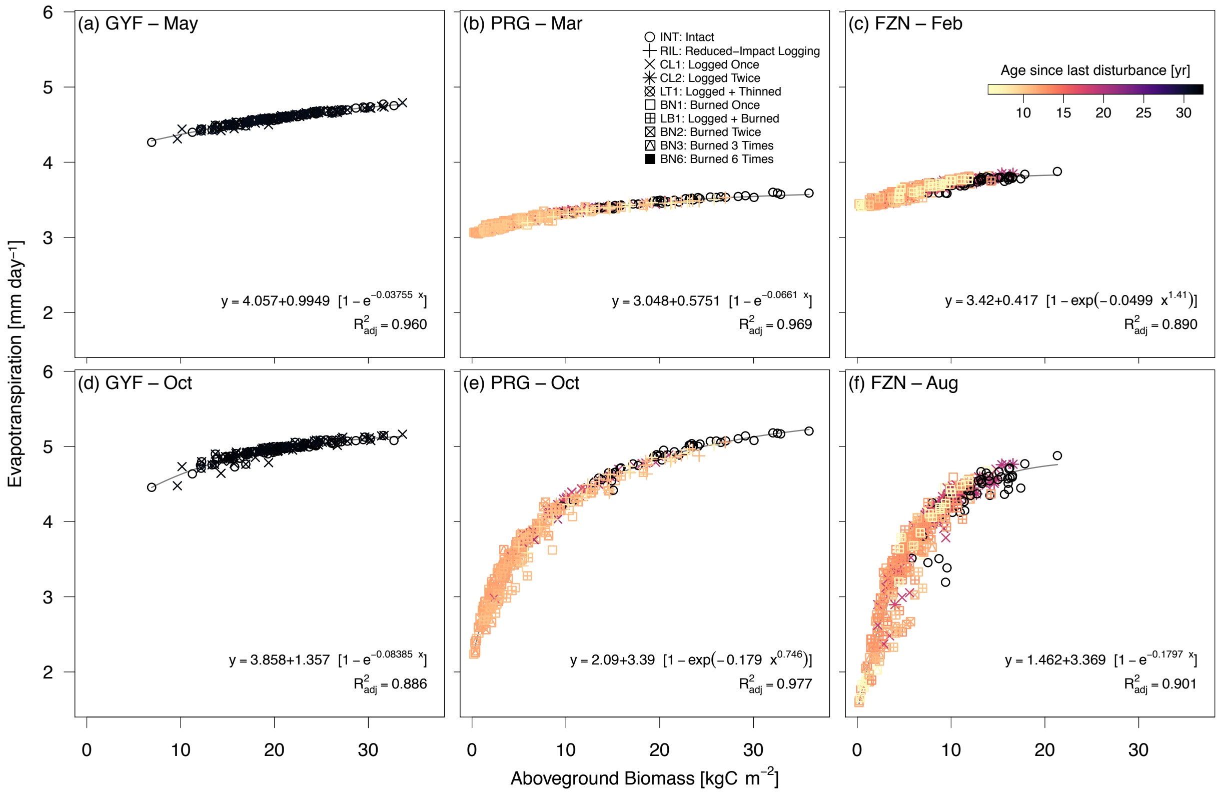

Figure S12. Variability of evapotranspiration (ET) as a function of local (patch) aboveground biomass (AGB). Scatter plot of AGB (x axis) and water flux (y axis) at sites (a,d) Paracou (GYF), (b,e) Paragominas (PRG), (c,f) Feliz Natal (FZN), for (a-c) the peak of wet season — May (GYF), March (PRG), and February (FZN) — and (d-f) peak of dry season — October (GYF and PRG), and August (FZN). Each point represents the 1980–2016 average ET of each patch solved by ED-2.2; point shapes correspond to the disturbance history, and point colors represent the time between the last disturbance (undetermined for intact forests) and lidar data acquisition. Curves correspond to non-linear least squares fits of the most parsimonious function, defined from Bayesian Information Criterion (Schwarz, 1978), between shifted exponential or shifted Weibull functions.

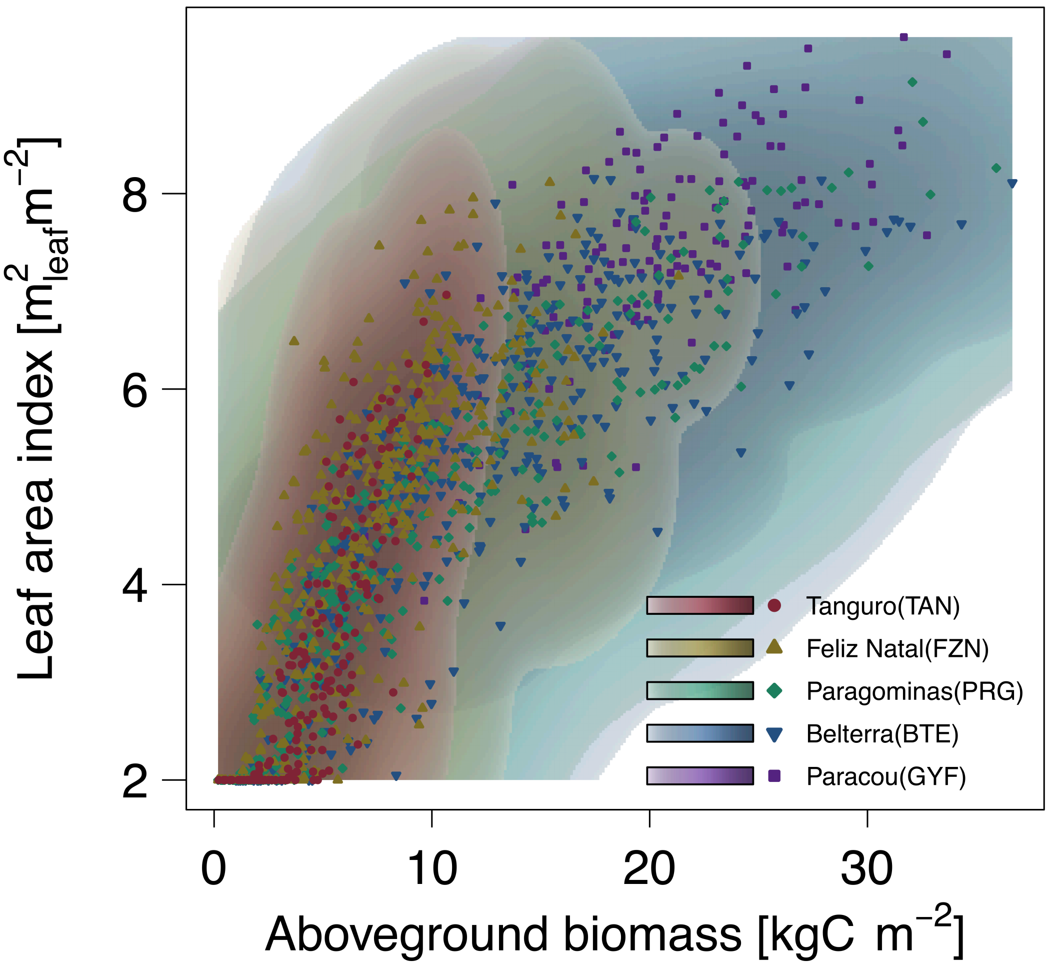

Figure S13. Leaf area index as a function of aboveground biomass. Scatter plot shows the leaf area index (x axis) and aboveground biomass (y axis) for each simulated patch across all regions. Density cloud (background color) was produced through a bi-dimensional kernel density estimator; points are the averages used to generate each density cloud. Color ramps (logarithmic) range from $0.1 - 100\%$ of the maximum computed scale.

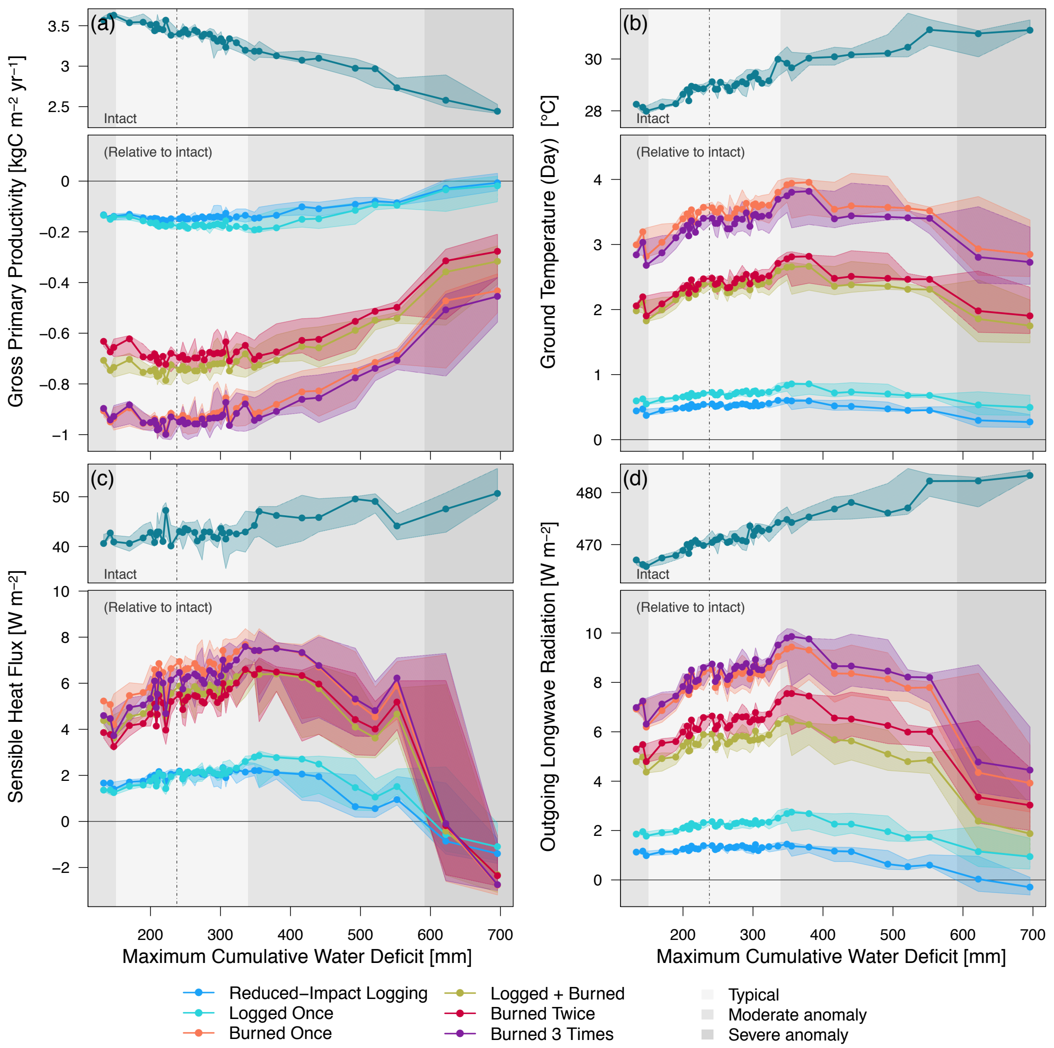

Figure S14. Response of the carbon and energy cycle components across a forest degradation gradient and drought severity in Paragominas (PRG). Selected components: (a) gross primary productivity, (b) daytime ground temperature, (c) sensible heat flux, and (d) outgoing longwave radiation. Points correspond to the median value of 12-month running averages, aggregated into 40 quantiles along the range of maximum cumulative water deficit (MCWD). Bands around the points correspond to the 95% range within each MCWD bin. Top panels are the absolute value for intact forests, and bottom panels are the absolute difference between degraded and intact forests. Background shades denote the MCWD anomaly: light grey – 68% range around the median (dot-dash vertical line); intermediate grey – 95% range; dark grey – anomalies exceeding the 95% range.

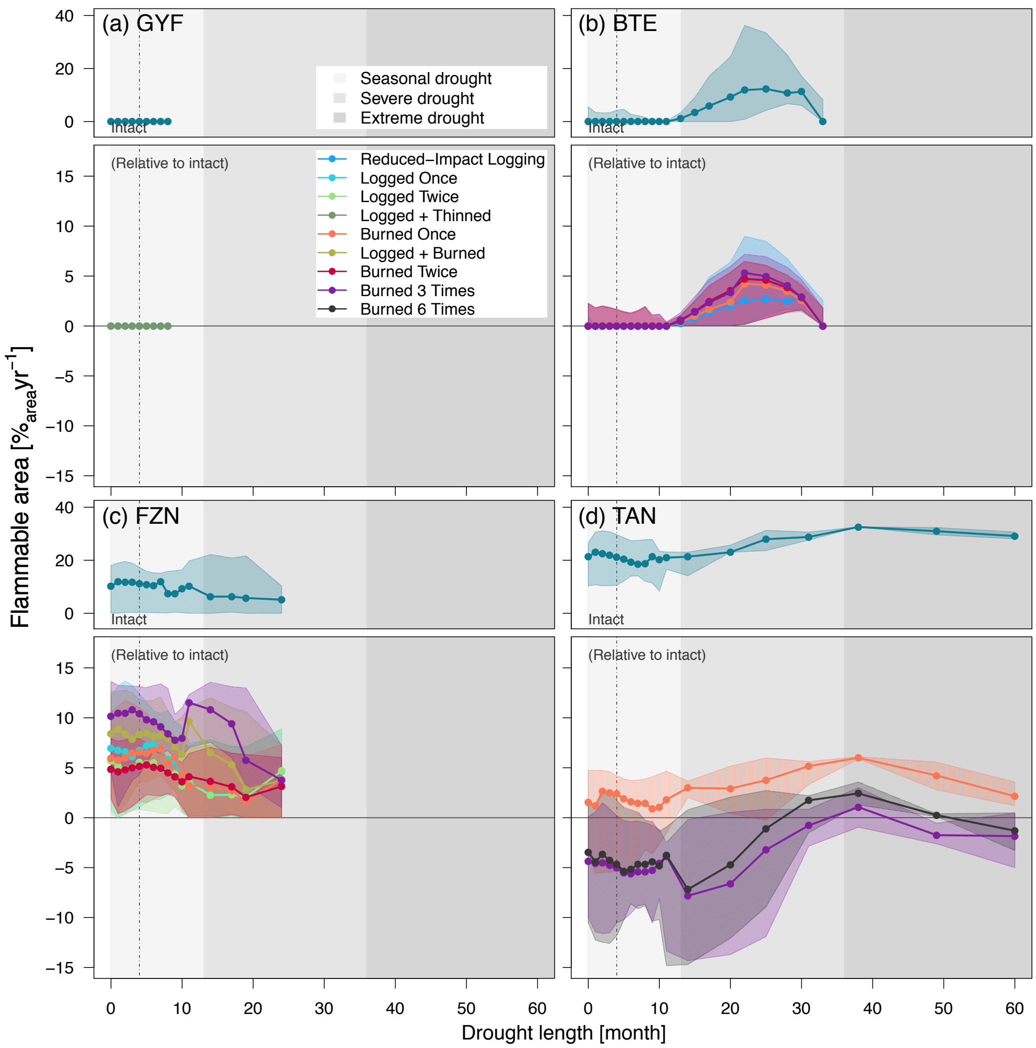

Figure S15. Flammable area as a function of degradation history and drought length (number of consecutive months with water deficit in excess of 20 mm) for regions (a) Paracou (GYF), (b) Belterra (BTE), (c) Feliz Natal (FZN), and (d) Tanguro (TAN). Points correspond to the median value of 12-month running averages, aggregated into quantiles along the drought length. Bands around the points correspond to the 95% range within each drought length bin. Top panels are the absolute value for intact forests, and bottom panels are the absolute difference between degraded and intact forests. Background shades denote drought-length classes used in the text: seasonal (light gray, less than 12 months); severe (intermediate gray, 12–36 months); extreme (dark grey; more than 36 months).

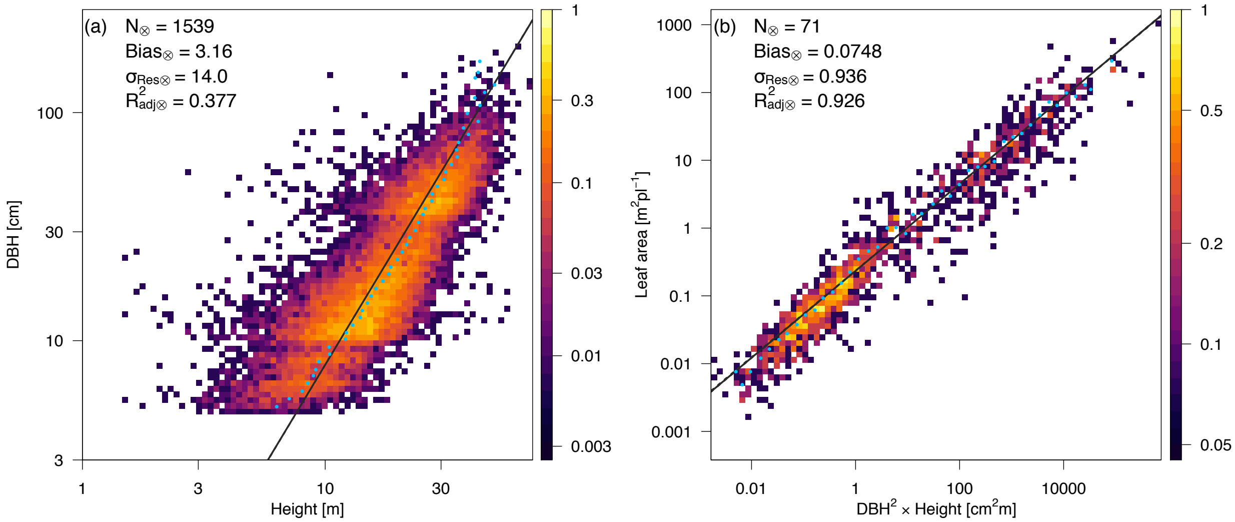

Figure S16. Fit of the allometric equations developed for the airborne lidar initialization and for ED-2.2 simulations. (a) Diameter at breast height (D) as a function of tree height (H); line corresponds to the standardized major axis equation defined by Equation (S1). (b) Individual leaf area (L) as a function of size ($D^2\,H$). Shaded background corresponds to the density of observed points. The results of the binned sampling with the lowest root mean square error are also shown: blue dots correspond to the binned sampled points used for the model fitting, black lines are the fitted model, and the goodness-of-fit metrics for the cross validation are shown for reference.

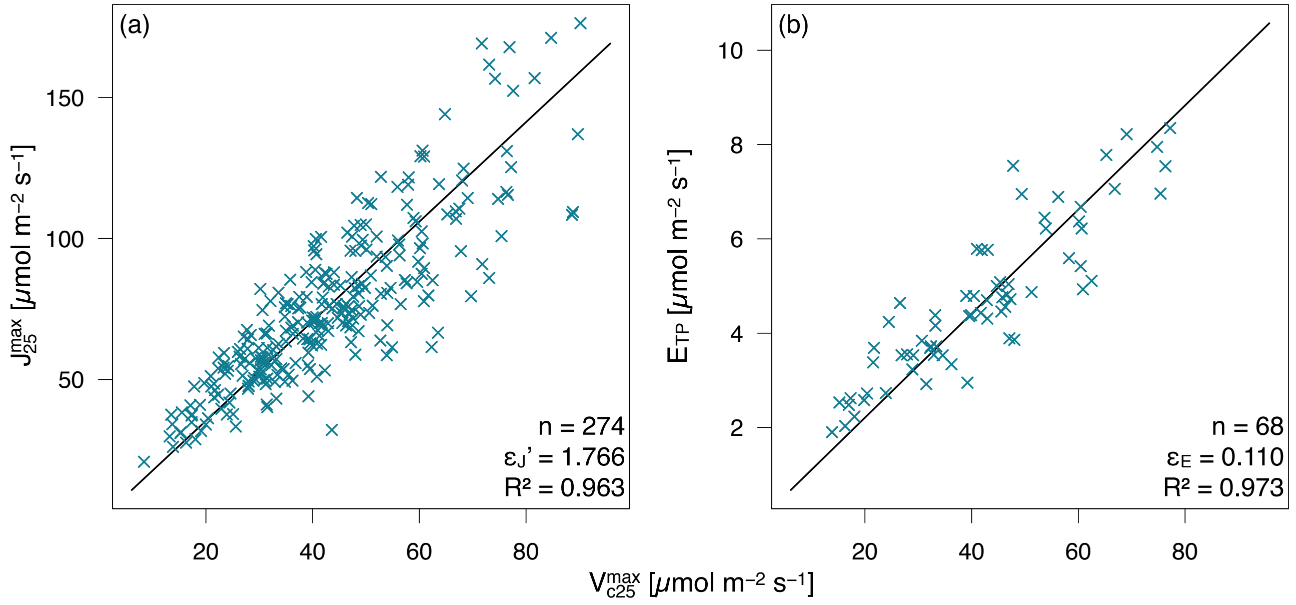

Figure S17. Scatter plots of (a) maximum electron transport rate at 25°C ($J_{25}^{\max}$) and (b) triose phosphate utilization rate (E^{TP}) as functions of maximum carboxylation rate at 25°C ($C_{c25}^{\max}$). Data were pooled from Gu et al. (2016). The slopes ε'_J and ε_E were obtained by fitting standardized major axes (SMA) and imposing zero intercept. The number of points (N), the slope of the SMA line (ε'_J and ε_E, respectively), and the R^2 for the SMA curve are also shown for reference.

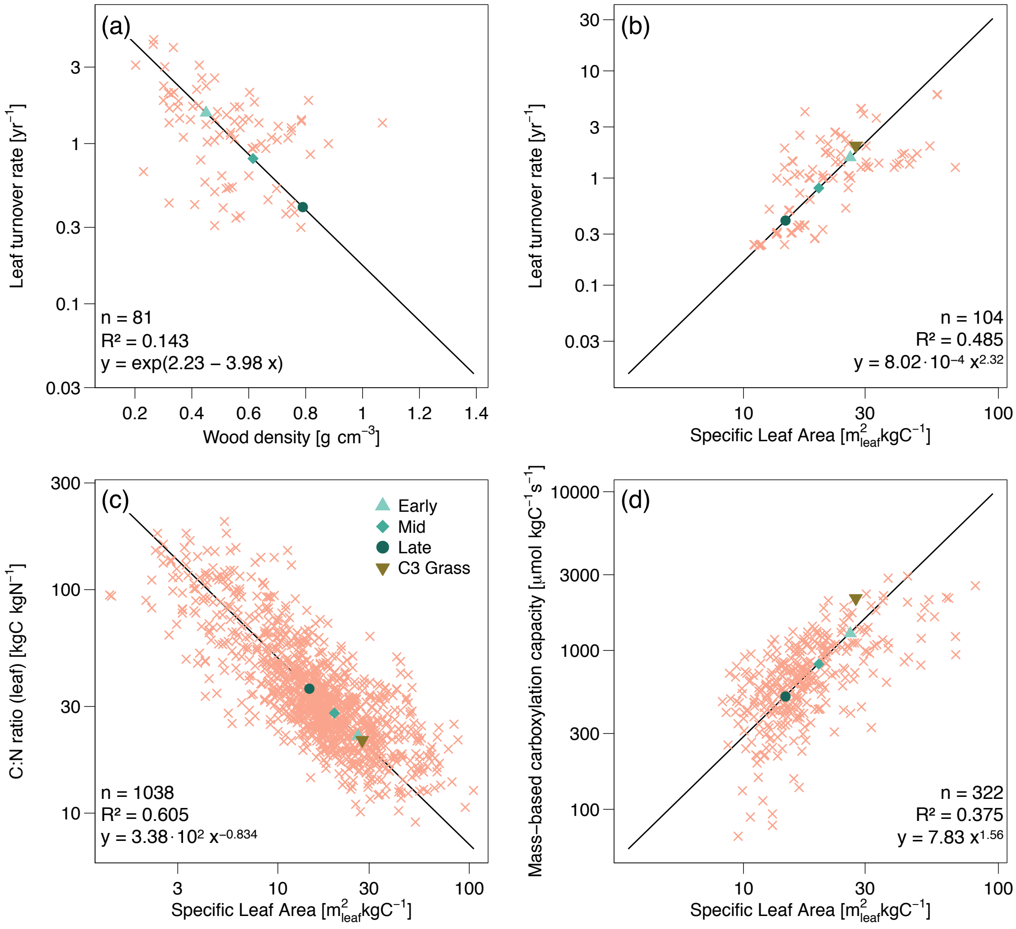

Figure S18. Scatter plots of trait relationships obtained from multiple studies and trait data bases, including GLOPNET and TRY (Wright et al., 2004; Santiago & Wright, 2007; Chave et al., 2009; Kattge et al., 2009, 2011; Baraloto et al., 2010; Powers & Tiffin, 2010; Bahar et al., 2017; Norby et al., 2017). (a) Wood density and leaf turnover rate; and specific leaf area (SLA) against (b) leaf turnover rate; (c) leaf carbon:nitrogen ratio; and (d) mass-based maximum carboxylation capacity. For panel (a), values were aggregated to species to increase sample size, otherwise individual measurements were used. Black line is the fitted standardized major axes, and the equations along with the number of points (n) and squared correlation (R^2) are shown for reference. Values for each PFT are shown in the plot for reference. Grasses are included, but their fitted relationship were carried out separately for the relationships shown in panels (b) and (d).

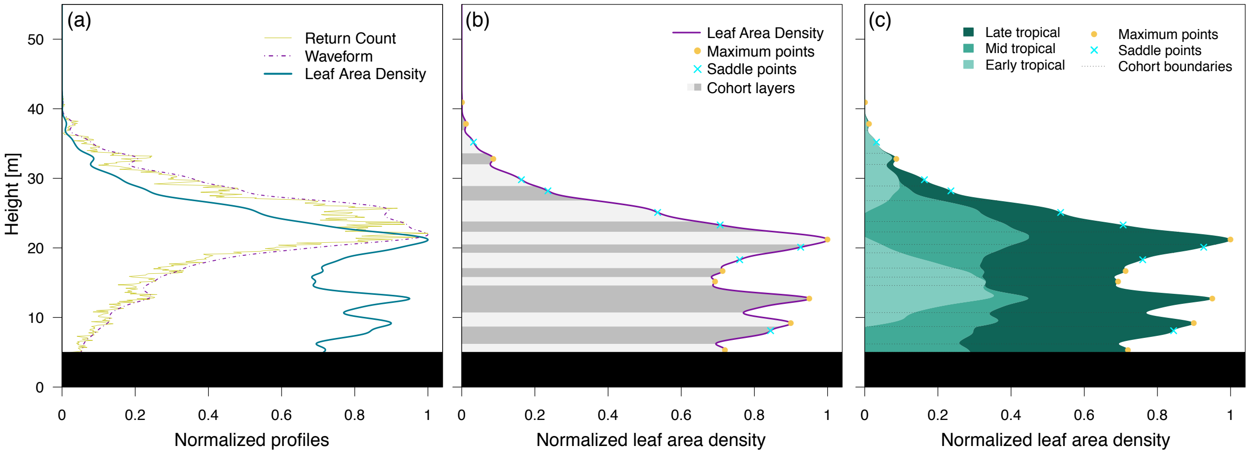

Figure S19. Example of how cohorts are obtained from the vertical distribution of returns, from one 50 × 50 m column at Paracou (GYF). (a) Thin lines: vertical profiles of return counts (X_v; Eq. S26); dot-dashed lines: waveform function (E_v; Eq. S25); thick lines: leaf area density ($\lambda^{\star}$; Eq. S30). (b) Discrete layers based on the curve features of leaf area density (thick line); Circles are the local maximum points and crosses are the saddle points. Discrete cohort layers are shown in alternate background shades. (c) Plant functional type (PFT) and cohort attribution. Cohorts are defined by the cohort layers, and further split by the existing PFTs in each layer. The unscaled leaf area index of each cohort is defined by the integral of the curve between each discrete layer and within each plant functional group. Black rectangles near ground are the bottom layer that is excluded from the cohort attribution.

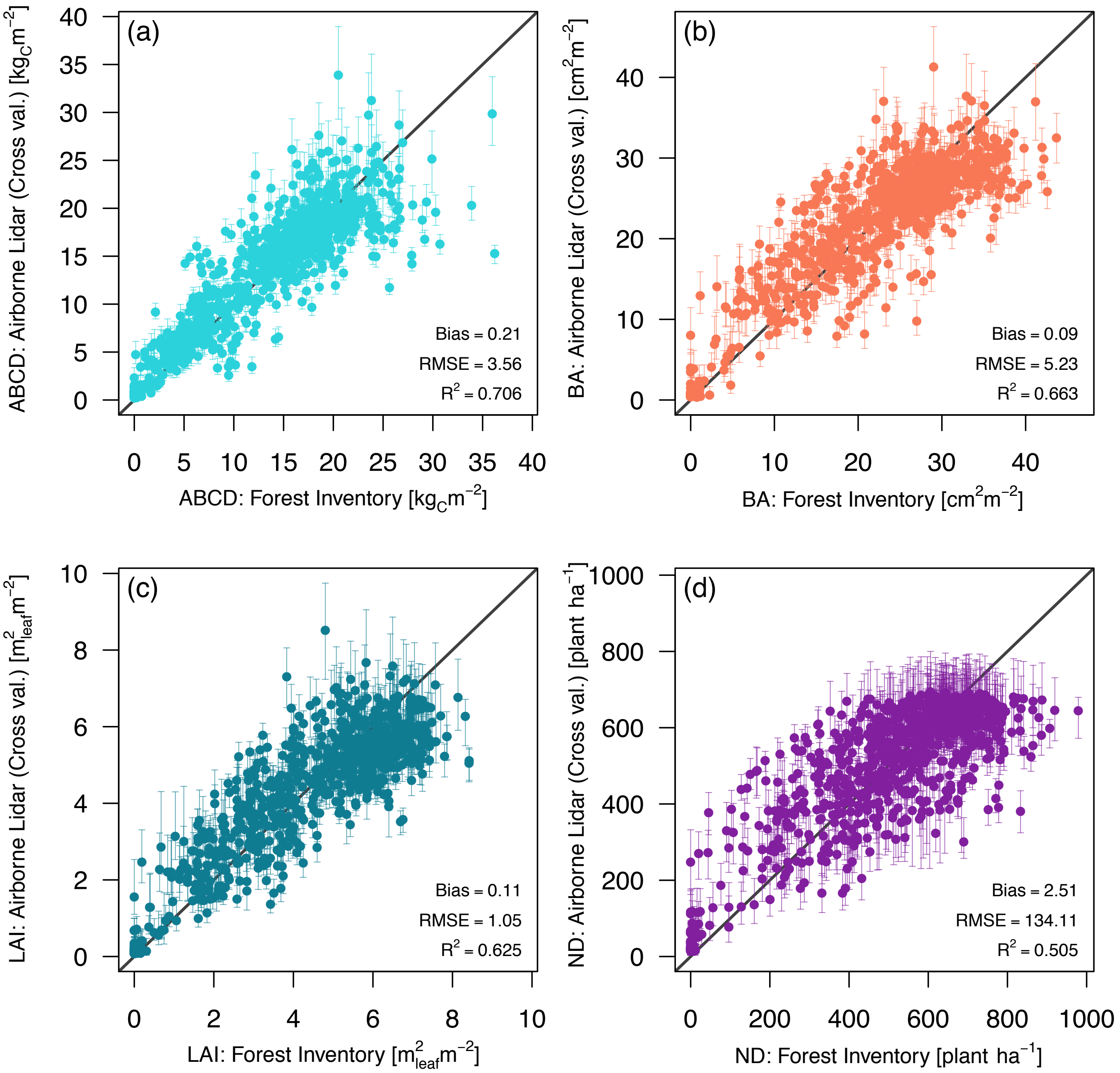

Figure S20. Comparison between forest inventory and airborne-lidar estimates of plot-level properties. (a) aboveground biomass carbon density (ABCD), (b) Basal area (BA), (c) (maximum, allometry-based) leaf area index and (d) stem number density (ND). For the airborne-lidar estimates, we show the average results from cross-validation: for each plot, we averaged all replicates which did not include the plot region in the model training step. Bars correspond to the 95% range of cross-validation predictions. Median bias, root mean square error (RMSE) and adjusted coefficient of determination (R^2_{adj}) for cross-validation predictions are shown for reference.

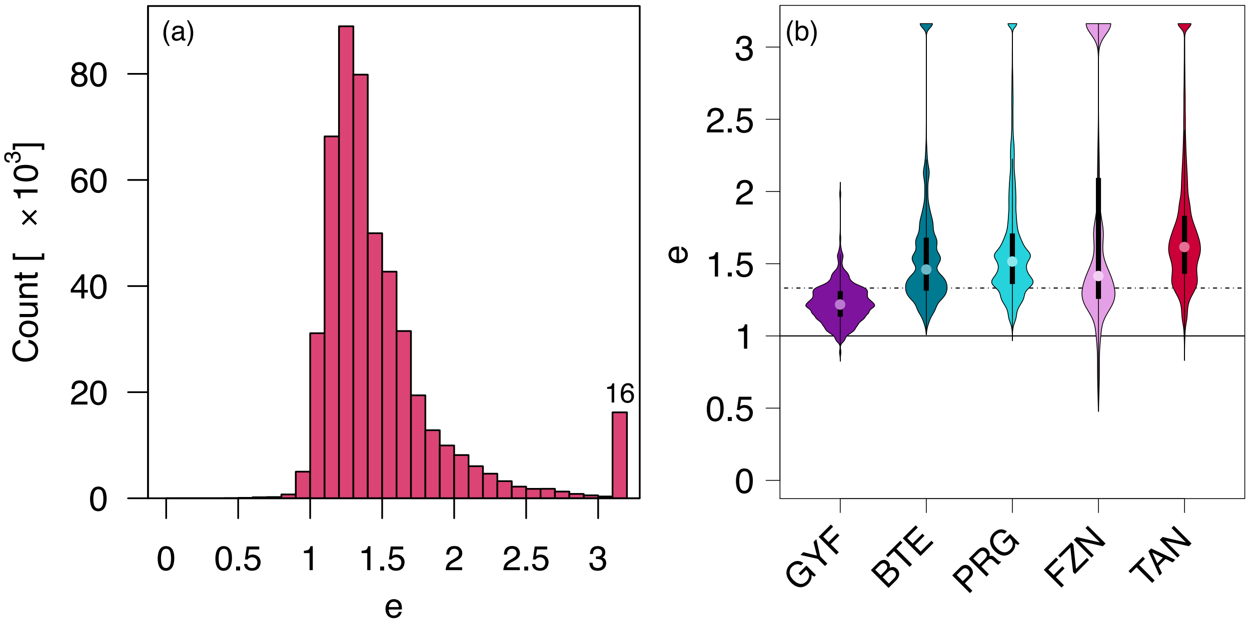

Figure S21. Statistics of the scaling factor e (Equation S55). (a) Histogram of e obtained from all plots and realizations of the regional cross-validation; the x axis was truncated at $\sqrt{10}$ to improve legibility, and the number of replicates exceeding this threshold is shown in the last bar of the histogram. (b) Violin plots for the five study regions: GYF – Paracou, BTE – Belterra, PRG – Paragominas, FZN – Feliz Natal, TAN – Tanguro; dot-dashed line represents the median ($e_{50} = 1.357$) used as the general scaling factor. The distribution was also truncated at $\sqrt{10}$, and the density function at the largest values along the y axis includes all values that exceed $\sqrt{10}$.

Table S1. Summary of model evaluation for eddy covariance tower sites Paracou (GYF) – Intact, Belterra (BTE) – Intact, Tanguro (TAN) – Intact, and Tanguro (TAN), Burned. In all cases, we only used daily averages for those days without gaps in tower observations or estimates. The following metrics are presented: number of daily averages observations (N), bias, root mean square error (RMSE), mean absolute error (MAE), Pearson's correlation coefficient (r). Units for bias, RMSE and MAE are presented in brackets; other metrics are dimensionless.

Variable	Metric	Paracou (GYF), Intact		Belterra (BTE), Intact		Tanguro (TAN), Intact		Tanguro (TAN), Burned	
		Inventory	Lidar	Inventory	Lidar	Inventory	Lidar	Inventory	Lidar
Gross Primary Productivity $\left[\mathrm{kgC\,m^{-2}\,yr^{-1}}\right]$	N	2305	2305	884	884	262	262	245	245
	Bias	0.102	0.316	-0.104	0.313	-0.046	0.394	0.296	0.242
	MAE	0.395	0.476	0.430	0.497	0.673	0.781	0.622	0.575
	RMSE	0.514	0.602	0.529	0.607	0.803	0.976	0.725	0.677
	r	0.832	0.826	0.498	0.528	0.506	0.478	0.455	0.501
Net Ecosystem Productivity $\left[\mathrm{kgC\,m^{-2}\,yr^{-1}}\right]$	N	2305	2305	884	884	262	262	245	245
	Bias	-0.555	-1.719	-0.647	-1.287	-0.745	-0.834	0.149	0.0824
	MAE	1.04	1.98	0.96	1.46	1.22	1.31	1.03	0.971
	RMSE	1.18	2.27	1.13	1.72	1.56	1.60	1.31	1.26
	r	0.407	0.299	0.476	0.489	0.494	0.514	0.574	0.577
Evapotranspiration $\left[\mathrm{mm\,day^{-1}}\right]$	N	3001	3001	932	932	539	539	603	603
	Bias	-0.0077	0.117	0.374	0.541	0.687	0.825	-0.0622	0.174
	MAE	0.45	0.47	0.58	0.65	0.89	1.17	0.90	0.86
	RMSE	0.57	0.58	0.74	0.82	1.08	1.32	1.18	1.13
	r	0.820	0.820	0.767	0.793	0.722	0.681	0.453	0.476
Sensible heat flux $\left[\mathrm{W\,m^{-2}}\right]$	N	2064	2064	930	930	291	291	324	324
	Bias	0.46	-1.16	17.7	16.9	6.84	6.38	11.2	11.0
	MAE	7.49	7.51	17.9	17.0	12.9	13.4	18.3	17.8
	RMSE	9.49	9.42	20.2	19.3	16.7	19.0	21.1	20.5
	r	0.864	0.866	0.767	0.783	0.811	0.754	0.808	0.821
Outgoing shortwave radiation $\left[\mathrm{W\,m^{-2}}\right]$	N	3784	3784	158	158	1039	1039	1318	1318
	Bias	2.182	1.807	0.297	0.067	-0.173	-0.298	0.167	0.280
	MAE	2.34	2.04	1.24	1.23	2.80	2.85	1.83	1.81
	RMSE	2.70	2.41	1.70	1.68	3.43	3.51	2.23	2.20
	r	0.970	0.969	0.932	0.932	0.873	0.868	0.940	0.940
Outgoing longwave radiation $\left[\mathrm{W\,m^{-2}}\right]$	N	3943	3943	396	396	1039	1039	1318	1318
	Bias	13.1	11.8	23.9	21.4	25.5	24.8	-5.6	23.9
	MAE	13.1	11.8	23.9	21.4	25.5	24.8	7.6	23.9
	RMSE	14.3	13.0	24.9	22.2	26.6	26.6	9.4	25.2
	r	0.647	0.658	0.938	0.938	0.891	0.863	0.889	0.889

Table S2. Detailed information of each study region. Density corresponds to the average number of returns per unit area. For plots that used sub-plots to sample individuals with diameter at breast height $D < 35\,\mathrm{cm}$, we provide the size of the sub-plot in parentheses. Ancillary regions and sites used only to establish the statistical models are shown in *italics*.

Region (Code)	Site	Coordinates	Lidar			Inventory		
			Area [ha]	Density [m^{-2}]	Date	Count	Size (Sub-Size) [m × m]	Date
Paracou (GYF)	GFE	5.28°N; 52.93°W	963[a]	22.4[a]	Sep 2013[a]	22	35 × 70 (16); 50 × 50 (6)[b]	Mar 2013
	PRC	5.27°N; 52.93°W				300	50 × 50[c]	Mar 2013
Belterra (BTE)	ANA	3.36°S; 55.00°W	989	20.11	Mar 2017	32	50 × 50	Jul 2015
	EBT	3.18°S; 54.88°W	1004	54.9	Apr 2015	14	50(5) × 50	Nov 2014
	TNF	2.86°S; 54.95°W	1048	19.3	Mar 2017	9	50 × 50	Dec 2015 – Mar 2016
	TSJ	3.13°S; 54.97°W	1012	30.0	Jul–Aug 2013	12	50 × 50	Sep 2013
Paragominas (PRG)	AND	2.55°S; 46.83°W	1000	38.2	Jun 2014	20	50(5) × 50	Aug 2013
	CAU	3.75°S; 48.48°W	1214	28.3	Jul 2012	85	20(2) × 125[d]	Jan–Mar 2012
	PAR	3.32°S; 47.53°W	1003	40.0	Jun 2014	39	20(2) × 125[d]	Mar–Apr 2013
	TAC[e]	2.77°S; 48.52°W	983	24.2	Nov 2013	13	50 × 50	May-Jun 2015
Feliz Natal (FZN)	FN2	11.86°S; 54.19°W	995	30.7	Mar 2016	7	50(5) × 50	Aug 2015
	FNA	12.50°S; 55.01°W	1200	38.3	Aug 2013	20	50 × 50	Oct 2013
	FNC	12.00°S; 54.20°W	903	15.2	Apr 2017	9	50(5) × 50	Aug 2015
	FND	12.27°S; 55.08°W	1099	13.2	Apr 2017	20	50(5) × 50	Aug 2015
Tanguro (TAN)	TGE	13.08°S; 52.38°W	1006[a]	13.1[a]	Aug 2012[a]	72	20(10) × 125[f]	Jun 2012
	TGW	13.09°S; 52.40°W				20	20(2) × 125[d]	Nov 2012
São Félix do Xingu (SFX)	*SX1*	6.41°S; 52.90°W	993	30.1	Aug–Sep 2012	9	40 × 40	Oct 2011
	SX2	6.60°S; 51.79°W	1005	30.1	Aug–Sep 2012	22	40 × 40	Aug 2012
Jamari (JAM)	*JAM*	9.12°S; 63.01°W	1673	31.0	Sep 2013	23	50(5) × 50	Dec 2013
Rio Branco (RBR)	*BON*	9.87°S; 67.29°W	600	33.4	Sep 2013	10	50(10) × 50	Jul 2014
	HUM	9.76°S; 67.65°W	501	66.7	Sep 2013	10	50(10) × 50	Jun–Jul 2014
	TAL	10.26°S; 67.98°W	500	40.7	May 2014	5	50(10) × 50	Jul 2014
Saracá Taquera (FST)	*FST*	1.62°S; 56.22°W	1021	32.9	Aug 2013	19	50(5) × 50	Nov 2013
Manaus (MAO)	*DUC*	2.95°S; 59.94°W	1248	22.7	Feb 2012	25	26(⋆) × 100[g]	Sep 2011

[a] Both sites were covered by the same airborne lidar survey.
[b] Original plot sizes 70 × 70 m (8), 50 × 100 m (1) and 100 × 100m (1), split in 35 × 70 m or 50 × 50 m blocks to be comparable with other areas.
[c] Original plot size 250 × 250 m, split in 50 × 50 m blocks to be comparable with other areas.
[d] Original transect size 20 × 500 m, split in 20 × 125 m blocks to be comparable with other areas.
[e] The lidar survey includes only second-growth forests and forest plantations, which are outside the scope of this study. All plots were located in second-growth forests.
[f] Original transect size 20 × 1500 m, split in 20 × 125 m blocks to be comparable with other areas. Sub-sampling was applied to trees with $D \leq 20\,\mathrm{cm}$.
[g] Sampling effort varied depending on the D, following Hunter, Keller, Vitoria, and Morton (2013). Nominal plot size defined from the largest surveyed tree ($D = 128.5\,\mathrm{cm}$).

:

Table S3. Configuration and parameters used in the simulations and described in Text S2. For parameters that are specific to each plant functional type (PFT), we use the format (x_{C4G}; x_{ETR}; x_{MTR}; x_{LTR}), for C_4 grasses, early-, mid-, and late-successional tropical trees, respectively.

**Process**	**Method**	
Integration scheme	4th order Runge-Kutta	
Soil bottom boundary condition	Free drainage	
Leaf phenology	Evergreen	
**Parameter**	**Value**	**Units**
Biophysics time step	240	s
Number of soil layers	16	—
Depth of the deepest soil layer	10.50	m
Depth of the shallowest soil layer	0.04	m
Biomass:carbon ratio (β, all tissues)	2.0	$\mathrm{kg\,kgC^{-1}}$
Fine-root:leaf ratio (q_R)	1.0	$\mathrm{kg_{Root}\,kg_{Leaf}^{-1}}$
Empirical parameter (η_c; Equations S7 and S8)	0.886	—
Leaf (η_c; Equations S7 and S8)	0.886	—
Leaf:sapwood area ratio ($A_{L:S}$, Equation S7)	13513	$\mathrm{m_{Leaf}^2\,m_{Sapwood}^{-2}}$
Leaf:bark area ratio ($A_{L:B}$, Equation S8)	292523	$\mathrm{m_{Leaf}^2\,m_{Bark}^{-2}}$
Aboveground fraction (f_{AG})	0.7	—
Curvature parameter (φ)	0.7	—
Quantum yield of photosystem II (γ_{PSII})	0.85	—
Q_{10} factor for carboxylation (Q_V)	2.43	—
Q_{10} factor for electron transport (Q_J)	1.81	—
ε_J – Equation (S23)	1.766	—
$\varepsilon_{\mathrm{TP}}$ – Equation (S24)	0.110	—
Parameter f_c – Equation (S19)	0.3	—
Parameter f_h – Equation (S19)	0.6	—
Parameter T_c – Equation (S19)	288.15	K
Parameter T_h – Equation (S19)	310.65	K
**PFT-dependent parameter**	**Value**	**Units**
Wood density	(–; 0.45; 0.62; 0.79)	$\mathrm{g\,cm^{-3}}$
Bark density	(–; 0.44; 0.46; 0.45)	$\mathrm{g\,cm^{-3}}$
Specific leaf area	(27.6; 26.2; 19.7; 14.6)	$\mathrm{m_{Leaf}^2\,kgC^{-1}}$
Leaf turnover rate	(2.00; 1.56; 0.80; 0.40)	$\mathrm{yr^{-1}}$
Maximum carboxylation rate ($V_{c15}^{\max}$)	(21.2; 20.3; 17.3; 14.6)	$\mu\mathrm{mol\,m^{-2}\,s^{-1}}$
Leaf carbon:nitrogen ratio	(21.2; 22.1; 28.0; 36.0)	$\mathrm{kgC\,kgN^{-1}}$

Table S4. Summary goodness-of-fit statistics for fitted models for above-ground biomass carbon density (ABCD), basal area (BA), (maximum, allometry-based) leaf area index (LAI) and stem number density (ND), both for the full model (*Full*; all plots used for calibration) and the cross-validation (*X-Val*; the median statistics obtained from 1000 hierarchical bootstrap replicates (goodness-of-fit were assessed from plots in regions not included in the model training stage). The 68% range (equivalent to $\pm 1\sigma$ if the distribution was Gaussian) relative to the median is also shown. Bias, mean absolute error (MAE) and root mean square error (RMSE) are show in percentage relative to the average value of all plots (inventory-based), to simplify comparison across properties. The other statistics are: adjusted coefficient of determination (R^2_{adj}); Kolmogorov-Smirnov statistics (D_{KS}) and *p-value* (p_{KS}).

Statistics	ABCD		BA		LAI		ND	
	Full	X-Val	Full	X-Val	Full	X-Val	Full	X-Val
%Bias	0.0	$1.5^{+5.5}_{-5.6}$	0.0	$0.4^{+6.5}_{-7.4}$	0.0	$2.4^{+5.8}_{-12.6}$	0.0	$0.5^{+6.3}-6.1$
%MAE	17.8	$18.9^{+4.2}_{-3.1}$	15.8	$17.5^{+4.5}_{-3.0}$	15.7	$18.4^{+3.0}_{-2.6}$	18.2	$20.7^{+2.7}_{-4.1}$
%RMSE	25.2	$26.6^{+5.4}_{-4.9}$	20.9	$23.1^{+3.9}_{-3.9}$	20.7	$23.3^{+3.2}_{-2.8}$	24.1	$26.7^{+3.1}_{-5.1}$
R^2_{adj}	0.779	$0.706^{+0.080}_{-0.209}$	0.754	$0.66^{+0.10}_{-0.30}$	0.79	$0.63^{+0.13}_{-0.27}$	0.65	$0.50^{+0.18}_{-0.34}$
D_{KS}	0.049	$0.120^{+0.068}_{-0.045}$	0.086	$0.151^{+0.078}_{-0.052}$	0.087	$0.172^{+0.158}_{-0.062}$	0.18	$0.20^{+0.10}_{-0.06}$
p_{KS}	0.28	$0.066^{+0.363}_{-0.065}$	0.005	$0.018^{+0.245}_{-0.018}$	0.004	$0.013^{+0.230}_{-0.013}$	0.0000	$0.0017^{+0.0628}_{-0.0017}$
